# Effectiveness of oral and inhaled repurposed and antiviral pharmaceutical treatments for acute SARS-CoV-2 in outpatient settings: a systematic review of 124 randomized trials

**DOI:** 10.1186/s12879-026-13901-7

**Published:** 2026-07-23

**Authors:** Banafshe Hosseini, Lesley Anne Pablo, Jolanta Piszczek, Yoav Keynan, Peter Daley, Becky Skidmore, Andrew D. Pinto

**Affiliations:** 1 Upstream Lab, MAP Centre for Urban Health Solutions, Li Ka Shing Knowledge Institute, St. Michael’s Hospital, Unity Health Toronto, Toronto, ON Canada; 2 Department of Family and Community Medicine, Temerty Faculty of Medicine, University of Toronto, Toronto, ON Canada; 3 Division of Clinical Public Health & Institute for Health Policy, Management and Evaluation, Dalla Lana School of Public Health, University of Toronto, Toronto, ON Canada; 4 Faculty of Pharmaceutical Sciences, University of British Columbia, Vancouver, BC Canada; 5 Department of Medical Microbiology and Infectious Diseases, University of Manitoba, Winnipeg, MB Canada; 6 Max Rady College of Medicine, University of Manitoba, Winnipeg, MB Canada; 7 Division of Infectious Diseases, Faculty of Medicine, Memorial University of Newfoundland, St. John’s, Newfoundland and Labrador Canada; 8 Independent Information Specialist, Ottawa, ON Canada; 9 Department of Family and Community Medicine, St. Michael’s Hospital, Toronto, ON Canada

**Keywords:** COVID-19, SARS-CoV-2, Outpatient therapeutics, Antivirals, Drug repurposing, Randomized controlled trials, Systematic review

## Abstract

**Background:**

Outpatient therapeutics for SARS-CoV-2 remain critical to reducing disease severity, preventing hospitalization, and mitigating long-term complications, yet evidence across trials has been fragmented and evolving.

**Objective:**

To systematically identify and evaluate the effectiveness of oral or inhaled pharmaceutical treatments, including repurposed and antiviral therapies, for acute SARS-CoV-2 infection in adult patients managed in outpatient settings.

**Methods:**

Comprehensive peer-reviewed searches were conducted in several databases for studies published by March 2024. We included randomized controlled trials evaluating oral or inhaled therapeutics administered to adults with acute SARS-CoV-2 infection in outpatient settings and excluded non-randomized and observational studies. Outcomes of interest included severe clinical outcomes. Random-effects meta-analyses were performed where feasible.

**Results:**

A total of 124 randomized controlled trials evaluating 137 therapeutics across 35 countries were included. Most interventions were repurposed agents (*n* = 88), with 33 antiviral and three combination regimens. Molnupiravir shortened time to recovery and reduced hospitalization in a subgroup pool of unvaccinated, higher-risk outpatients (OR 0.68, 95% CI 0.52–0.89), although this effect attenuated when mostly vaccinated patients was included (OR 0.83, 95% CI 0.58–1.19). Inhaled budesonide and metformin showed modest or setting-specific benefits, while most repurposed drugs, including fluvoxamine, ivermectin, and hydroxychloroquine, had no consistent clinical impact. Safety profiles were class-typical with no new signals identified.

**Conclusions:**

Most repurposed and antiviral therapies evaluated in outpatient COVID-19 trials showed no consistent benefit for severe clinical outcomes. Modest or setting-specific effects were observed for inhaled budesonide, metformin, and molnupiravir, with molnupiravir’s effect on hospitalization most evident in unvaccinated, higher-risk outpatients and unreplicated for mortality.

**Clinical trial number:**

Not applicable.

**Supplementary Information:**

The online version contains supplementary material available at https://doi.org/10.1186/s12879-026-13901-7.

## Background

Since its emergence in late 2019, SARS-CoV-2 has caused substantial global morbidity and mortality, with hundreds of millions of confirmed infections and millions of deaths worldwide [1]. Although widespread vaccination has substantially reduced severe outcomes, SARS-CoV-2 remains a major cause of morbidity and mortality, with recurrent waves driven by new variants, waning immunity, and persistent inequities in vaccine access and uptake [2]. Identifying accessible, affordable outpatient treatments that can reduce symptom burden, prevent progression, and shorten infectious periods, therefore, remains a global public health priority.

The therapeutic landscape has evolved rapidly. Early in the pandemic, inpatient trials identified systemic therapies that significantly reduced mortality in hospitalized patients [3]. In contrast, the outpatient setting has lagged, even though most cases are managed outside of hospitals. Several oral antivirals (such as molnupiravir [4–6], favipiravir [7, 8], bemnifosbuvir [9], leritrelvir [10], interferon-β (SNG001) [11, 12], and lopinavir/ritonavir [13]) and inhaled agents (such as budesonide [14], ciclesonide [15]) have been evaluated, but evidence remains fragmented, with trials often underpowered, inconsistent in design, or limited to specific populations and viral variants. Repurposed drugs, including hydroxychloroquine, ivermectin, and fluvoxamine [16–21], have been widely tested but with conflicting or inconclusive results, raising questions about their true clinical utility.

Previous efforts, including the WHO living network meta-analysis of COVID-19 therapeutics [22], the BMJ living review [23], and the more recent meta-analysis of 61 trials by Sullivan et al. [24], were instrumental in shaping treatment guidelines during the acute phases of the pandemic. However, these reviews are now outdated, with dozens of outpatient randomized controlled trials (RCTs) published since their last update. This systematic review, therefore, aims to provide a comprehensive synthesis of oral and inhaled therapeutics for non-hospitalized COVID-19 patients. By collating and critically appraising this expanding body of evidence, we seek to inform treatment recommendations, highlight research gaps, and strengthen preparedness for future respiratory pandemics.

## Methods

This systematic review was registered on Open Science Framework (Registration number: https://osf.io/s53bd/overview) and prepared following the Preferred Reporting Items for Systematic Reviews (PRISMA) guidelines [25].

### Search strategy

An Information Specialist (BS) developed and tested the search strategies through an iterative process in consultation with the review team. Another senior information specialist peer reviewed the MEDLINE strategy prior to execution using the Peer Review of Electronic Search Strategies (PRESS) checklist [26].

Using the multifile option and deduplication tool on the Ovid platform, we searched Ovid MEDLINE^®^ ALL, Embase Classic+Embase, and International Pharmaceutical Abstracts. We also searched the Cochrane Central Register of Controlled Trials (CENTRAL) on the Wiley platform, CINAHL on Ebsco, and the Web of Science core databases (excluding the two conference databases). We applied a combination of controlled vocabulary (e.g., “COVID-19,” “COVID-19 Drug Treatment,” “Drug Therapy”) and keywords (e.g., “SARS-CoV-2,” “Paxlovid,” “pharmacotherapy”), adjusting the vocabulary and syntax as necessary across the databases. We applied an amended version of the 2008 Cochrane highly sensitive search strategy (sensitivity- and precision-maximizing version) in all databases but CENTRAL, which is prefiltered for randomized and controlled clinical trials. We did not restrict by language but did limit the publication year from 2019 to the present and removed animal-only records, conference abstracts, opinion pieces, and other unwanted publication types, where possible. We performed all searches on September 3, 2023, and updated them on December 18, 2023, and March 26, 2024. Results were downloaded and de-duplicated using EndNote 9.3.3 (Clarivate Analytics) and uploaded to Covidence systematic review software (Veritas Health Innovation) for final deduplication and screening. The full search strategy can be found in Supplementary File 1.

### Eligibility criteria

This review included randomized controlled trials conducted in outpatient settings that investigated pharmaceutical COVID-19 therapeutics administered orally or through inhalation. The population of interest included human adults aged 18 years and older infected with SARS-CoV-2 from all genders and ethnicities. Eligible studies compared treatments against any other active comparator or treatment, placebo, active placebo, no treatment, or standard care. Other study designs, such as non-randomized trials, observational studies, case-studies, animal studies, in vitro studies, conference abstracts, opinion papers, preprints, reviews, and studies with no control group or on non-pharmaceutical therapeutics or involving intravenous interventions were excluded. Studies published in languages other than English were also excluded at the full-text eligibility stage.

Primary outcomes in this review include all-cause mortality, any hospital admission or duration, intensive care unit (ICU) admission or duration or mechanical ventilation, any emergency department (ED) admission or re-admissions, time to recovery, severity and duration of symptoms, viral clearance or viral load, and prevalence of long COVID, defined as persistent symptoms lasting over 12 weeks.

### Screening process

Covidence was used to perform screening. Several members of the research team (PK, NM, AO, ZP, USR, ESA, NS, IF, ASR) were trained on the inclusion and exclusion criteria of studies to perform first- and second-level screening of citations. First-level screening involved title and abstract screening of studies while second-level screening involved a thorough assessment of the full texts of studies included through first-level screening. Each study was independently assessed by two reviewers during first- and second-level screening. Screening conflicts on citation inclusion and exclusion were brought to the study team and were resolved by a third reviewer. Second-level screening only involved studies that were deemed eligible during first-level screening. All studies deemed eligible during full-text screening were included in the study for extraction and synthesis.

### Data collection and synthesis

Data were extracted from all studies included after full-text screening. One reviewer completed data extraction for each study, and a second team member independently checked the extracted data for accuracy and completeness. Extracted variables included study setting, sample size by treatment arm, participant age and sex, vaccination status, prior SARS-CoV-2 infection, common comorbidities, and whether the study focused on a specific population defined by age, risk status, or other eligibility criteria. Intervention-level data included the therapeutic agent, dose, duration, route of administration, comparator group, outcomes assessed, effect estimates, study limitations, and authors’ conclusions.

We grouped studies by therapeutic category, route of administration, and outcome. Where sufficiently comparable data were available, we conducted meta-analyses for binary clinical outcomes, including hospitalization, ICU admission, and all-cause mortality. For these outcomes, treatment effects were summarized using relative measures of effect with corresponding 95% confidence intervals. We pooled relative effect estimates on the log scale. Because severe outcomes were rare, ORs, RRs, and HRs were considered comparable. Random-effects meta-analyses were performed in Python 3 (NumPy, SciPy), with the Haldane-Anscombe 0.5 continuity correction applied to studies with zero cells. Forest plots were produced with Matplotlib. Meta-analysis was not conducted for outcomes with substantial heterogeneity in outcome definitions, timing of assessment, measurement approaches, or reporting formats. These outcomes were summarized narratively [27].

### Risk of bias and quality assessment

Risk of bias assessments were completed using Cochrane’s Risk of Bias 2 (RoB 2) tool [28, 29] with figures generated using the *robvis* tool [30]. Each study deemed eligible during full-text screening was independently assessed by two reviewers, with conflicts resolved by a third reviewer.

### Patient and public involvement

Patients and members of the public were not involved in the design, conduct, reporting, or dissemination plans for this review.

## Results

### Search results

**Fig. 1:**
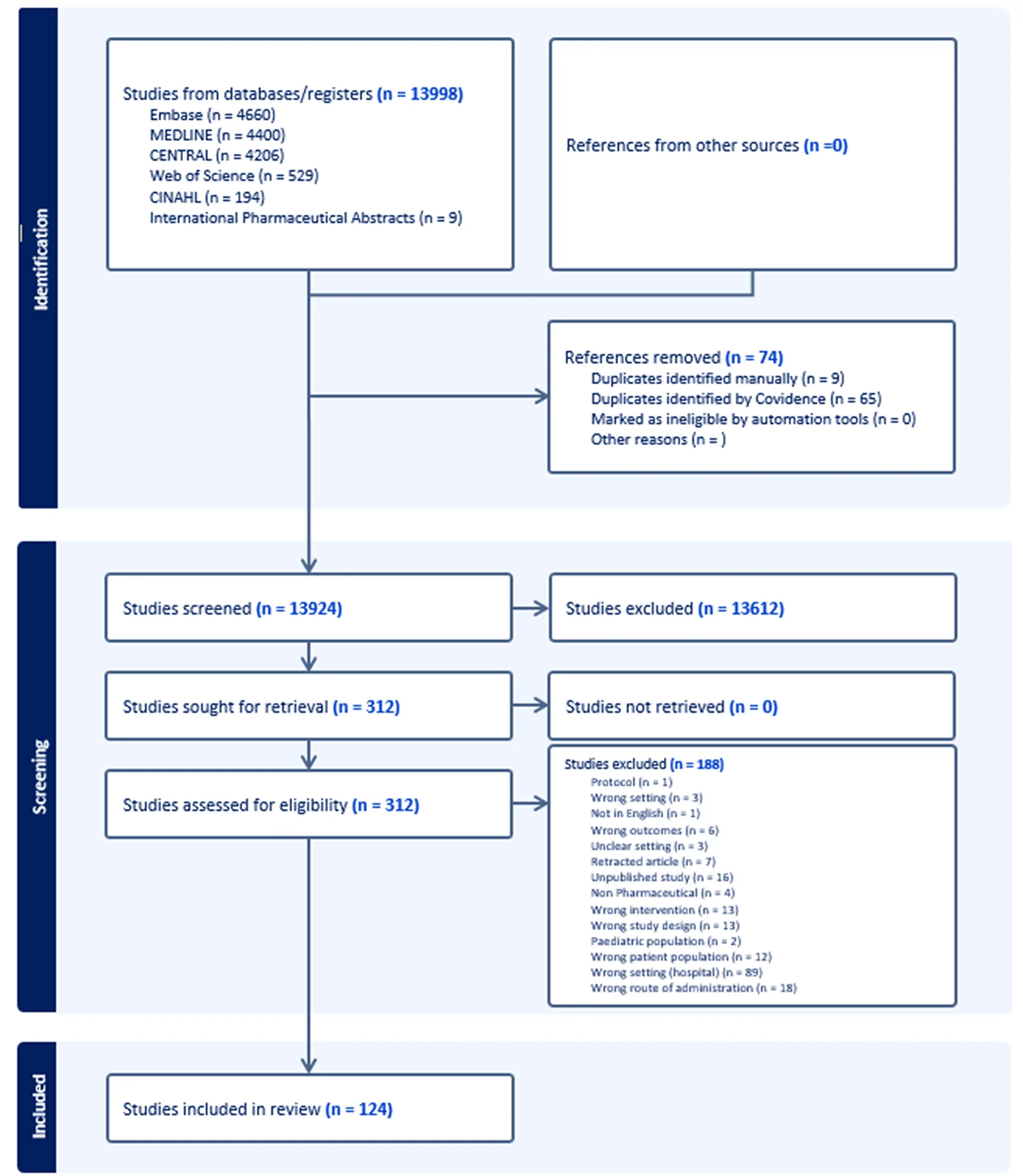
PRISMA flow diagram

The base search captured 18,675 citations with 12,340 remaining after de-duplication through EndNote and Covidence. The first update captured 1,782 citations with 848 remaining, while the second update, in turn, captured 1,880 citations with 745 remaining after de-duplication. We screened 13,998 citations based on title and abstract, with 13,612 and 74 citations classified as irrelevant and as duplicates, respectively. We assessed 312 full texts of studies, with 188 studies excluded based on the inclusion criteria. A total of 124 studies were included in the review (Fig. 1).

### Study characteristics

We identified 124 outpatient randomized controlled trials evaluating 137 therapeutic interventions across 35 countries (Table 1). Of these, 88 assessed repurposed agents, 33 evaluated antivirals, and three tested combination regimens. The most frequently studied treatments were hydroxychloroquine, ivermectin, molnupiravir, favipiravir, and fluvoxamine. Most trials were placebo-controlled (*n* = 80), with fewer using usual care (*n* = 15) or active comparators (*n* = 10). Studies were conducted globally, with the largest contributions from Iran (*n* = 13), Brazil (*n* = 13), the United States (*n* = 31), and the United Kingdom (*n* = 11).

**Table 1:** Characteristics of included studies

Characteristics	*N* (%)
**Year of publication**	
2019	0 (0.0%)
2020	4 (3.2%)
2021	30 (24.2%)
2022	36 (29.0%)
2023	46 (37.1%)
2024	8 (6.5%)
**Region**	
Africa	7 (5.6%)
Asia	16 (12.9%)
Australia	1 (0.8%)
Central and South America	17 (13.7%)
Europe	25 (20.2%)
Middle East	15 (12.1%)
North America	34 (27.4%)
Multinational/ global	9 (7.3%)
**Type of administration**	
Oral	109 (87.9%)
Inhaled	12 (9.7%)
Oral and inhaled	3 (2.4%)

### Risk of bias

**Fig. 2:**
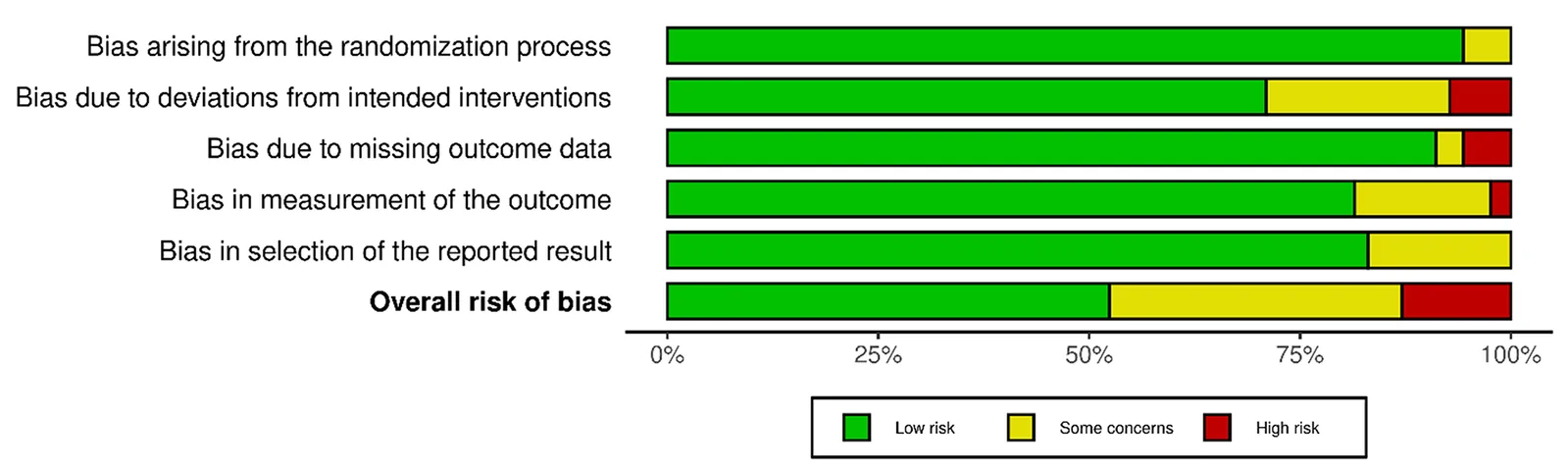
Risk of bias 2 summary

Across 124 outpatient trials, 52.4% were rated low risk overall, 34.7% had some concerns, and 12.9% were high risk (Fig. 2). Most trials (94.4%) had low risk for randomization, while bias from deviations from intended interventions was most frequent (71.0% low, 21.8% some concerns, 7.2% high). Missing outcome data was low risk in 91.1% of studies, and measurement bias was low in 81.4%. Selective reporting bias was low in 83.1% of trials. The complete RoB 2 assessment can be found in Supplementary File 2.

### Time to recovery

We identified 26 outpatient RCTs evaluating repurposed or antiviral agents with time-to-recovery endpoints (time to first or sustained recovery, “time unwell,” or closely related symptom measures). Of these, 20 tested repurposed therapies and six evaluated antivirals (Supplementary File 3, Table 1a and b). Repurposed agents with no consistent benefit included antibacterials (azithromycin [31], doxycycline [32]) and several others (colchicine [33], camostat [21], fluvoxamine regimens [34, 35], ciclesonide [15], fluticasone furoate [36]. In contrast, inhaled budesonide improved recovery in both a small RCT [14] and the PRINCIPLE platform trial [37], while ciclesonide had no effect [15]. Dutasteride showed higher early remission rates compared with placebo in a small trial [38]. Ivermectin showed no benefit in ACTIV-6 but a modest signal for faster recovery in the UK platform trial [39–41]. Among antivirals, favipiravir showed no effect on recovery [42]. Molnupiravir consistently shortened recovery across three RCTs, including a large UK platform analysis estimating ~ 3.5-day acceleration [4–6], and leritrelvir also improved time to recovery in a large trial [10]. Interferon-β (SNG001) did not show benefit in two outpatient trials [11, 12], tafenoquine also failed to demonstrate improvements [43], and a small three-arm open-label study of N-acetylcysteine and bromhexine suggested shorter mean recovery in the active arms versus usual care but lacked a placebo control and requires confirmation [44].

### Emergency department admission

We identified nine outpatient randomized trials that reported ED visits as an outcome (Supplementary File 3, Table 2a and b). Of these, seven tested repurposed treatments (such as metformin, fluvoxamine, losartan) and 2 evaluated antivirals, including Favipiravir [7], and Molnupiravir [45].

Among repurposed therapies, most showed no consistent benefit. Acebilustat [46] and losartan [47] did not reduce ED visits, and fluvoxamine produced mixed results, showing no effect in smaller trials [19, 48] but a reduction in prolonged ED stays in the large TOGETHER trial [49]. Budesonide lowered urgent care or hospital admission in a UK trial [14], while metformin was the only metabolic agent associated with reduced ED visits [19]. Among antivirals, favipiravir showed no benefit [7], whereas molnupiravir reduced acute care visits in a multinational trial [45].

### Hospital admission or duration

Across 76 outpatient randomized trials, 56 evaluated repurposed treatments, 17 assessed antivirals, and 3 tested combination regimens including both categories (Supplementary File 3, Table 3a- c). Most repurposed agents did not significantly reduce hospital admissions or shorten hospital stay. This included antibiotics, anticoagulants, renin–angiotensin–aldosterone system inhibitors, antiparasitic agents, and other anti-inflammatory or metabolic drugs [40, 41, 47, 50–52]. Small studies of budesonide [14], sulodexide [53], methylprednisolone [54], and upamostat [55] suggested potential benefit, but effects were modest and inconsistently replicated across trials. Trials of hydroxychloroquine, colchicine, camostat, ivermectin, fluvoxamine, and metformin were largely null in larger or more robust studies [16–21, 39, 41].

Among antivirals, results were similarly mixed. Molnupiravir reduced hospitalization or death in its pivotal registration trial [56] but not in the large pragmatic PANORAMIC study [57] Other antivirals, including favipiravir [7, 8], bemnifosbuvir [9], leritrelvir [10], interferon-β (SNG001) [11, 12], and lopinavir/ritonavir [13], showed no consistent benefit on hospital admissions in generally low-risk outpatient populations. Mixed antiviral-repurposed combinations, such as hydroxychloroquine ± lopinavir/ritonavir [58] and nitazoxanide [59], also failed to reduce hospitalization rates. Antibacterials such as azithromycin [31] and doxycycline [32] also exhibited null effects.

**Fig. 3:**
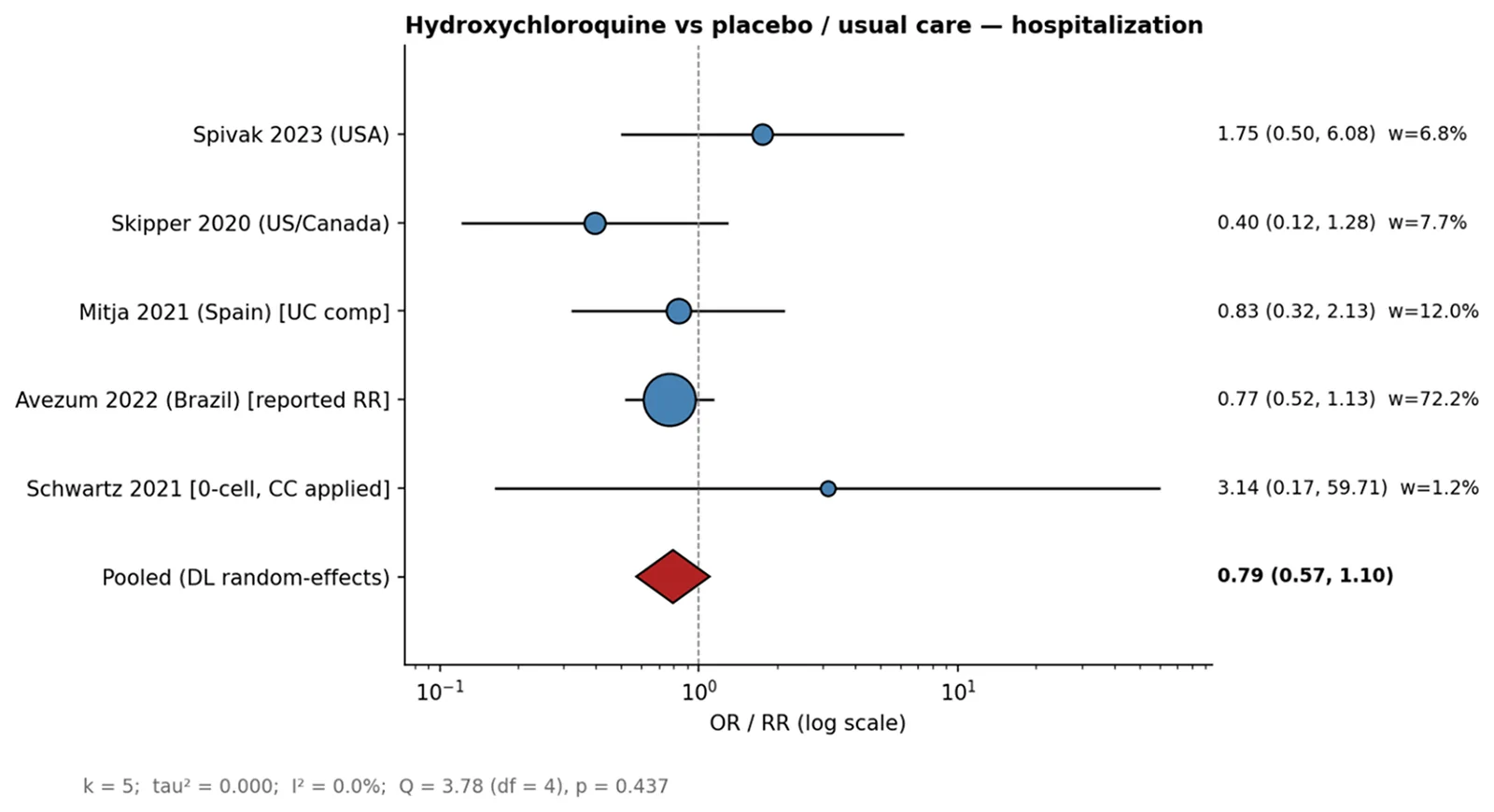
Hydroxychloroquine versus placebo/usual care for hospitalization. Forest plot of randomized trials comparing hydroxychloroquine with placebo or usual care for hospitalization. Forest plots show study-specific effect estimates and 95% confidence intervals for each comparison. Blue circles represent individual study estimates, with the size of the circle proportional to the study weight in the meta-analysis. Horizontal lines represent 95% confidence intervals. The red diamond represents the pooled random-effects estimate, and the width of the diamond represents the 95% confidence interval. The vertical dashed line at 1.0 represents the line of no effect. Estimates to the left of 1.0 favour the intervention for adverse clinical outcomes, while estimates to the right favour placebo, usual care, or standard of care. The x-axis is presented on a logarithmic scale. OR=odds ratio; RR=risk ratio; HR=hazard ratio; CI=confidence interval; DL=DerSimonian–Laird random-effects model; I²=percentage of total variability due to between-study heterogeneity; τ²=between-study variance; Q=Cochran’s heterogeneity statistic; df=degrees of freedom; p = p-value for the heterogeneity test; SoC=standard of care; UC=usual care; CC=continuity correction; MV=mechanical ventilation; IMV=invasive mechanical ventilation; NIV = non-invasive ventilation

**Fig. 4:**
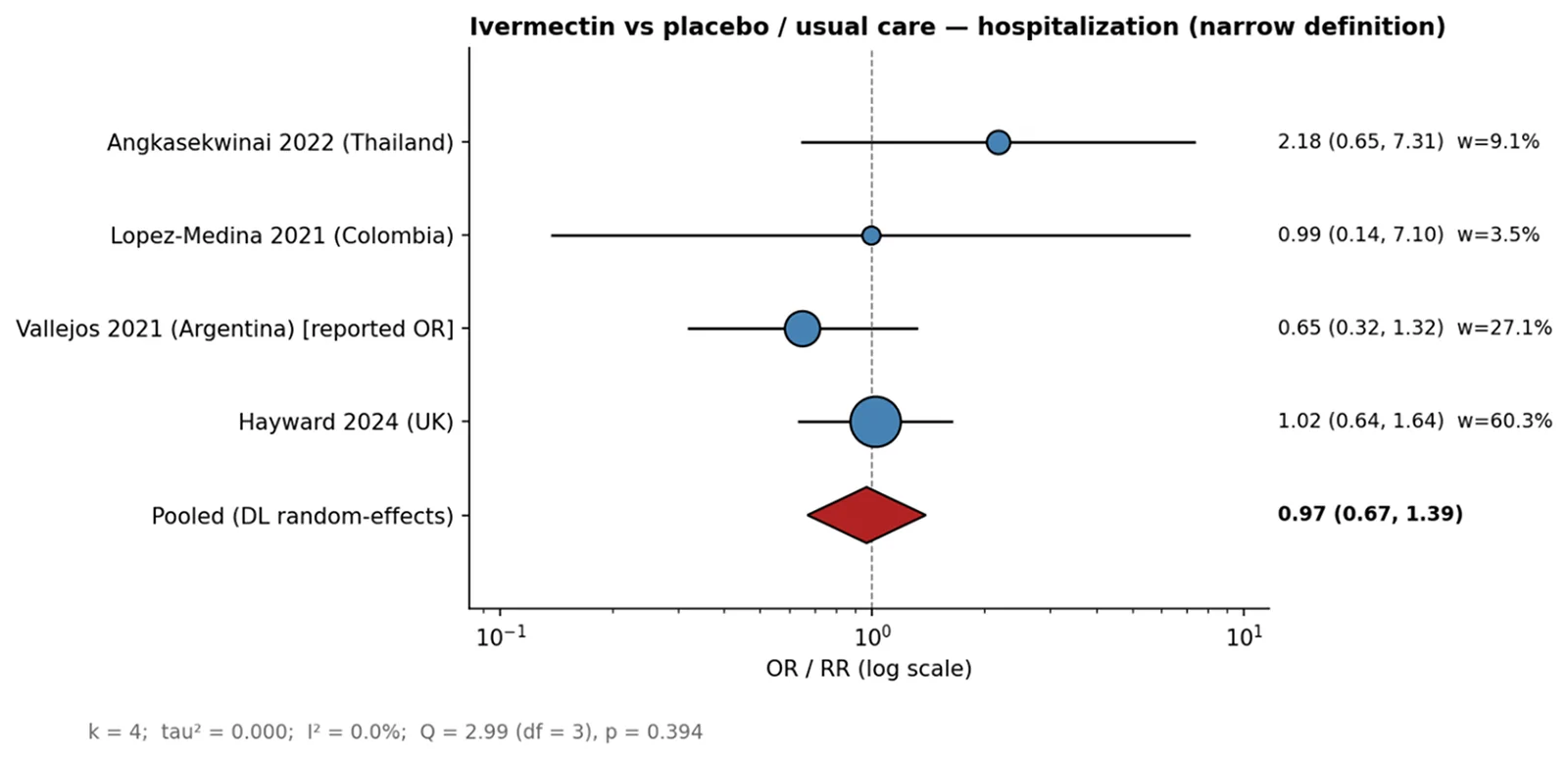
Ivermectin versus placebo/usual care for hospitalization. Forest plot of randomized trials comparing ivermectin with placebo or usual care for hospitalization using the narrow hospitalization definition. Forest plots show study-specific effect estimates and 95% confidence intervals for each comparison. Blue circles represent individual study estimates, with the size of the circle proportional to the study weight in the meta-analysis. Horizontal lines represent 95% confidence intervals. The red diamond represents the pooled random-effects estimate, and the width of the diamond represents the 95% confidence interval. The vertical dashed line at 1.0 represents the line of no effect. Estimates to the left of 1.0 favour the intervention for adverse clinical outcomes, while estimates to the right favour placebo, usual care, or standard of care. The x-axis is presented on a logarithmic scale. OR=odds ratio; RR=risk ratio; HR=hazard ratio; CI=confidence interval; DL=DerSimonian–Laird random-effects model; I²=percentage of total variability due to between-study heterogeneity; τ²=between-study variance; Q=Cochran’s heterogeneity statistic; df=degrees of freedom; p = p-value for the heterogeneity test; SoC=standard of care; UC=usual care; CC=continuity correction; MV=mechanical ventilation; IMV=invasive mechanical ventilation; NIV = non-invasive ventilation

**Fig. 5:**
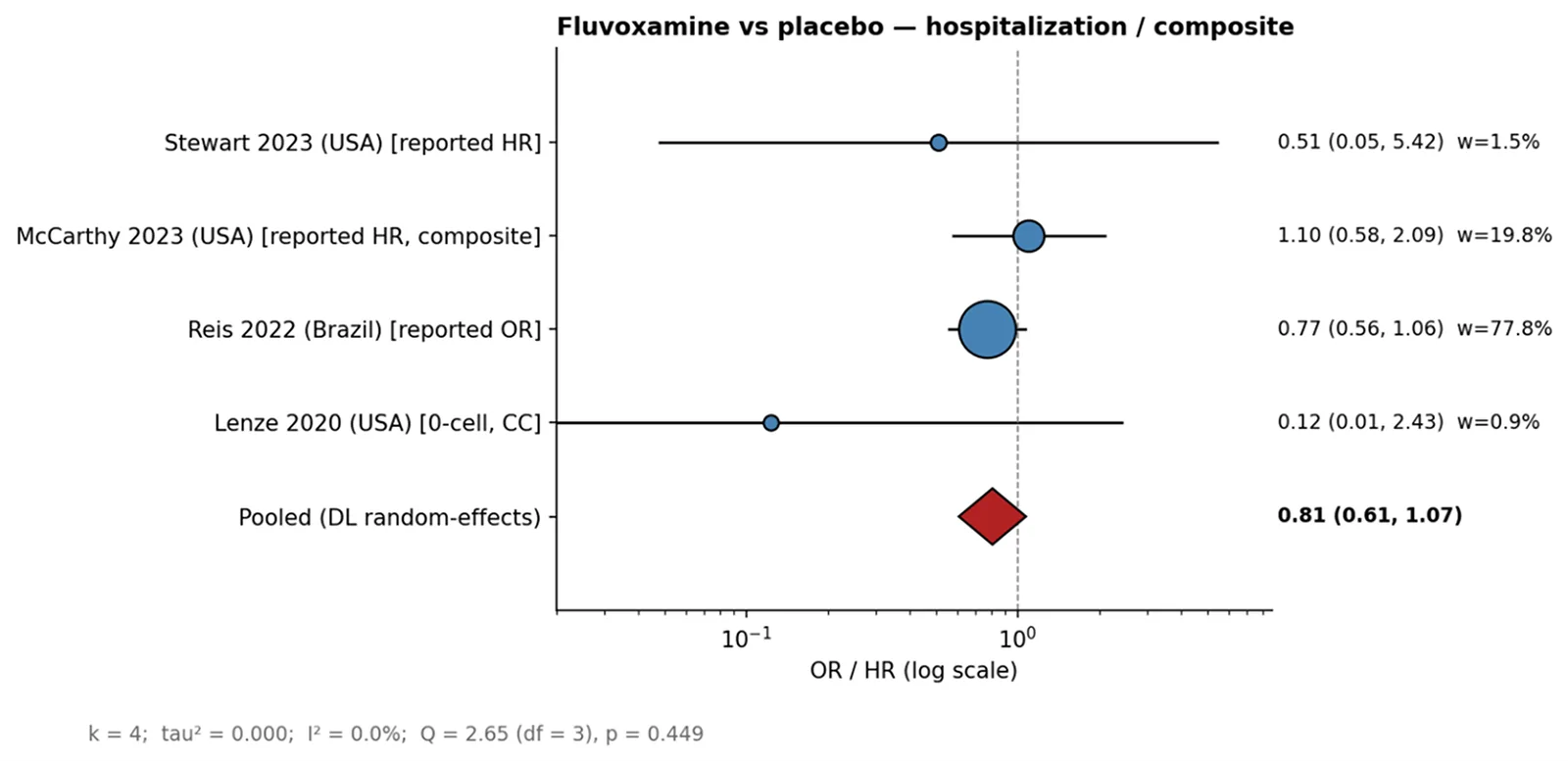
Fluvoxamine versus placebo for hospitalization. Forest plot of randomized trials comparing fluvoxamine with placebo for hospitalization or hospitalization-related composite outcomes. Forest plots show study-specific effect estimates and 95% confidence intervals for each comparison. Blue circles represent individual study estimates, with the size of the circle proportional to the study weight in the meta-analysis. Horizontal lines represent 95% confidence intervals. The red diamond represents the pooled random-effects estimate, and the width of the diamond represents the 95% confidence interval. The vertical dashed line at 1.0 represents the line of no effect. Estimates to the left of 1.0 favour the intervention for adverse clinical outcomes, while estimates to the right favour placebo, usual care, or standard of care. The x-axis is presented on a logarithmic scale. OR=odds ratio; RR=risk ratio; HR=hazard ratio; CI=confidence interval; DL=DerSimonian–Laird random-effects model; I²=percentage of total variability due to between-study heterogeneity; τ²=between-study variance; Q=Cochran’s heterogeneity statistic; df=degrees of freedom; p = p-value for the heterogeneity test; SoC=standard of care; UC=usual care; CC=continuity correction; MV=mechanical ventilation; IMV=invasive mechanical ventilation; NIV = non-invasive ventilation

**Fig. 6:**
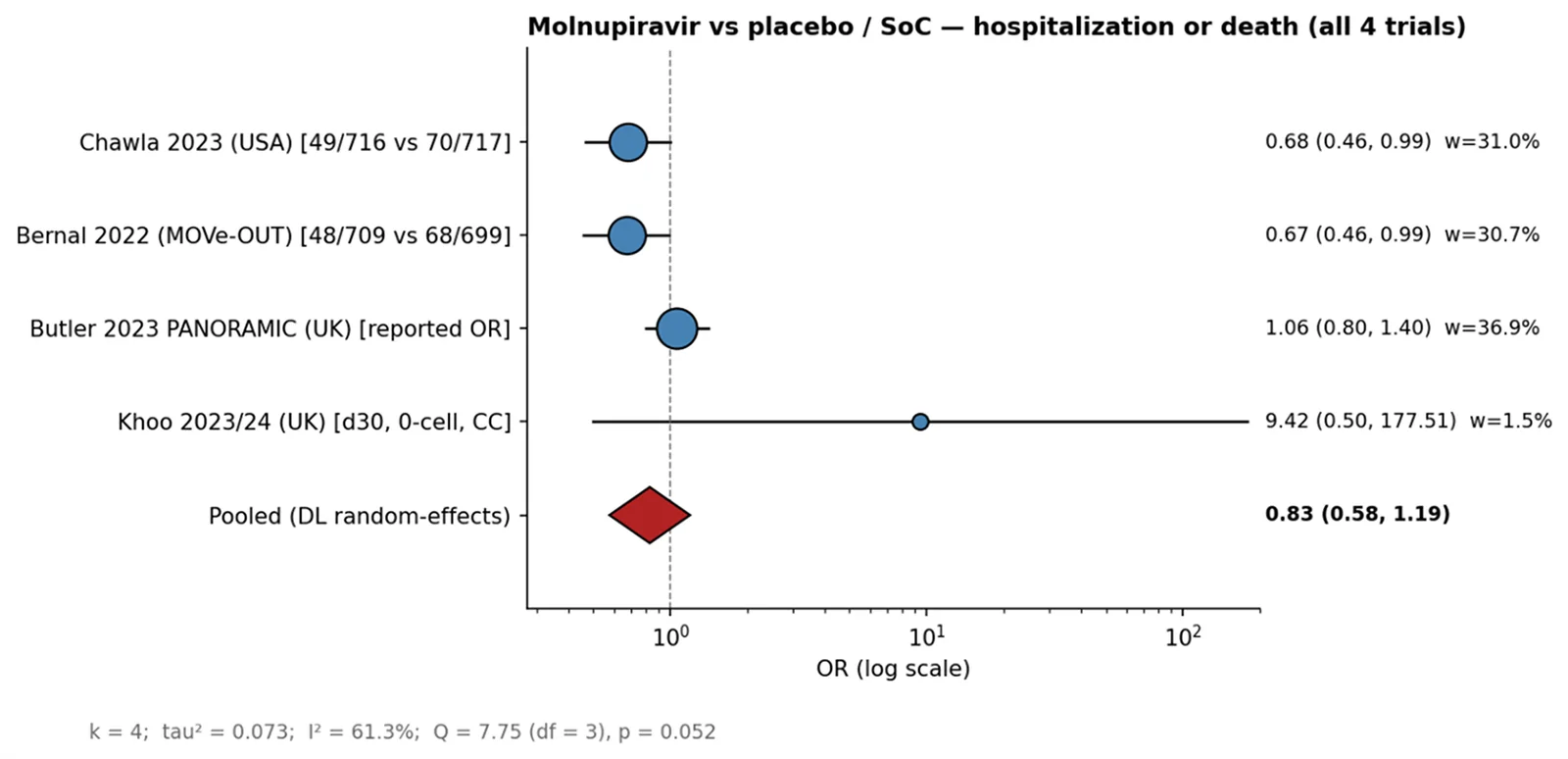
Molnupiravir versus placebo/usual care for hospitalization or death, all studies. Forest plot of trials comparing molnupiravir with placebo or usual care for the composite outcome of hospitalization or death. Forest plots show study-specific effect estimates and 95% confidence intervals for each comparison. Blue circles represent individual study estimates, with the size of the circle proportional to the study weight in the meta-analysis. Horizontal lines represent 95% confidence intervals. The red diamond represents the pooled random-effects estimate, and the width of the diamond represents the 95% confidence interval. The vertical dashed line at 1.0 represents the line of no effect. Estimates to the left of 1.0 favour the intervention for adverse clinical outcomes, while estimates to the right favour placebo, usual care, or standard of care. The x-axis is presented on a logarithmic scale. OR=odds ratio; RR=risk ratio; HR=hazard ratio; CI=confidence interval; DL=DerSimonian–Laird random-effects model; I²=percentage of total variability due to between-study heterogeneity; τ²=between-study variance; Q=Cochran’s heterogeneity statistic; df=degrees of freedom; p = p-value for the heterogeneity test; SoC=standard of care; UC=usual care; CC=continuity correction; MV=mechanical ventilation; IMV=invasive mechanical ventilation; NIV = non-invasive ventilation

**Fig. 7:**
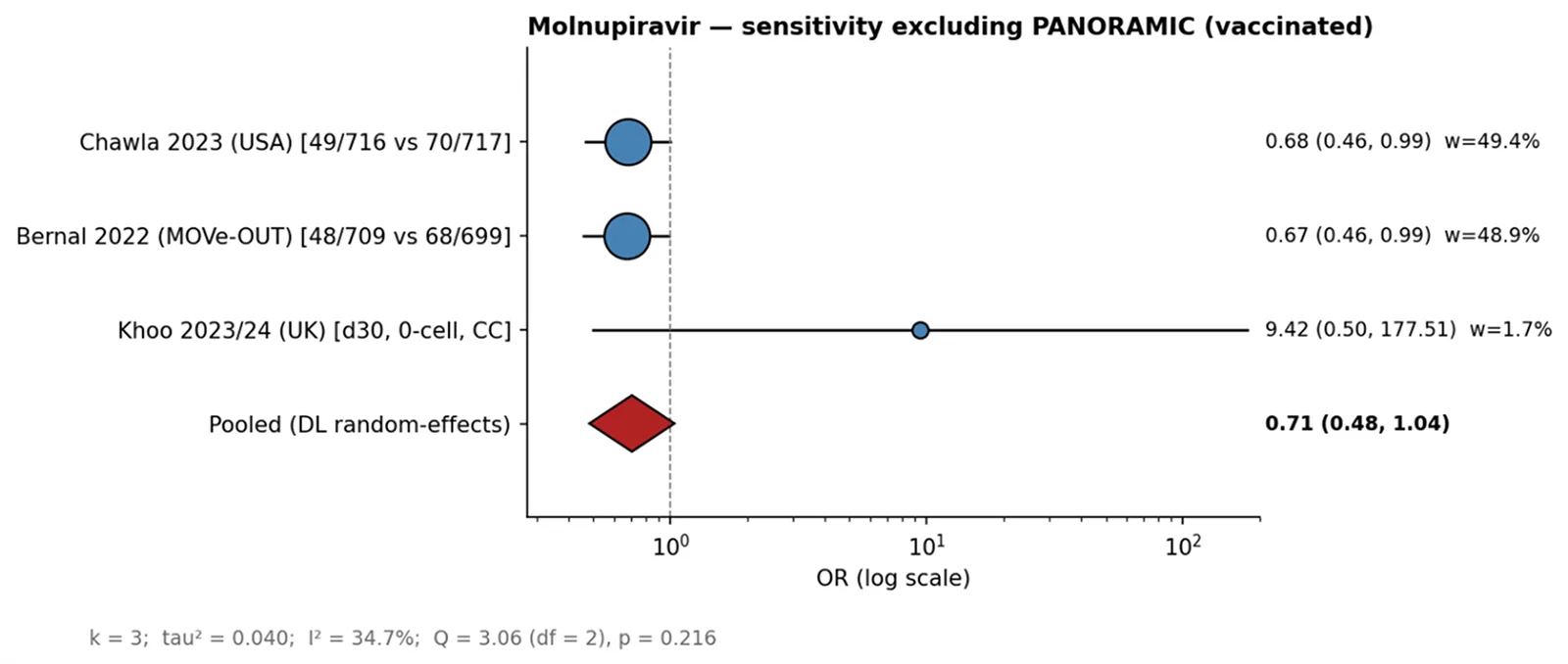
Molnupiravir versus placebo/usual care for hospitalization or death, excluding PANORAMIC. Sensitivity analysis excluding the PANORAMIC trial, comparing molnupiravir with placebo or usual care for hospitalization or death. Forest plots show study-specific effect estimates and 95% confidence intervals for each comparison. Blue circles represent individual study estimates, with the size of the circle proportional to the study weight in the meta-analysis. Horizontal lines represent 95% confidence intervals. The red diamond represents the pooled random-effects estimate, and the width of the diamond represents the 95% confidence interval. The vertical dashed line at 1.0 represents the line of no effect. Estimates to the left of 1.0 favour the intervention for adverse clinical outcomes, while estimates to the right favour placebo, usual care, or standard of care. The x-axis is presented on a logarithmic scale. OR=odds ratio; RR=risk ratio; HR=hazard ratio; CI=confidence interval; DL=DerSimonian–Laird random-effects model; I²=percentage of total variability due to between-study heterogeneity; τ²=between-study variance; Q=Cochran’s heterogeneity statistic; df=degrees of freedom; p = p-value for the heterogeneity test; SoC=standard of care; UC=usual care; CC=continuity correction; MV=mechanical ventilation; IMV=invasive mechanical ventilation; NIV = non-invasive ventilation

Across treatment-specific meta-analyses, most interventions did not show a clear reduction in hospitalization or hospitalization-containing outcomes. These findings are summarized in a combined treatment-specific forest plot, showing that pooled estimates for hydroxychloroquine (Fig. 3), ivermectin (Fig. 4), fluvoxamine (Fig. 5) crossed the null. Molnupiravir showed no clear benefit in the overall pooled analysis (OR 0.83, 95% CI 0.58–1.19; I²=61%; Fig. 6), but the estimate favoured treatment when restricted to unvaccinated high-risk outpatients (OR 0.68, 95% CI 0.52–0.89; I²=0%; Fig. 7), suggesting that pandemic period, baseline risk, and vaccination status are important effect modifiers.

### Intensive care unit admission or duration

We identified 24 outpatient randomized controlled trials reporting ICU admission or duration outcomes, including 20 repurposed treatment trials and four antiviral trials (Supplementary File 3, Table 4a and b). Across repurposed interventions, no consistent reductions in ICU admission or mechanical ventilation were observed. Trials of antibacterials such as azithromycin [31, 60] and doxycycline [32] showed null effects, as did colchicine [16] and hydroxychloroquine [20, 61, 62]. Ivermectin showed no benefit in either PRINCIPLE [40], TOGETHER [63], or smaller trials [64, 65], while fluvoxamine, alone or combined with bromhexine or cyproheptadine, showed reductions in severe respiratory failure requiring invasive mechanical ventilation in one recent study [66] but not in earlier trials [35, 48, 49]. Other repurposed agents (losartan [47], sulodexide [53], rivaroxaban [67], N-acetylcysteine/bromhexine [44]) also showed no statistically significant effects. Among antivirals, molnupiravir [45], lopinavir/ritonavir [13], sofosbuvir/daclatasvir and sofosbuvir/ravidasvir [68], and sofosbuvir alone [69] did not demonstrate any statistically significant differences in ICU admission rates compared with placebo or usual care.

**Fig. 8:**
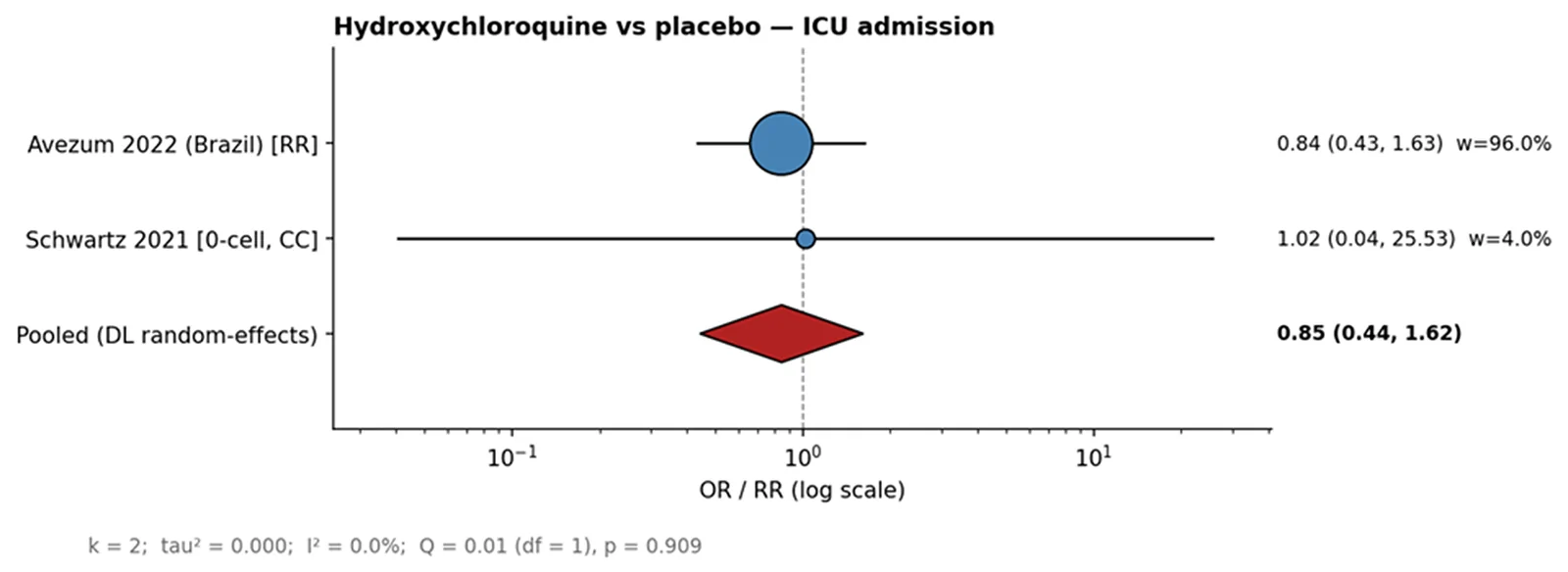
Hydroxychloroquine versus placebo for ICU admission. Forest plot of randomized trials comparing hydroxychloroquine with placebo for ICU admission. Forest plots show study-specific effect estimates and 95% confidence intervals for each comparison. Blue circles represent individual study estimates, with the size of the circle proportional to the study weight in the meta-analysis. Horizontal lines represent 95% confidence intervals. The red diamond represents the pooled random-effects estimate, and the width of the diamond represents the 95% confidence interval. The vertical dashed line at 1.0 represents the line of no effect. Estimates to the left of 1.0 favour the intervention for adverse clinical outcomes, while estimates to the right favour placebo, usual care, or standard of care. The x-axis is presented on a logarithmic scale. OR=odds ratio; RR=risk ratio; HR=hazard ratio; CI=confidence interval; DL=DerSimonian–Laird random-effects model; I²=percentage of total variability due to between-study heterogeneity; τ²=between-study variance; Q=Cochran’s heterogeneity statistic; df=degrees of freedom; p = p-value for the heterogeneity test; SoC=standard of care; UC=usual care; CC=continuity correction; MV=mechanical ventilation; IMV=invasive mechanical ventilation; NIV = non-invasive ventilation

**Fig. 9:**
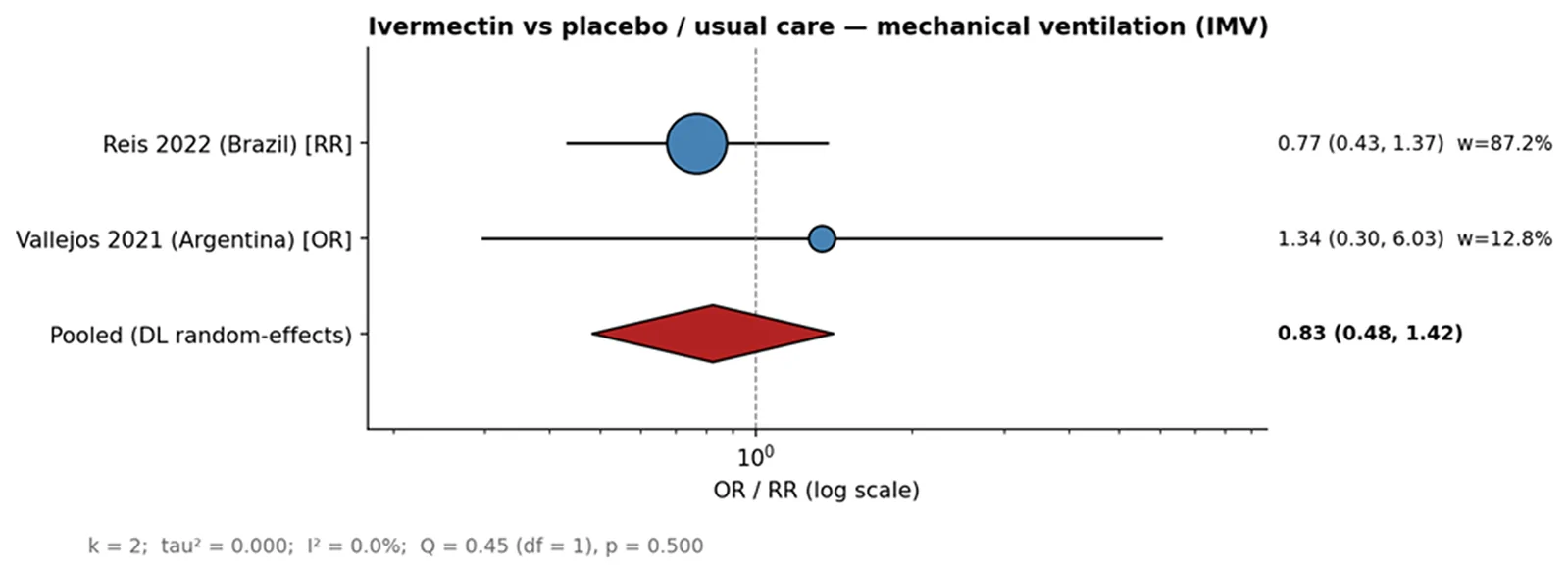
Ivermectin versus placebo/usual care for mechanical ventilation. Forest plot of randomized trials comparing ivermectin with placebo or usual care for invasive mechanical ventilation. Forest plots show study-specific effect estimates and 95% confidence intervals for each comparison. Blue circles represent individual study estimates, with the size of the circle proportional to the study weight in the meta-analysis. Horizontal lines represent 95% confidence intervals. The red diamond represents the pooled random-effects estimate, and the width of the diamond represents the 95% confidence interval. The vertical dashed line at 1.0 represents the line of no effect. Estimates to the left of 1.0 favour the intervention for adverse clinical outcomes, while estimates to the right favour placebo, usual care, or standard of care. The x-axis is presented on a logarithmic scale. OR=odds ratio; RR=risk ratio; HR=hazard ratio; CI=confidence interval; DL=DerSimonian–Laird random-effects model; I²=percentage of total variability due to between-study heterogeneity; τ²=between-study variance; Q=Cochran’s heterogeneity statistic; df=degrees of freedom; p = p-value for the heterogeneity test; SoC=standard of care; UC=usual care; CC=continuity correction; MV=mechanical ventilation; IMV=invasive mechanical ventilation; NIV = non-invasive ventilation

**Fig. 10:**
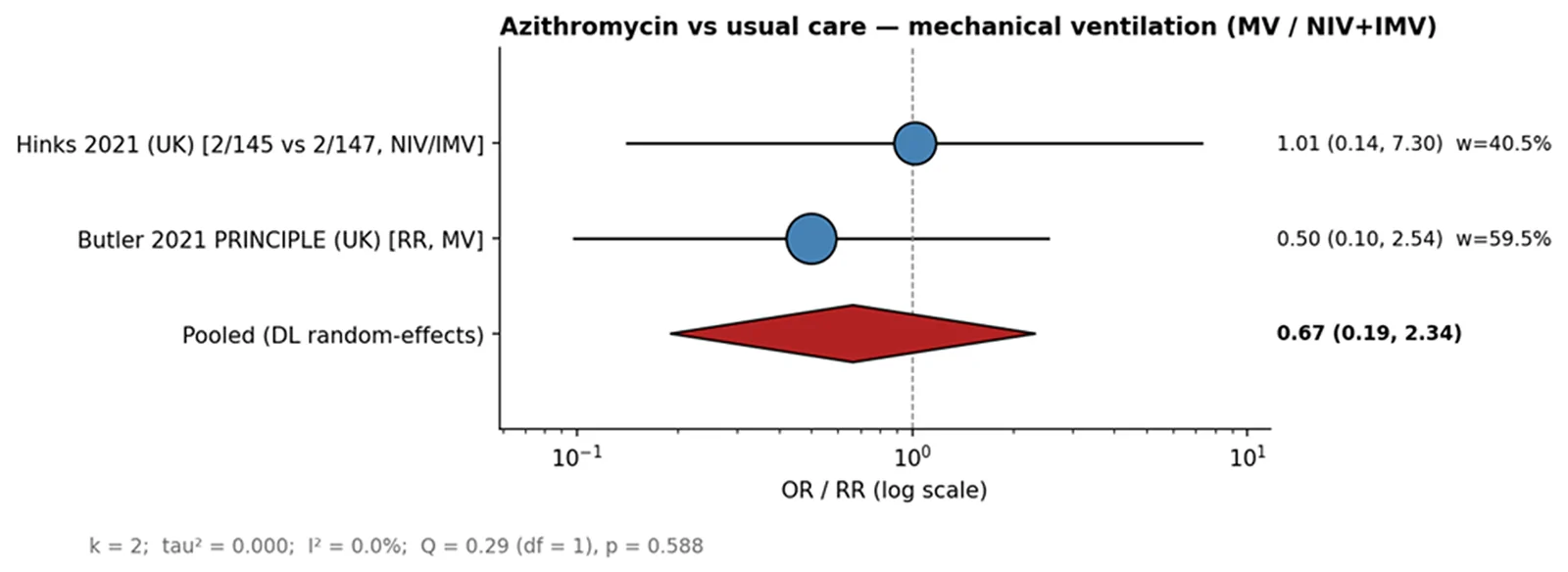
Azithromycin versus usual care for mechanical ventilation. Forest plot of randomized trials comparing azithromycin with usual care for mechanical ventilation. Forest plots show study-specific effect estimates and 95% confidence intervals for each comparison. Blue circles represent individual study estimates, with the size of the circle proportional to the study weight in the meta-analysis. Horizontal lines represent 95% confidence intervals. The red diamond represents the pooled random-effects estimate, and the width of the diamond represents the 95% confidence interval. The vertical dashed line at 1.0 represents the line of no effect. Estimates to the left of 1.0 favour the intervention for adverse clinical outcomes, while estimates to the right favour placebo, usual care, or standard of care. The x-axis is presented on a logarithmic scale. OR=odds ratio; RR=risk ratio; HR=hazard ratio; CI=confidence interval; DL=DerSimonian–Laird random-effects model; I²=percentage of total variability due to between-study heterogeneity; τ²=between-study variance; Q=Cochran’s heterogeneity statistic; df=degrees of freedom; p = p-value for the heterogeneity test; SoC=standard of care; UC=usual care; CC=continuity correction; MV=mechanical ventilation; IMV=invasive mechanical ventilation; NIV = non-invasive ventilation

**Fig. 11:**
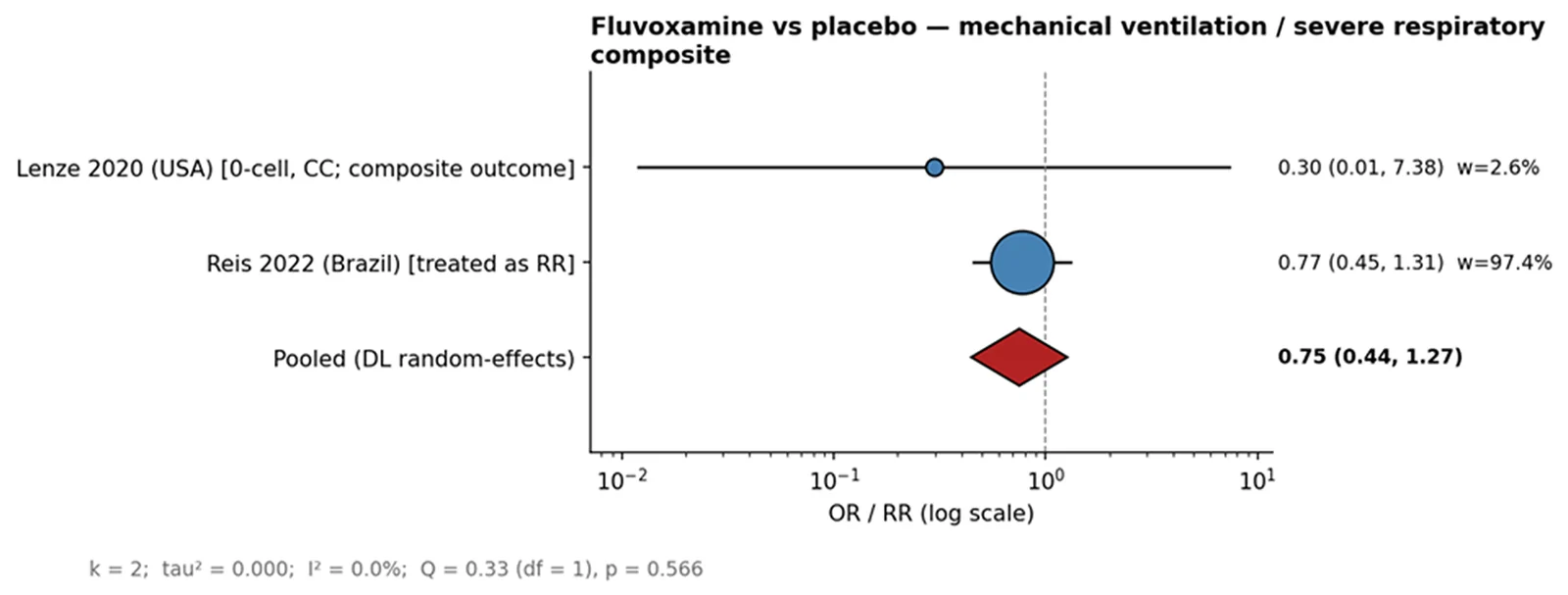
Fluvoxamine versus placebo for mechanical ventilation or severe respiratory outcomes. Forest plot of randomized trials comparing fluvoxamine with placebo for mechanical ventilation or severe respiratory composite outcomes. Forest plots show study-specific effect estimates and 95% confidence intervals for each comparison. Blue circles represent individual study estimates, with the size of the circle proportional to the study weight in the meta-analysis. Horizontal lines represent 95% confidence intervals. The red diamond represents the pooled random-effects estimate, and the width of the diamond represents the 95% confidence interval. The vertical dashed line at 1.0 represents the line of no effect. Estimates to the left of 1.0 favour the intervention for adverse clinical outcomes, while estimates to the right favour placebo, usual care, or standard of care. The x-axis is presented on a logarithmic scale. OR=odds ratio; RR=risk ratio; HR=hazard ratio; CI=confidence interval; DL=DerSimonian–Laird random-effects model; I²=percentage of total variability due to between-study heterogeneity; τ²=between-study variance; Q=Cochran’s heterogeneity statistic; df=degrees of freedom; p = p-value for the heterogeneity test; SoC=standard of care; UC=usual care; CC=continuity correction; MV=mechanical ventilation; IMV=invasive mechanical ventilation; NIV = non-invasive ventilation

Meta-analysis of ICU admission outcome was limited by sparse events and heterogeneous outcome definitions. Hydroxychloroquine versus placebo showed no clear reduction in ICU admission across two outpatient RCTs (pooled OR/RR 0.85, 95% CI 0.44–1.62; I²=0%; Fig. 8). Additional pooled analyses were available for mechanical ventilation or severe respiratory outcomes, including ivermectin (pooled OR/RR 0.83, 95% CI 0.48–1.42; I²=0%; Fig. 9), azithromycin (pooled OR/RR 0.67, 95% CI 0.19–2.34; I²=0%; Fig. 10), and fluvoxamine (pooled OR/RR 0.75, 95% CI 0.44–1.27; I²=0%; Fig. 11).

### Mortality

We identified 46 outpatient randomized trials that reported all-cause mortality as an outcome, including 36 repurposed treatment trials and 10 antiviral trials (Supplementary File 3, Table 5a and b).

Among repurposed agents, no consistent reduction in mortality was observed across therapeutic classes. Trials of colchicine [16, 17], azithromycin [60], hydroxychloroquine [20, 61, 62, 70], ivermectin [18, 39, 41, 63–65, 71], fluvoxamine [34, 49, 66, 72, 73], losartan [47], metformin [19], bromhexine-based regimens [44, 66] and rivaroxaban [67, 74, 75] showed no difference versus control. Small studies of amantadine [76], bosentan [77], camostat [21], ciclesonide [15], domperidone [78], raloxifene [79], and sulodexide [53] also reported no mortality events or non-significant differences. A modest reduction in mortality was reported in one trial of methylprednisolone [54], but this finding was not replicated elsewhere.

Among antivirals, most studies similarly found no meaningful reduction in mortality, including those testing favipiravir [80, 81], sofosbuvir-based regimens [68, 69], lopinavir/ritonavir [13], and bemnifosbuvir [9]. Trials of ensitrelvir [82], leritrelvir [10], and interferon-β (SNG001) [11] reported no deaths in either group. Only molnupiravir [56] demonstrated a statistically significant reduction in mortality through day 29, with an estimated 89% (95% CI 14–99) relative risk reduction compared with placebo.

**Fig. 12:**
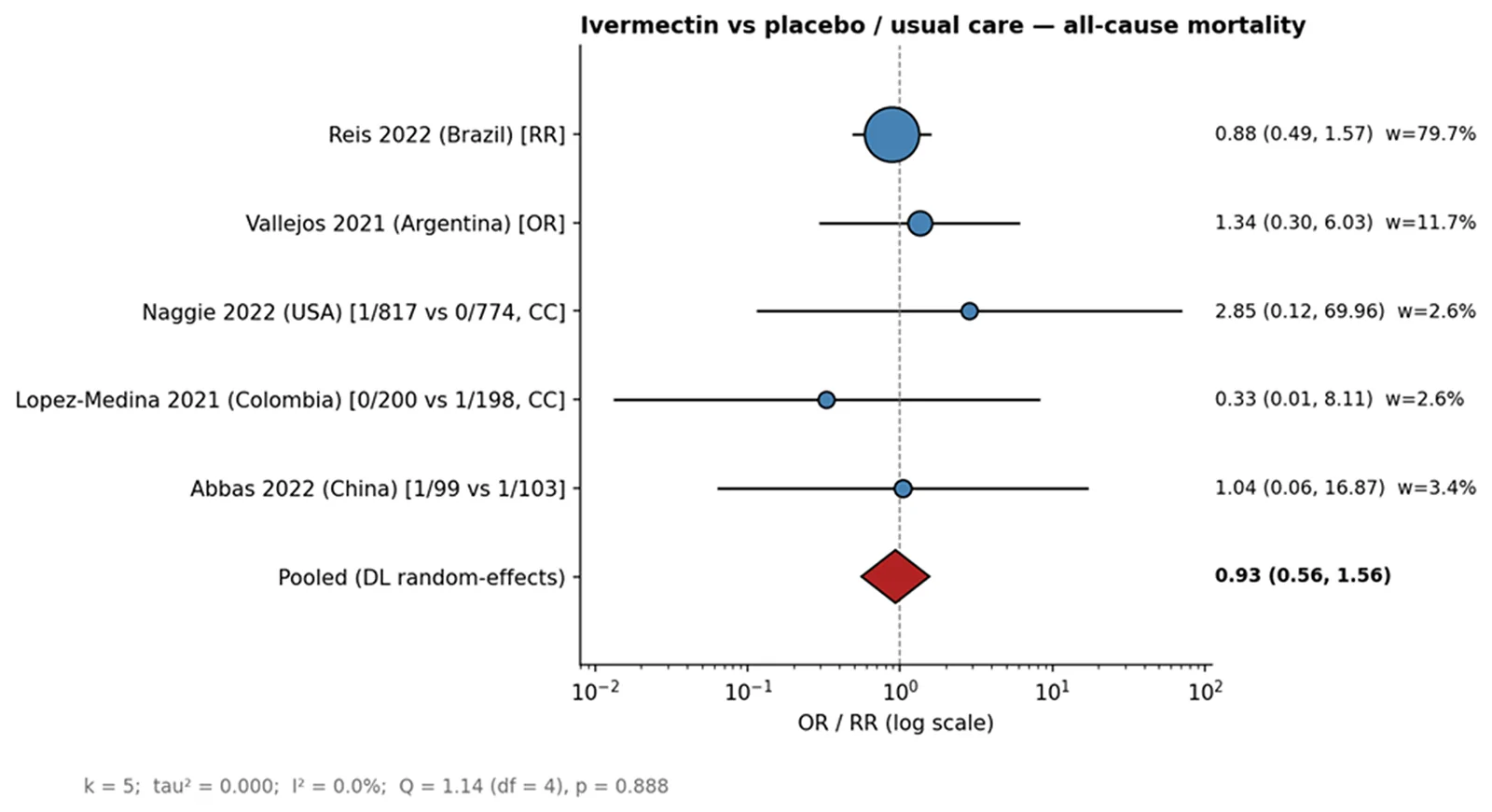
Ivermectin versus placebo/usual care for all-cause mortality. Forest plot of randomized trials comparing ivermectin with placebo or usual care for all-cause mortality. Forest plots show study-specific effect estimates and 95% confidence intervals for each comparison. Blue circles represent individual study estimates, with the size of the circle proportional to the study weight in the meta-analysis. Horizontal lines represent 95% confidence intervals. The red diamond represents the pooled random-effects estimate, and the width of the diamond represents the 95% confidence interval. The vertical dashed line at 1.0 represents the line of no effect. Estimates to the left of 1.0 favour the intervention for adverse clinical outcomes, while estimates to the right favour placebo, usual care, or standard of care. The x-axis is presented on a logarithmic scale. OR=odds ratio; RR=risk ratio; HR=hazard ratio; CI=confidence interval; DL=DerSimonian–Laird random-effects model; I²=percentage of total variability due to between-study heterogeneity; τ²=between-study variance; Q=Cochran’s heterogeneity statistic; df=degrees of freedom; p = p-value for the heterogeneity test; SoC=standard of care; UC=usual care; CC=continuity correction; MV=mechanical ventilation; IMV=invasive mechanical ventilation; NIV = non-invasive ventilation

**Fig. 13:**
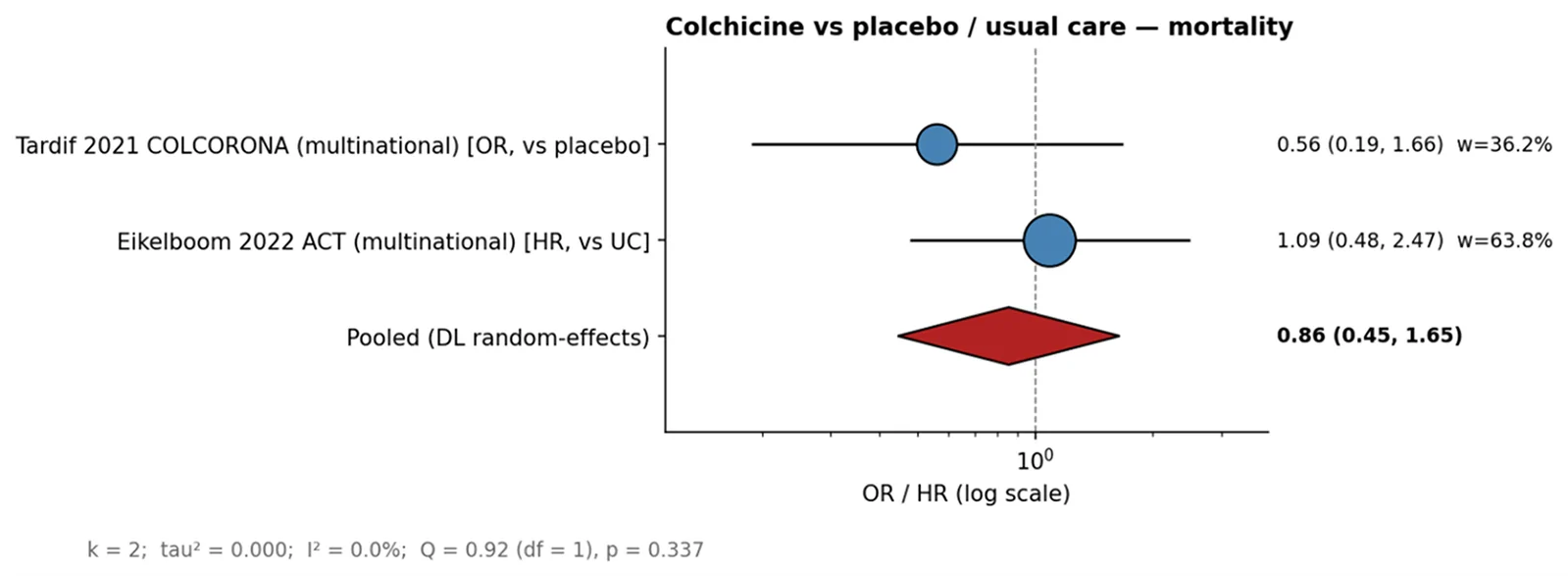
Colchicine versus placebo/usual care for all-cause mortality. Forest plot of randomized trials comparing colchicine with placebo or usual care for all-cause mortality. Forest plots show study-specific effect estimates and 95% confidence intervals for each comparison. Blue circles represent individual study estimates, with the size of the circle proportional to the study weight in the meta-analysis. Horizontal lines represent 95% confidence intervals. The red diamond represents the pooled random-effects estimate, and the width of the diamond represents the 95% confidence interval. The vertical dashed line at 1.0 represents the line of no effect. Estimates to the left of 1.0 favour the intervention for adverse clinical outcomes, while estimates to the right favour placebo, usual care, or standard of care. The x-axis is presented on a logarithmic scale. OR=odds ratio; RR=risk ratio; HR=hazard ratio; CI=confidence interval; DL=DerSimonian–Laird random-effects model; I²=percentage of total variability due to between-study heterogeneity; τ²=between-study variance; Q=Cochran’s heterogeneity statistic; df=degrees of freedom; p = p-value for the heterogeneity test; SoC=standard of care; UC=usual care; CC=continuity correction; MV=mechanical ventilation; IMV=invasive mechanical ventilation; NIV = non-invasive ventilation

**Fig. 14:**
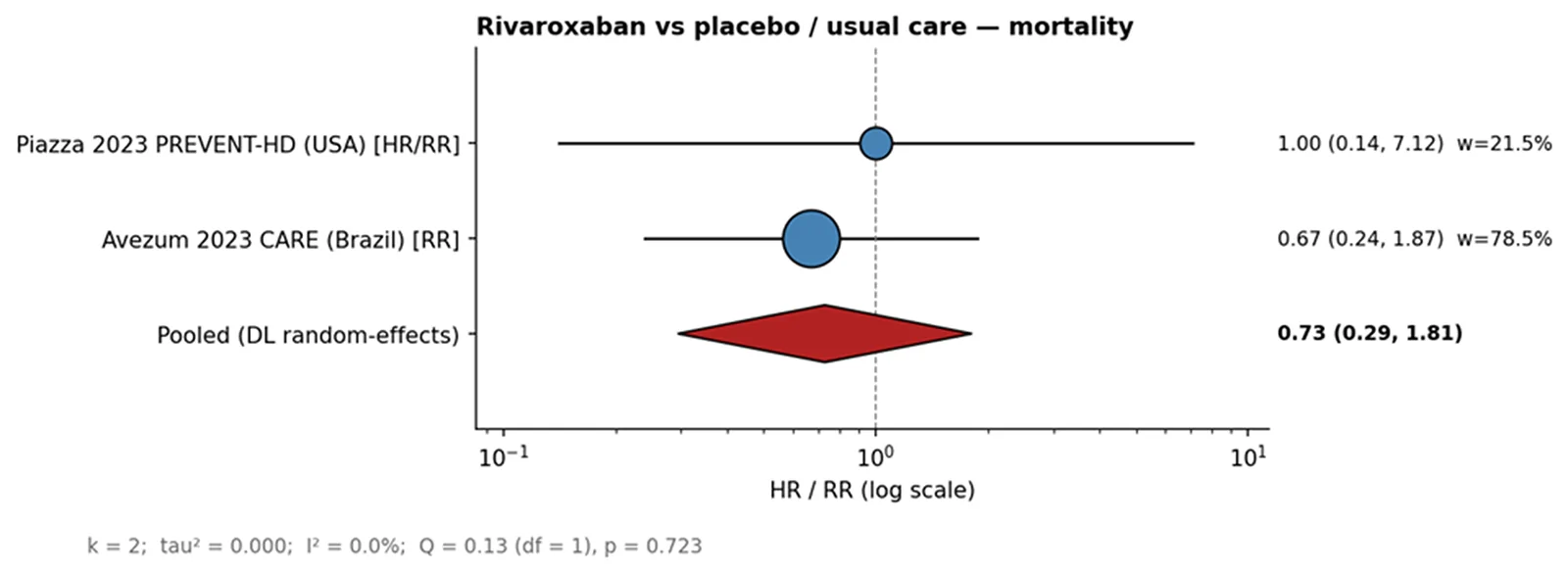
Rivaroxaban versus placebo/usual care for all-cause mortality. Forest plot of randomized trials comparing rivaroxaban with placebo or usual care for all-cause mortality. Forest plots show study-specific effect estimates and 95% confidence intervals for each comparison. Blue circles represent individual study estimates, with the size of the circle proportional to the study weight in the meta-analysis. Horizontal lines represent 95% confidence intervals. The red diamond represents the pooled random-effects estimate, and the width of the diamond represents the 95% confidence interval. The vertical dashed line at 1.0 represents the line of no effect. Estimates to the left of 1.0 favour the intervention for adverse clinical outcomes, while estimates to the right favour placebo, usual care, or standard of care. The x-axis is presented on a logarithmic scale. OR=odds ratio; RR=risk ratio; HR=hazard ratio; CI=confidence interval; DL=DerSimonian–Laird random-effects model; I²=percentage of total variability due to between-study heterogeneity; τ²=between-study variance; Q=Cochran’s heterogeneity statistic; df=degrees of freedom; p = p-value for the heterogeneity test; SoC=standard of care; UC=usual care; CC=continuity correction; MV=mechanical ventilation; IMV=invasive mechanical ventilation; NIV = non-invasive ventilation

Mortality was rare across trials, and many comparisons reported zero deaths in both arms; therefore, only ivermectin, colchicine, and rivaroxaban had sufficient informative data for pooling. Ivermectin showed no statistically significant effect on mortality across five trials (pooled relative effect 0.93, 95% CI 0.56–1.56; I² = 0%), with the estimate largely driven by the TOGETHER trial (Fig. 12). Similarly, colchicine showed an imprecise effect across two trials (0.86, 95% CI 0.45–1.65; I² = 0%) (Fig. 13), and rivaroxaban showed an imprecise effect across two trials (0.73, 95% CI 0.29–1.81; I² = 0%) (Fig. 14).

### Safety and adverse events

Across the included outpatient trials, adverse events were generally similar between active treatment and control groups, or modestly higher with active treatment, with events largely consistent with each drug’s known safety profile. Hydroxychloroquine was associated with more mild gastrointestinal events [61], but no outpatient trial detected an excess of QT prolongation, ventricular arrhythmia, retinopathy, or sudden cardiac death. Colchicine increased gastrointestinal events [33], while fluvoxamine was associated with modest increases in nausea, dyspepsia, and insomnia [34, 35]; ivermectin, molnupiravir, inhaled budesonide, and metformin were generally well tolerated across trials.

For serious adverse events, pooled estimates for colchicine, ivermectin, fluvoxamine, and molnupiravir did not show a statistically significant excess compared with placebo or usual care. These estimates should be interpreted cautiously because event counts were sparse for several drugs, heterogeneity was present for colchicine and molnupiravir, and SAE ascertainment differed across trials, particularly between MOVe-OUT [56] and PANORAMIC [57]. Importantly, in placebo-controlled outpatient COVID-19 trials, COVID-19-related hospitalization was often the dominant SAE and was also a key efficacy outcome. Therefore, pooled SAE estimates should not be interpreted as independent measures of drug-attributable harm, but rather as a combined reflection of severe COVID-19 events and the absence of clear new drug-specific safety signals.

## Discussion

Across 124 outpatient randomized trials evaluating 137 therapeutic interventions, we found no consistent evidence of benefit for most repurposed or antiviral agents in improving clinically meaningful outcomes in mild to moderate cases of SARS-CoV-2. Repurposed therapies such as fluvoxamine, ivermectin, hydroxychloroquine, colchicine, and anticoagulants showed largely null or inconsistent effects across outcomes. Inhaled budesonide demonstrated faster recovery, and a small number of studies suggested potential benefit for metformin, sulodexide, and methylprednisolone, though none were consistently replicated. Among antivirals, molnupiravir showed benefits for selected outcomes, including shorter time to recovery in several RCTs [4–6] and reduced emergency department or acute care visits in one trial [45]; however, it did not consistently reduce hospitalization or mortality. Other antivirals, including favipiravir, interferon-β, and lopinavir/ritonavir, showed no clear benefit across major clinical outcomes.

Our findings are broadly consistent with previous systematic reviews but provide a more comprehensive and updated synthesis through 2024 [22–24, 83]. These reviews generally concluded that evidence for most repurposed therapies was uncertain, low quality, or did not support routine use in non-hospitalized patients [22, 83]. Our review reinforces this conclusion across a larger and more recent body of outpatient randomized evidence. In particular, we found no consistent improvement in hospitalization, ICU admission, mortality, or recovery outcomes for several widely studied repurposed agents, including hydroxychloroquine, ivermectin, colchicine, fluvoxamine, and anticoagulants.

There were also important differences between our review and earlier syntheses. Previous living reviews were essential during the acute phase of the pandemic, when rapid evidence updates were needed to inform clinical guidance [23, 83]. However, many of those conclusions were based on smaller numbers of trials, early pandemic populations, and evidence generated before widespread vaccination and later SARS-CoV-2 variants. The review by Sullivan et al. included 61 outpatient randomized trials and found limited evidence of benefit for most small-molecule antivirals and repurposed agents, with clearer effects observed for monoclonal antibodies [23, 24, 83]. In contrast, our review includes 124 outpatient randomized trials evaluating 137 therapeutic interventions and focuses specifically on oral and inhaled pharmaceutical treatments. This broader scope allowed us to examine whether signals seen in early or smaller studies were replicated in larger or more pragmatic trials.

The changing immune context of the pandemic is important when interpreting these findings. Trials conducted in 2020 and early 2021 largely enrolled participants with little or no vaccine-derived immunity and limited prior exposure to SARS-CoV-2. In those settings, baseline risk of hospitalization and death was higher, and treatment effects on severe outcomes were more likely to be detected. Trials conducted later in the pandemic often enrolled populations with higher vaccination coverage, greater prior infection, different circulating variants, and lower baseline risk of severe outcomes. These changes may reduce the absolute benefit of treatment, particularly for outcomes such as hospitalization, ICU admission, and mortality. They also constrain quantitative synthesis: ICU admission and mechanical ventilation were rare across our included trials, yielding only four defensibly pooled drug-specific estimates (hydroxychloroquine for ICU admission, and ivermectin, azithromycin, and fluvoxamine for mechanical ventilation or severe respiratory composite), all consistent with no effect. Mortality was even rarer, with most trials reporting zero deaths in both arms; only three drug-specific pools (ivermectin, colchicine, rivaroxaban) had sufficient informative events for pooling, and none reached statistical significance.

Our findings suggest that most early signals for repurposed therapies did not translate into consistent clinical benefit when tested across broader outpatient populations. For example, early interest in hydroxychloroquine, ivermectin, and fluvoxamine was not supported by consistent reductions in hospitalization, ICU admission, or mortality across subsequent trials. Inhaled budesonide showed more favorable effects on time to recovery in selected trials [14, 37] but this did not translate into consistent evidence of reduced severe outcomes. Similarly, metformin showed promising findings in some analyses, including signals related to emergency department visits or longer-term outcomes, but the evidence remains insufficient to support a broad conclusion of effectiveness across the acute clinical outcomes assessed in this review.

Among antivirals, our findings are consistent with earlier reviews [22–24, 83], showing that the effect of these treatments depends on the outcome and the population studied. For example, Molnupiravir shortened time to recovery in several trials, but its effect on hospitalization and mortality was not consistent. The difference between MOVe-OUT and PANORAMIC is important here. MOVe-OUT [56] showed that molnupiravir reduced hospitalization or death among unvaccinated outpatients with mild-to-moderate COVID-19 who were at higher risk of severe disease. In contrast, PANORAMIC [57] was conducted later in the pandemic, in a much larger and largely vaccinated outpatient population, and did not show a reduction in hospitalization or death, although it did shorten time to recovery. In our pooled analyses, restricting to unvaccinated, higher-risk outpatients preserved the effect on hospitalization (OR 0.68, 95% CI 0.52–0.89; I²=0%), whereas the overall pool that included PANORAMIC was non-significant with substantial heterogeneity (OR 0.83, 95% CI 0.58–1.19; I²=61%). This difference likely reflects the lower baseline risk in PANORAMIC, vaccination status, changes in circulating variants, and changes in usual care over time. These findings suggest that molnupiravir is most likely to benefit patients at higher risk of progression, particularly those who are unvaccinated. Similarly, other oral or inhaled antivirals, including favipiravir, interferon-β, and lopinavir/ritonavir, did not show consistent benefit across major clinical outcomes. These findings underscore the importance of interpreting outpatient therapeutic effects in relation to population risk, vaccination status, timing of treatment initiation, variant era, and baseline event rates.

When findings were interpreted in relation to risk of bias, the overall conclusions were unchanged. Most treatments with null or inconsistent effects, including hydroxychloroquine, ivermectin, fluvoxamine, and colchicine, did not show consistent benefit when greater weight was placed on trials rated as low risk of bias.

The strengths of this systematic review include its broad scope, inclusion of all major therapeutic classes, and up-to-date coverage through 2024. However, several limitations should be noted. Many trials were open-label, introducing potential bias, particularly for subjective outcomes such as time to recovery. Although meta-analyses were conducted for selected binary outcomes where data were sufficiently comparable, considerable heterogeneity in dosing regimens, duration, timing of treatment, population risk, vaccination status, and outcome definitions limited quantitative synthesis across several outcomes and treatment classes. Finally, the inclusion of studies conducted over a four-year period means evolving immunity, viral variants, and standard care may have influenced treatment effects in ways not fully captured by available data.

## Conclusion

Across 124 outpatient randomized trials, most repurposed therapies showed no consistent benefit for recovery, hospitalization, ICU admission, or mortality. A few agents, such as inhaled budesonide, metformin, and sulodexide, showed limited or setting-specific effects. Molnupiravir showed evidence of benefit for time to recovery, but effects on hospitalization and mortality were not consistent across trials. The findings highlight the limited effectiveness of most early repurposed drugs and the need for ongoing investigation as new antivirals and variants emerge. Regular evidence synthesis and standardized outcome reporting will remain critical for guiding outpatient treatment strategies for SARS-CoV-2.

## Supplementary Information

Below is the link to the electronic supplementary material.

Supplementary Material 1

Supplementary Material 2

Supplementary Material 3

## Data Availability

The data underlying this study are available upon reasonable request from the corresponding author (ADP).

## Abbreviations

SARS-CoV-2: Severe Acute Respiratory Syndrome Coronavirus 2
ICU: Intensive Care Unit
ED: Emergency Department
WHO: World Health Organization
RCT: Randomized Controlled Trial
CI: Confidence Interval
