# Supplementary material for: Effectiveness of oral and inhaled repurposed and antiviral pharmaceutical treatments for acute SARS-CoV-2 in outpatient settings: a systematic review of 124 randomized trials
Source: BMC Infect Dis. 2026 Jul 23;26:1829. doi: 10.1186/s12879-026-13901-7 (PMC13682097; doi:10.1186/s12879-026-13901-7)
Supplement: Supplementary file 1 [file 12879_2026_13901_MOESM1_ESM.docx]

**Supplementary File 1. Complete Search Strategy**

Ovid Multifile

Database: Embase Classic+Embase <1947 to 2023 September 01>, International Pharmaceutical Abstracts <1970 to August 2023>, Ovid MEDLINE(R) ALL <1946 to September 01, 2023>

Search Strategy:

--------------------------------------------------------------------------------

1	COVID-19/ (369885)

2	(COVID-19 or COVID19).tw,kw,kf. (711843)

3	((coronavirus* or corona virus*) and (hubei or wuhan or beijing or shanghai)).tw,kw,kf. (14739)

4	(wuhan adj5 virus*).tw,kw,kf. (914)

5	(2019-nCoV or 19nCoV or 2019nCoV).tw,kw,kf. (4578)

6	(SARS-CoV-2 or SARS-CoV2 or SARSCoV-2 or SARSCoV2 or SARS2 or SARS-2 or severe acute respiratory syndrome coronavirus 2).tw,kw,kf. (280185)

7	(2019-novel CoV or Sars-coronavirus2 or Sars-coronavirus-2 or SARS-like coronavirus* or ((novel or new or nouveau) adj2 (CoV or nCoV or covid or coronavirus* or corona virus or Pandemi*2))).tw,kw,kf. (48067)

8	(novel coronavirus* or novel corona virus* or novel CoV).tw,kw,kf. (28328)

9	((coronavirus* or corona virus*) adj ("19" or "2019")).tw,kw,kf. (7994)

10	("coronavirus 2" or "corona virus 2").tw,kw,kf. (74681)

11	(coronavirus* or corona virus*).ti,kw,kf. (115930)

12	COVID.ti,kw,kf. (606424)

13	("B.1.1.7" or "B.1.351" or "B.1.617" or "B.1.427" or "B.1.429").tw,kw,kf,rx,px,ox. (3876)

14	("B.1.1.529" or "BA.1" or "BA.2" or "BA.2.86" or "BA.3" or "BA.4" or "BA.5" or "BQ.1" or "BQ.1.1" or XBB or "XBB.1.5" or "XBB.1.6").tw,kw,kf,rx,px,ox. (15914)

15	("P.1" and (Brazil* or variant?)).tw,kw,kf,rx,px,ox. (4910)

16	(((alpha or beta or delta or eta or gamma or iota or kappa or lambda or omicron or zeta) adj3 variant?) and (coronavirus* or corona virus* or covid*)).tw,kw,kf. (14307)

17	or/1-16 [COVID-19] (840318)

18	(exp Child/ or exp Infant/) not (Adolescent/ or exp Adult/) (3579912)

19	17 not 18 [18 AND UNDER POPULATION REMOVED] (812684)

20	COVID-19 Drug Treatment/ [COVID-19 DRUG TREATMENT PT 1] (12174)

21	Drug Therapy/ (987728)

22	(drug or drugs or medication? or medicines or pharmaceutic*).ti,kw,kf. (1912261)

23	(pharmacotherap* or pharmaco-therap*).ti,kw,kf. (37378)

24	(treat* or therap*).ti,kw,kf. (6617095)

25	(paxlovid$2 or (nirmatrelvir* adj2 ritonavir*)).tw,kw,kf. (1734)

26	(("pf 07321332" or pf07321332 or pf 7321332 or pf7321332) adj2 ritonavir*).tw,kw,kf. (22)

27	(remdesivir* or "gs 5734" or gs5734 or "gs 465124" or "gs 829143" or "ro 7286260" or ro7286260 or redyx$2 or veklury$2 or UNII-3QKI37EEHE or 1809249-37-3).tw,kw,kf,rn. (16523)

28	(molnupiravir* or "eidd 2801" or eidd2801 or "mk 4482" or mk4482 or lagevrio$2 or UNII-YA84KI1VEW or 2349386-89-4 or 2492423-29-5).tw,kw,kf,rn. (1985)

29	or/21-28 [COVID DRUG TREATMENTS] (8856354)

30	19 and 29 [COVID-19 DRUG TREATMENT PT 2] (101664)

31	20 or 30 [COVID-19 DRUG TREATMENTS] (107260)

32	exp Animals/ not Humans/ (17620244)

33	31 not 32 [ANIMAL-ONLY REMOVED] (106007)

34	(address or autobiography or bibliography or biography or comment or dictionary or directory or editorial or "expression of concern" or festschrift or historical article or interactive tutorial or lecture or legal case or legislation or news or newspaper article or patient education handout or personal narrative or portrait or preprint or video-audio media or webcast).pt. (3023739)

35	Case Reports.pt. (2354415)

36	Single-Case Studies as Topic/ (507)

37	(case study or case studies or case report? or case series).ti,kw,kf. (1016485)

38	chart review?.ti,kw,kf. (5240)

39	33 not (34 or 35 or 36 or 37 or 38) [OPINION PIECES, CASE STUDIES, CHART REVIEWS REMOVED] (94294)

40	(controlled clinical trial or randomized controlled trial or pragmatic clinical trial or equivalence trial).pt. (691162)

41	"Clinical Trials as Topic"/ (298710)

42	(randomi#ed or randomi#ation? or randomly or RCT or placebo*).tw,kw,kf. (3046839)

43	((singl* or doubl* or trebl* or tripl*) adj (mask* or blind* or dumm*)).tw,kw,kf. (511389)

44	trial.ti. (718162)

45	or/40-44 (3680859)

46	39 and 45 [RCTs] (10431)

47	limit 46 to yr="2019-current" (10391)

48	47 use medall [MEDLINE RECORDS] (4043)

49	coronavirus disease 2019/ (580164)

50	(COVID-19 or COVID19).tw,kw,kf. (711843)

51	((coronavirus* or corona virus*) and (hubei or wuhan or beijing or shanghai)).tw,kw,kf. (14739)

52	(wuhan adj5 virus*).tw,kw,kf. (914)

53	(2019-nCoV or 19nCoV or 2019nCoV).tw,kw,kf. (4578)

54	(SARS-CoV-2 or SARS-CoV2 or SARSCoV-2 or SARSCoV2 or SARS2 or SARS-2 or severe acute respiratory syndrome coronavirus 2).tw,kw,kf. (280185)

55	(2019-novel CoV or Sars-coronavirus2 or Sars-coronavirus-2 or SARS-like coronavirus* or ((novel or new or nouveau) adj2 (CoV or nCoV or covid or coronavirus* or corona virus or Pandemi*2))).tw,kw,kf. (48067)

56	(novel coronavirus* or novel corona virus* or novel CoV).tw,kw,kf. (28328)

57	((coronavirus* or corona virus*) adj ("19" or "2019")).tw,kw,kf. (7994)

58	("coronavirus 2" or "corona virus 2").tw,kw,kf. (74681)

59	(coronavirus* or corona virus*).ti,kw,kf. (115930)

60	COVID.ti,kw,kf. (606424)

61	("B.1.1.7" or "B.1.351" or "B.1.617" or "B.1.427" or "B.1.429").tw,kw,kf,rn. (3822)

62	("B.1.1.529" or "BA.1" or "BA.2" or "BA.2.86" or "BA.3" or "BA.4" or "BA.5" or "BQ.1" or "BQ.1.1" or XBB or "XBB.1.5" or "XBB.1.6").tw,kw,kf,rn. (15256)

63	("P.1" and (Brazil* or variant?)).tw,kw,kf,rn. (4865)

64	(((alpha or beta or delta or eta or gamma or iota or kappa or lambda or omicron or zeta) adj3 variant?) and (coronavirus* or corona virus* or covid*)).tw,kw,kf. (14307)

65	or/49-64 [COVID-19] (860900)

66	(exp child/ or exp adolescent/) not exp adult/ (4482400)

67	65 not 66 [18 AND UNDER POPULATION REMOVED] (820946)

68	coronavirus disease 2019/dt [Drug Therapy] (47205)

69	COVID-19 pharmacotherapy/ (86)

70	68 or 69 [COVID-19 DRUG TREATMENT PT 1] (47280)

71	drug therapy/ (987728)

72	antiviral therapy/ (41958)

73	anti-SARS-CoV-2 agent/ or anticoronavirus agent/ (1965)

74	(drug or drugs or medication? or medicines or pharmaceutic*).ti,kw,kf. (1912261)

75	(pharmacotherap* or pharmaco-therap*).ti,kw,kf. (37378)

76	(treat* or therap*).ti,kw,kf. (6617095)

77	nirmatrelvir plus ritonavir/ (1077)

78	(paxlovid$2 or (nirmatrelvir* adj2 ritonavir*)).tw,kw,kf. (1734)

79	(("pf 07321332" or pf07321332 or pf 7321332 or pf7321332) adj2 ritonavir*).tw,kw,kf. (22)

80	remdesivir/ (12248)

81	(remdesivir* or "gs 5734" or gs5734 or "gs 465124" or "gs 829143" or "ro 7286260" or ro7286260 or redyx$2 or veklury$2 or UNII-3QKI37EEHE or 1809249-37-3).tw,kw,kf,rn. (16523)

82	molnupiravir/ (1256)

83	(molnupiravir* or "eidd 2801" or eidd2801 or "mk 4482" or mk4482 or lagevrio$2 or UNII-YA84KI1VEW or 2349386-89-4 or 2492423-29-5).tw,kw,kf,rn. (1985)

84	or/71-83 [COVID DRUG TREATMENTS] (8876454)

85	67 and 84 [COVID-19 DRUG TREATMENT PT 2] (105615)

86	70 or 85 [COVID-19 DRUG TREATMENTS] (136762)

87	(exp animal/ or exp animal experimentation/ or exp animal model/ or exp animal experiment/ or nonhuman/ or exp vertebrate/) not (exp human/ or exp human experimentation/ or exp human experiment/) (13201263)

88	86 not 87 [ANIMAL-ONLY REMOVED] (133128)

89	(conference abstract or editorial or preprint).pt. (6418649)

90	letter.pt. not (letter.pt. and randomized controlled trial/) (2502540)

91	case report/ (3011915)

92	exp case study/ (2460027)

93	(case study or case studies or case report? or case series).ti,kw,kf. (1016485)

94	medical record review/ (169424)

95	chart review?.ti,kw,kf. (5240)

96	88 not (89 or 90 or 91 or 92 or 93 or 94 or 95) [OPINION PIECES, CASE STUDIES, CHART REVIEWS REMOVED] (87478)

97	exp randomized controlled trial/ or controlled clinical trial/ (1670287)

98	clinical trial/ (1628908)

99	exp "controlled clinical trial (topic)"/ (270063)

100	(randomi#ed or randomi#ation? or randomly or RCT or placebo*).tw,kw,kf. (3046839)

101	((singl* or doubl* or trebl* or tripl*) adj (mask* or blind* or dumm*)).tw,kw,kf. (511389)

102	trial.ti. (718162)

103	or/97-102 (4571085)

104	96 and 103 [RCTs] (10172)

105	limit 104 to yr="2019-current" (10100)

106	105 use emczd [EMBASE RECORDS] (6995)

107	covid 19.sh. (237626)

108	(COVID-19 or COVID19).ti,ab. (684094)

109	((coronavirus* or corona virus*) and (hubei or wuhan or beijing or shanghai)).ti,ab. (14154)

110	(wuhan adj5 virus*).ti,ab. (883)

111	(2019-nCoV or 19nCoV or 2019nCoV).ti,ab. (3491)

112	(SARS-CoV-2 or SARS-CoV2 or SARSCoV-2 or SARSCoV2 or SARS2 or SARS-2 or severe acute respiratory syndrome coronavirus 2).ti,ab. (248598)

113	(2019-novel CoV or Sars-coronavirus2 or Sars-coronavirus-2 or SARS-like coronavirus* or ((novel or new or nouveau) adj2 (CoV or nCoV or covid or coronavirus* or corona virus or Pandemi*2))).ti,ab. (45978)

114	(novel coronavirus* or novel corona virus* or novel CoV).ti,ab. (26427)

115	((coronavirus* or corona virus*) adj ("19" or "2019")).ti,ab. (7381)

116	("coronavirus 2" or "corona virus 2").ti,ab. (70774)

117	(coronavirus* or corona virus*).ti,ab. (261945)

118	COVID.ti. (523012)

119	("B.1.1.7" or "B.1.351" or "B.1.617" or "B.1.427" or "B.1.429").ti,ab. (3746)

120	("B.1.1.529" or "BA.1" or "BA.2" or "BA.2.86" or "BA.3" or "BA.4" or "BA.5" or "BQ.1" or "BQ.1.1" or XBB or "XBB.1.5" or "XBB.1.6").ti,ab. (15060)

121	("P.1" and (Brazil* or variant?)).ti,ab. (4806)

122	(((alpha or beta or delta or eta or gamma or iota or kappa or lambda or omicron or zeta) adj3 variant?) and (coronavirus* or corona virus* or covid*)).ti,ab. (12962)

123	or/107-122 [COVID-19] (833213)

124	(drug or drugs or medication? or medicines or pharmaceutic*).ti. (1486162)

125	(pharmacotherap* or pharmaco-therap*).ti. (28913)

126	(treat* or therap*).ti. (5957840)

127	("Nirmatrelvir combination, ritonavir" or "Ritonavir combination, nirmatrelvir" or "Antivirals nirmatrelvir, combination, ritonavir").sh. (34)

128	(paxlovid$2 or (nirmatrelvir* adj2 ritonavir*)).ti,ab,tn. (1640)

129	(("pf 07321332" or pf07321332 or pf 7321332 or pf7321332) adj2 ritonavir*).ti,ab. (18)

130	Remdesivir$.sh. (12250)

131	(remdesivir* or "gs 5734" or gs5734 or "gs 465124" or "gs 829143" or "ro 7286260" or ro7286260 or redyx$2 or veklury$2 or UNII-3QKI37EEHE or 1809249-37-3).ti,ab,rn,tn. (16332)

132	molnupiravir$.sh. (1257)

133	(molnupiravir* or "eidd 2801" or eidd2801 or "mk 4482" or mk4482 or lagevrio$2 or UNII-YA84KI1VEW or 2349386-89-4 or 2492423-29-5).ti,ab,rn,tn. (1921)

134	or/124-133 [COVID DRUG TREATMENTS] (7281715)

135	123 and 134 [COVID-19 DRUG TREATMENT] (74086)

136	(case study or case studies or case report? or case series).ti. (962261)

137	chart review?.ti. (4800)

138	editorial.ti. (159495)

139	135 not (136 or 137 or 138) [IRRELEVANT PUBLICATION TYPES REMOVED] (71435)

140	(randomi#ed or randomi#ation? or randomly or RCT or placebo*).ti,ab. (3038434)

141	((singl* or doubl* or trebl* or tripl*) adj (mask* or blind* or dumm*)).ti,ab. (510892)

142	trial.ti. (718162)

143	or/140-142 [RCTs] (3283584)

144	139 and 143 [COVID-19 DRUG TREATMENT - RCTs] (6673)

145	limit 144 to yr="2019-current" (6627)

146	145 use ipab [IPA RECORDS] (145)

147	48 or 106 or 146 [ALL DATABASES] (11183)

148	limit 147 to yr="2022-current" (5537)

149	remove duplicates from 148 (4034)

150	147 not 148 (5646)

151	remove duplicates from 150 (4055)

152	149 or 151 [TOTAL UNIQUE RECORDS] (8089)

153	152 use medall [MEDLINE UNIQUE RECORDS] (4020)

154	152 use emczd [EMBASE UNIQUE RECORDS] (4021)

155	152 use ipab [IPA UNIQUE RECORDS] (48)

***************************

CENTRAL

Search Name:

Date Run:	03/09/2023 18:18:57

Comment:

ID	Search	Hits

#1	[mh ^"COVID-19"]	4725

#2	("COVID-19" or COVID19):ti,ab,kw	16720

#3	((coronavirus* or corona virus*) and (hubei or wuhan or beijing or shanghai)):ti,ab,kw	319

#4	(wuhan NEAR/5 virus*):ti,ab,kw	10

#5	("2019-nCoV" or 19nCoV or 2019nCoV):ti,ab,kw	13

#6	("SARS-CoV-2" or "SARS-CoV2" or "SARSCoV-2" or SARSCoV2 or SARS2 or "SARS-2" or "severe acute respiratory syndrome coronavirus 2"):ti,ab,kw	6719

#7	("2019-novel CoV" or "Sars-coronavirus2" or "Sars-coronavirus-2" or "SARS-like coronavirus" or "SARS-like coronaviruses" or ((novel or new or nouveau) NEAR/2 (CoV or nCoV or covid or coronavirus* or "corona virus" or "pandemic 2"))):ti,ab,kw	1159

#8	("novel coronavirus" or "novel coronaviruses" or "novel corona virus" or "novel corona viruses" or "novel CoV"):ti,ab,kw	886

#9	((coronavirus* or "corona virus" or "corona viruses") NEXT ("19" or "2019")):ti,ab,kw	192

#10	("coronavirus 2" or "corona virus 2"):ti,ab,kw	1993

#11	(coronavirus* or "corona virus" or "corona viruses"):ti,kw	8003

#12	covid:ti,kw	13140

#13	("B.1.1.7" or "B.1.351" or "B.1.617" or "B.1.427" or "B.1.429"):ti,ab,kw	63

#14	("B.1.1.529" or "BA.1" or "BA.2" or "BA.2.86" or "BA.3" or "BA.4" or "BA.5" or "BQ.1" or "BQ.1.1" or XBB or "XBB.1.5" or "XBB.1.6"):ti,ab,kw	229

#15	("P.1" and (Brazil* or variant or variants)):ti,ab,kw	224

#16	(((alpha or beta or delta or eta or gamma or iota or kappa or lambda or omicron or zeta) NEAR/3 variant*) and (coronavirus* or "corona virus" or "corona viruses" or covid*)):ti,ab,kw	242

#17	{or #1-#16}	18210

#18	([mh Child] or [mh Infant]) not ([mh Adolescent] or [mh Adult])	54632

#19	#17 NOT #18	17998

#20	[mh "COVID-19 Drug Treatment"]	700

#21	[mh ^"Drug Therapy"]	14826

#22	(drug or drugs or medication or medications or medicines or pharmaceutic*):ti,kw	642789

#23	(pharmacotherap* or (pharmaco NEXT therap*)):ti,kw	3113

#24	(treat* or therap*):ti,kw	972659

#25	(paxlovid* or (nirmatrelvir* NEAR/2 ritonavir*)):ti,ab,kw	66

#26	(("pf 07321332" or pf07321332 or pf 7321332 or pf7321332) NEAR/2 ritonavir*):ti,ab,kw	10

#27	(remdesivir* or "gs 5734" or gs5734 or "gs 465124" or "gs 829143" or "ro 7286260" or ro7286260 or redyx* or veklury* or "UNII-3QKI37EEHE" or "1809249-37-3"):ti,ab,kw	412

#28	(molnupiravir* or "eidd 2801" or eidd2801 or "mk 4482" or mk4482 or lagevrio* or "UNII-YA84KI1VEW" or "2349386-89-4" or "2492423-29-5"):ti,ab,kw	84

#29	{or #21-#28}	1090957

#30	#19 AND #29	8383

#31	#20 or #30	8384

#32	(preprint or conference*):pt	226485

#33	#31 not #32 with Cochrane Library publication date Between Dec 2019 and Dec 2023	6779

Trials – 6730

CINAHL

| # | Query | Limiters/Expanders | Last Run Via | Results |
| --- | --- | --- | --- | --- |
| S41 | S33 AND S39 | Limiters - Published Date: 20190101-20231231  Search modes - Boolean/Phrase | Interface - EBSCOhost Research Databases  Search Screen - Advanced Search  Database - CINAHL Plus with Full Text | 912 |
| S40 | S33 AND S39 | Search modes - Boolean/Phrase | Interface - EBSCOhost Research Databases  Search Screen - Advanced Search  Database - CINAHL Plus with Full Text | 916 |
| S39 | S34 OR S35 OR S36 OR S37 OR S38 | Search modes - Boolean/Phrase | Interface - EBSCOhost Research Databases  Search Screen - Advanced Search  Database - CINAHL Plus with Full Text | 498,129 |
| S38 | TI trial | Search modes - Boolean/Phrase | Interface - EBSCOhost Research Databases  Search Screen - Advanced Search  Database - CINAHL Plus with Full Text | 183,699 |
| S37 | TI ( (singl* or doubl* or trebl* or tripl*) W0 (mask* or blind* or dumm*) ) OR AB ( (singl* or doubl* or trebl* or tripl*) W0 (mask* or blind* or dumm*) ) | Search modes - Boolean/Phrase | Interface - EBSCOhost Research Databases  Search Screen - Advanced Search  Database - CINAHL Plus with Full Text | 59,357 |
| S36 | TI ( randomi?ed or randomi?ation# or randomly or RCT or placebo* ) OR AB ( randomi?ed or randomi?ation# or randomly or RCT or placebo* ) | Search modes - Boolean/Phrase | Interface - EBSCOhost Research Databases  Search Screen - Advanced Search  Database - CINAHL Plus with Full Text | 410,915 |
| S35 | (MH "Double-Blind Studies") OR (MH "Single-Blind Studies") OR (MH "Triple-Blind Studies") | Search modes - Boolean/Phrase | Interface - EBSCOhost Research Databases  Search Screen - Advanced Search  Database - CINAHL Plus with Full Text | 70,383 |
| S34 | (MH "Randomized Controlled Trials+") | Search modes - Boolean/Phrase | Interface - EBSCOhost Research Databases  Search Screen - Advanced Search  Database - CINAHL Plus with Full Text | 138,949 |
| S33 | S27 NOT S32 | Search modes - Boolean/Phrase | Interface - EBSCOhost Research Databases  Search Screen - Advanced Search  Database - CINAHL Plus with Full Text | 9,072 |
| S32 | S28 OR S29 OR S30 OR S31 | Search modes - Boolean/Phrase | Interface - EBSCOhost Research Databases  Search Screen - Advanced Search  Database - CINAHL Plus with Full Text | 484,935 |
| S31 | TI chart W0 review# | Search modes - Boolean/Phrase | Interface - EBSCOhost Research Databases  Search Screen - Advanced Search  Database - CINAHL Plus with Full Text | 1,034 |
| S30 | TI "case study" or "case studies" or "case report" or "case reports" or "case series" | Search modes - Boolean/Phrase | Interface - EBSCOhost Research Databases  Search Screen - Advanced Search  Database - CINAHL Plus with Full Text | 109,166 |
| S29 | (MH "Case Studies") OR (MH "One-Shot Case Study") | Search modes - Boolean/Phrase | Interface - EBSCOhost Research Databases  Search Screen - Advanced Search  Database - CINAHL Plus with Full Text | 27,064 |
| S28 | PT editorial or newspaper | Search modes - Boolean/Phrase | Interface - EBSCOhost Research Databases  Search Screen - Advanced Search  Database - CINAHL Plus with Full Text | 353,904 |
| S27 | S17 AND S26 | Search modes - Boolean/Phrase | Interface - EBSCOhost Research Databases  Search Screen - Advanced Search  Database - CINAHL Plus with Full Text | 9,681 |
| S26 | S18 OR S19 OR S20 OR S21 OR S22 OR S23 OR S24 OR S25 | Search modes - Boolean/Phrase | Interface - EBSCOhost Research Databases  Search Screen - Advanced Search  Database - CINAHL Plus with Full Text | 843,708 |
| S25 | TI ( molnupiravir* or "eidd 2801" or eidd2801 or "mk 4482" or mk4482 or lagevrio* or "UNII-YA84KI1VEW" or "2349386-89-4" or "2492423-29-5" ) OR AB ( molnupiravir* or "eidd 2801" or eidd2801 or "mk 4482" or mk4482 or lagevrio* or "UNII-YA84KI1VEW" or "2349386-89-4" or "2492423-29-5" ) | Search modes - Boolean/Phrase | Interface - EBSCOhost Research Databases  Search Screen - Advanced Search  Database - CINAHL Plus with Full Text | 76 |
| S24 | TI ( remdesivir* or "gs 5734" or gs5734 or "gs 465124" or "gs 829143" or "ro 7286260" or ro7286260 or redyx* or veklury* or "UNII-3QKI37EEHE" or "1809249-37-3" ) OR AB ( remdesivir* or "gs 5734" or gs5734 or "gs 465124" or "gs 829143" or "ro 7286260" or ro7286260 or redyx* or veklury* or "UNII-3QKI37EEHE" or "1809249-37-3" ) | Search modes - Boolean/Phrase | Interface - EBSCOhost Research Databases  Search Screen - Advanced Search  Database - CINAHL Plus with Full Text | 650 |
| S23 | TI ( ("pf 07321332" or pf07321332 or pf 7321332 or pf7321332) N2 ritonavir* ) OR AB ( ("pf 07321332" or pf07321332 or pf 7321332 or pf7321332) N2 ritonavir* ) | Search modes - Boolean/Phrase | Interface - EBSCOhost Research Databases  Search Screen - Advanced Search  Database - CINAHL Plus with Full Text | 0 |
| S22 | TI ( paxlovid* or (nirmatrelvir* N2 ritonavir*) ) OR AB ( paxlovid* or (nirmatrelvir* N2 ritonavir*) ) | Search modes - Boolean/Phrase | Interface - EBSCOhost Research Databases  Search Screen - Advanced Search  Database - CINAHL Plus with Full Text | 173 |
| S21 | TI treat* or therap* | Search modes - Boolean/Phrase | Interface - EBSCOhost Research Databases  Search Screen - Advanced Search  Database - CINAHL Plus with Full Text | 617,159 |
| S20 | TI pharmacotherap* or (pharmaco W0 therap*) | Search modes - Boolean/Phrase | Interface - EBSCOhost Research Databases  Search Screen - Advanced Search  Database - CINAHL Plus with Full Text | 3,397 |
| S19 | TI drug or drugs or medication# or medicines or pharmaceutic* | Search modes - Boolean/Phrase | Interface - EBSCOhost Research Databases  Search Screen - Advanced Search  Database - CINAHL Plus with Full Text | 241,002 |
| S18 | (MH "Drug Therapy") | Search modes - Boolean/Phrase | Interface - EBSCOhost Research Databases  Search Screen - Advanced Search  Database - CINAHL Plus with Full Text | 16,620 |
| S17 | S1 OR S2 OR S3 OR S4 OR S5 OR S6 OR S7 OR S8 OR S9 OR S10 OR S11 OR S12 OR S13 OR S14 OR S15 OR S16 | Search modes - Boolean/Phrase | Interface - EBSCOhost Research Databases  Search Screen - Advanced Search  Database - CINAHL Plus with Full Text | 133,367 |
| S16 | TI ( (((alpha or beta or delta or eta or gamma or iota or kappa or lambda or omicron or zeta) N3 variant#) and (coronavirus* or "corona virus" or "corona viruses" or covid*)) ) OR AB ( (((alpha or beta or delta or eta or gamma or iota or kappa or lambda or omicron or zeta) N3 variant#) and (coronavirus* or "corona virus" or "corona viruses" or covid*)) ) | Search modes - Boolean/Phrase | Interface - EBSCOhost Research Databases  Search Screen - Advanced Search  Database - CINAHL Plus with Full Text | 876 |
| S15 | TI ( "P.1" and (Brazil* or variant#) ) OR AB ( "P.1" and (Brazil* or variant#) ) | Search modes - Boolean/Phrase | Interface - EBSCOhost Research Databases  Search Screen - Advanced Search  Database - CINAHL Plus with Full Text | 40 |
| S14 | TI ( "B.1.1.529" or "BA.1" or "BA.2" or "BA.2.86" or "BA.3" or "BA.4" or "BA.5" or "BQ.1" or "BQ.1.1" or XBB or "XBB.1.5" or "XBB.1.6" ) OR AB ( "B.1.1.529" or "BA.1" or "BA.2" or "BA.2.86" or "BA.3" or "BA.4" or "BA.5" or "BQ.1" or "BQ.1.1" or XBB or "XBB.1.5" or "XBB.1.6" ) | Search modes - Boolean/Phrase | Interface - EBSCOhost Research Databases  Search Screen - Advanced Search  Database - CINAHL Plus with Full Text | 306 |
| S13 | TI ( "B.1.1.7" or "B.1.351" or "B.1.617" or "B.1.427" or "B.1.429" ) OR AB ( "B.1.1.7" or "B.1.351" or "B.1.617" or "B.1.427" or "B.1.429" ) | Search modes - Boolean/Phrase | Interface - EBSCOhost Research Databases  Search Screen - Advanced Search  Database - CINAHL Plus with Full Text | 201 |
| S12 | TI COVID | Search modes - Boolean/Phrase | Interface - EBSCOhost Research Databases  Search Screen - Advanced Search  Database - CINAHL Plus with Full Text | 91,392 |
| S11 | TI coronavirus* or (corona W0 virus*) | Search modes - Boolean/Phrase | Interface - EBSCOhost Research Databases  Search Screen - Advanced Search  Database - CINAHL Plus with Full Text | 9,667 |
| S10 | TI ( "coronavirus 2" or "corona virus 2" ) OR AB ( "coronavirus 2" or "corona virus 2" ) | Search modes - Boolean/Phrase | Interface - EBSCOhost Research Databases  Search Screen - Advanced Search  Database - CINAHL Plus with Full Text | 5,718 |
| S9 | TI ( (coronavirus* or "corona virus" or "corona viruses") W0 ("19" or "2019") ) OR AB ( (coronavirus* or "corona virus" or "corona viruses") W0 ("19" or "2019") ) | Search modes - Boolean/Phrase | Interface - EBSCOhost Research Databases  Search Screen - Advanced Search  Database - CINAHL Plus with Full Text | 828 |
| S8 | TI ( (novel W0 coronavirus*) or ("novel corona" W0 virus*) or "novel CoV" ) OR AB ( (novel W0 coronavirus*) or ("novel corona" W0 virus*) or "novel CoV" ) | Search modes - Boolean/Phrase | Interface - EBSCOhost Research Databases  Search Screen - Advanced Search  Database - CINAHL Plus with Full Text | 3,051 |
| S7 | TI ( "2019-novel CoV" or "Sars-coronavirus2" or "Sars-coronavirus-2" or "SARS-like coronavirus" or "SARS-like coronaviruses" or ((novel or new or nouveau) N2 (CoV or nCoV or covid or coronavirus* or "corona virus" or "Pandemic 2")) ) OR AB ( "2019-novel CoV" or "Sars-coronavirus2" or "Sars-coronavirus-2" or "SARS-like coronavirus" or "SARS-like coronaviruses" or ((novel or new or nouveau) N2 (CoV or nCoV or covid or coronavirus* or "corona virus" or "Pandemic 2")) ) | Search modes - Boolean/Phrase | Interface - EBSCOhost Research Databases  Search Screen - Advanced Search  Database - CINAHL Plus with Full Text | 5,417 |
| S6 | TI ( "SARS-CoV-2" or "SARS-CoV2" or "SARSCoV-2" or SARSCoV2 or SARS2 or "SARS-2" or "severe acute respiratory syndrome coronavirus 2" ) OR AB ( "SARS-CoV-2" or "SARS-CoV2" or "SARSCoV-2" or SARSCoV2 or SARS2 or "SARS-2" or "severe acute respiratory syndrome coronavirus 2" ) | Search modes - Boolean/Phrase | Interface - EBSCOhost Research Databases  Search Screen - Advanced Search  Database - CINAHL Plus with Full Text | 20,205 |
| S5 | TI ( "2019-nCoV" or 19nCoV or 2019nCoV ) OR AB ( "2019-nCoV" or 19nCoV or 2019nCoV ) | Search modes - Boolean/Phrase | Interface - EBSCOhost Research Databases  Search Screen - Advanced Search  Database - CINAHL Plus with Full Text | 19,332 |
| S4 | TI wuhan N5 virus* OR AB wuhan N5 virus* | Search modes - Boolean/Phrase | Interface - EBSCOhost Research Databases  Search Screen - Advanced Search  Database - CINAHL Plus with Full Text | 82 |
| S3 | TI ( (coronavirus* or "corona virus" or "corona viruses") and (hubei or wuhan or beijing or shanghai) ) OR AB ( (coronavirus* or "corona virus" or "corona viruses") and (hubei or wuhan or beijing or shanghai) ) | Search modes - Boolean/Phrase | Interface - EBSCOhost Research Databases  Search Screen - Advanced Search  Database - CINAHL Plus with Full Text | 1,310 |
| S2 | TI ( "COVID-19" or COVID19 ) OR AB ( "COVID-19" or COVID19 ) | Search modes - Boolean/Phrase | Interface - EBSCOhost Research Databases  Search Screen - Advanced Search  Database - CINAHL Plus with Full Text | 116,679 |
| S1 | (MH "COVID-19") | Search modes - Boolean/Phrase | Interface - EBSCOhost Research Databases  Search Screen - Advanced Search  Database - CINAHL Plus with Full Text | 43,136 |

Web of Science (*Core Databases - excluding 2 conference databases*)

| Set # | Search Query | Results |
| --- | --- | --- |
| 1 | "COVID-19" or COVID19 (Topic) OR (coronavirus* or "corona virus" or "corona viruses") and (hubei or wuhan or beijing or shanghai) (Topic) OR wuhan NEAR/5 virus* (Topic) OR "2019-nCoV" or 19nCoV or 2019nCoV (Topic) OR "SARS-CoV-2" or "SARS-CoV2" or "SARSCoV-2" or SARSCoV2 or SARS2 or "SARS-2" or "severe acute respiratory syndrome coronavirus 2" (Topic) Editions: WOS.SCI,WOS.SSCI,WOS.AHCI,WOS.BSCI,WOS.BHCI,WOS.ESCI | 457469 |
| 2 | "2019-novel CoV" or "Sars-coronavirus2" or "Sars-coronavirus-2" or "SARS-like coronavirus" or "SARS-like coronaviruses" or ((novel or new or nouveau) NEAR/2 (CoV or nCoV or covid or coronavirus* or "corona virus" or "Pandemic 2")) (Topic) OR "novel coronavirus" or "novel corona virus" or "novel coronaviruses" or "novel corona viruses" or "novel CoV" (Topic) OR (coronavirus* or "corona virus" or "corona viruses") NEAR/0 ("19" or "2019") (Topic) OR "coronavirus 2" or "corona virus 2" (Topic) OR coronavirus* or "corona virus" or "corona viruses" (Title) OR COVID (Title) Editions: WOS.SCI,WOS.SSCI,WOS.AHCI,WOS.BSCI,WOS.BHCI,WOS.ESCI | 356692 |
| 3 | "B.1.1.7" or "B.1.351" or "B.1.617" or "B.1.427" or "B.1.429" (Topic) OR "B.1.1.529" or "BA.1" or "BA.2" or "BA.2.86" or "BA.3" or "BA.4" or "BA.5" or "BQ.1" or "BQ.1.1" or XBB or "XBB.1.5" or "XBB.1.6" (Topic) OR "P.1" and (Brazil* or variant or variants) (Topic) Editions: WOS.SCI,WOS.SSCI,WOS.AHCI,WOS.BSCI,WOS.BHCI,WOS.ESCI | 16762 |
| 4 | (((alpha or beta or delta or eta or gamma or iota or kappa or lambda or omicron or zeta) NEAR/3 variant*) and (coronavirus* or "corona virus" or "corona viruses" or covid*)) (Topic) Editions: WOS.SCI,WOS.SSCI,WOS.AHCI,WOS.BSCI,WOS.BHCI,WOS.ESCI | 6191 |
| 5 | #4 OR #3 OR #2 OR #1 Editions: WOS.SCI,WOS.SSCI,WOS.AHCI,WOS.BSCI,WOS.BHCI,WOS.ESCI | 488334 |
| 6 | drug or drugs or medication or medications or medicines or pharmaceutic* (Title) OR pharmacotherap* or (pharmaco NEAR/0 therap*) (Title) OR treat* or therap* (Title) Editions: WOS.SCI,WOS.SSCI,WOS.AHCI,WOS.BSCI,WOS.BHCI,WOS.ESCI | 3895685 |
| 7 | paxlovid* or (nirmatrelvir* NEAR/2 ritonavir*) (Topic) OR remdesivir* or "gs 5734" or gs5734 or "gs 465124" or "gs 829143" or "ro 7286260" or ro7286260 or redyx* or veklury* or "UNII-3QKI37EEHE" or "1809249-37-3" (Topic) OR molnupiravir* or "eidd 2801" or eidd2801 or "mk 4482" or mk4482 or lagevrio* or "UNII-YA84KI1VEW" or "2349386-89-4" or "2492423-29-5" (Topic) Editions: WOS.SCI,WOS.SSCI,WOS.AHCI,WOS.BSCI,WOS.BHCI,WOS.ESCI | 4504 |
| 8 | #7 OR #6 Editions: WOS.SCI,WOS.SSCI,WOS.AHCI,WOS.BSCI,WOS.BHCI,WOS.ESCI | 3898197 |
| 9 | #8 AND #5 Editions: WOS.SCI,WOS.SSCI,WOS.AHCI,WOS.BSCI,WOS.BHCI,WOS.ESCI | 35314 |
| 10 | "case study" or "case studies" or "case report" or "case reports" or "case series" (Title) Editions: WOS.SCI,WOS.SSCI,WOS.AHCI,WOS.BSCI,WOS.BHCI,WOS.ESCI | 591553 |
| 11 | "chart review" or "chart reviews" (Title) Editions: WOS.SCI,WOS.SSCI,WOS.AHCI,WOS.BSCI,WOS.BHCI,WOS.ESCI | 2568 |
| 12 | #11 OR #10 Editions: WOS.SCI,WOS.SSCI,WOS.AHCI,WOS.BSCI,WOS.BHCI,WOS.ESCI | 594107 |
| 13 | #9 NOT #12 Editions: WOS.SCI,WOS.SSCI,WOS.AHCI,WOS.BSCI,WOS.BHCI,WOS.ESCI | 34292 |
| 14 | randomised or randomisation* or randomized or randomization or randomly or RCT or placebo* (Title) OR randomised or randomisation* or randomized or randomization or randomly or RCT or placebo* (Abstract) OR (singl* or doubl* or trebl* or tripl*) NEAR/0 (mask* or blind* or dumm*) (Title) OR (singl* or doubl* or trebl* or tripl*) NEAR/0 (mask* or blind* or dumm*) (Abstract) OR trial (Title) Editions: WOS.SCI,WOS.SSCI,WOS.AHCI,WOS.BSCI,WOS.BHCI,WOS.ESCI | 1650647 |
| 15 | #14 AND #13 Editions: WOS.SCI,WOS.SSCI,WOS.AHCI,WOS.BSCI,WOS.BHCI,WOS.ESCI | 2977 |
| 16 | #14 AND #13 and 2023 or 2022 or 2021 or 2020 or 2019 (Publication Years) Editions: WOS.SCI,WOS.SSCI,WOS.AHCI,WOS.BSCI,WOS.BHCI,WOS.ESCI | 2944 |
