# Supplementary material for: Effectiveness of oral and inhaled repurposed and antiviral pharmaceutical treatments for acute SARS-CoV-2 in outpatient settings: a systematic review of 124 randomized trials
Source: BMC Infect Dis. 2026 Jul 23;26:1829. doi: 10.1186/s12879-026-13901-7 (PMC13682097; doi:10.1186/s12879-026-13901-7)
Supplement: Supplementary file 3 [file 12879_2026_13901_MOESM3_ESM.docx]

**Supplementary File 2. Tables 1-5**

**Table 1a:** Effect of repurposed treatments on time to recovery

| **Treatment Name** | **Comparator category** | **Study, country** | **Tx classification** | **Treatment (N)** | **Comparator (N)** | **Unique population** | **Tx dosage and duration** | **Type of comparator** | **Outcome Definition** | **Effect measure** | **Effect estimate** | **Notes** |
| --- | --- | --- | --- | --- | --- | --- | --- | --- | --- | --- | --- | --- |
| Azithromycin + Standard/Usual Care | Standard/Usual Care | Butler 2021, UK | RP | 500 | 629 | 1 | Oral Azithromycin 500 mg once daily for three days plus usual care | Usual care | Time to sustained recovery  (days) | HR 95% Bayseian credible interval | 0·94 (0·81 to 1·09) | n value used for Azithromycin in primary analysis population and n value used for Usual care in concurrent randomisation analysis population. Estimated HR derived from a Cox proportional hazard model adjusted for age, comorbidities  at baseline, duration of illness, and eligibility for azithromycin at baseline, with 95% CI |
| Azithromycin + Standard/Usual Care | Standard/Usual Care | Butler 2021, UK | RP | 500 | 629 | 1 | Oral Azithromycin 500 mg once daily for three days plus usual care | Usual care | Time to alleviation of all  symptoms (days) | HR 95% Bayseian credible interval | 1·04 (0·91 to 1·19) | n value used for Azithromycin in primary analysis population and n value used for Usual care in concurrent randomisation analysis population. Estimated HR derived from a Cox proportional hazard model adjusted for age, comorbidities  at baseline, duration of illness, and eligibility for azithromycin at baseline, with 95% CI |
| Budesonide | Standard/Usual Care | Ramakrishnan. 2021, UK | RP | 71 | 69 | 0 | Budesonide dry powder inhaler (two puffs to be taken twice per day; total dose 1600 μg) | Usual care | Self-reported clinical recovery | Tx vs comparator; Median days [95% CI], log rank test; p-valur | 7 days [6-9] vs. 8 days [7-11]; p=0.007 |  |
| Budesonide | Standard/Usual Care | Ramakrishnan. 2021, UK | RP | 71 | 69 | 0 | Budesonide dry powder inhaler (two puffs to be taken twice per day; total dose 1600 μg) | Usual care | Mean time to recovery | Tx vs comparator; Mean (SD) | 8 days (5) vs. 12 days (8) |  |
| Budesonide | Standard/Usual Care | Ramakrishnan. 2021, UK | RP | 71 | 69 | 0 | Budesonide dry powder inhaler (two puffs to be taken twice per day; total dose 1600 μg) | Usual care | Median times to recovery (clinical recovery in participants with confirmed SARS-CoV-2) | Tx vs comparator; Median days [95% CI], p-value | 7 days [6–9] vs 8 days [7–10]; p=0·012 |  |
| Budesonide | Standard/Usual Care | Yu, UK, 2021 | RP | 833 | 1126 | 1 | 800 μg Budesonide twice daily for 14 days | Usual care + other inventions | Time to first self-reported recovery | Hazard Ratio (HR), 95% BCI | 1.21 [ 1.08 to 1.36]) |  |
| Camostat | Placebo | Jilg, 2023, USA | RP | 109 | 107 | 0 | 200 mg oral camostat (2 tablets, 100 mg each) every 6 hours for 7 days | 200 mg oral placebo (2 tablets, 100 mg each) every 6 hours for 7 days | Time to 2 consecutive days of improvement in all targeted symptoms | Camostat vs. Placebo:   Median (Q1, Q3) days  P-value | 9 (5, 19) vs. 9 (9,19)  P=0.99 |  |
| Camostat | Placebo | Jilg, 2023, USA | RP | 109 | 107 | 0 | 200 mg oral camostat (2 tablets, 100 mg each) every 6 hours for 7 days | 200 mg oral placebo (2 tablets, 100 mg each) every 6 hours for 7 days | Time (days) to returning to usual (pre-COVID) health for 2 consecutive days | Camostat vs. Placebo:   Median days (IQR)  P-value | 12 (7, > 27) vs. 14 (7, 26) p=0.84 |  |
| Camostat | Placebo | Jilg, 2023, USA | RP | 109 | 107 | 0 | 200 mg oral camostat (2 tablets, 100 mg each) every 6 hours for 7 days | 200 mg oral placebo (2 tablets, 100 mg each) every 6 hours for 7 days | Time (days) to returning to usual (pre-COVID) health for 4 consecutive days | Camostat vs. Placebo:   Median days (IQR)  P-value | 15 (8, >25) vs. 15 (7, >25) p=0.47 |  |
| Ciclesonide | Placebo | Ezer 2021, Canada | RP | 105 | 98 | 1 | inhaled ciclesonide (600 μg twice daily) and intranasal ciclesonide (200 μg daily) for 14 days. | metered dose inhaler and nasal saline placebos for 14 days | Overall feeling of improvement; Day 7 | Adjusted risk difference‡, % (95% CI) | −2.1 (−14.2 to 9.9) | ‡Adjusted for stratification on sex |
| Ciclesonide | Placebo | Ezer 2021, Canada | RP | 105 | 98 | 1 | inhaled ciclesonide (600 μg twice daily) and intranasal ciclesonide (200 μg daily) for 14 days. | metered dose inhaler and nasal saline placebos for 14 days | Overall feeling of improvement; Day 14 | Adjusted risk difference‡, % (95% CI) | −2.4 (−10.0 to 5.1) | ‡Adjusted for stratification on sex |
| Colchicine + Standard/Usual Care | Standard/Usual Care | Dorward 2022, UK | RP | 156 | 1145 | 0 | usual care plus colchicine 500 µg daily for 14 days | usual care alone daily for 14 days | time to first-reported recovery defined as the first instance that a participant reports feeling recovered; and | HR 95% CrI | 0.92 (0.72 to 1.16) | n value used from SARS-CoV-2-positive analysis population. Bayesian primary analysis |
| Doxycycline + Standard/Usual Care | Standard/Usual Care | Butler 2021, UK | RP | 435 | 336 | 0 | usual care plus oral doxycycline (200 mg on day 1, followed by 100 mg once daily for the following 6 days) | usual care only: largely supportive. | Time to first reported recovery in days | Estimated benefit, median (95% BCI) Bayesian credible interval | 0·70 (–1·45 to 3·03) | n value used is in SARS-CoV-2 PCR-positive population. Estimated benefit in median time to recovery derived from a Bayesian piecewise exponential model adjusted for age and comorbidity at baseline; a positive value in estimated benefit in median time to recovery (or HR >1) corresponds to a reduction in time to recovery in days with usual care plus doxycycline compared with usual care only. Model-based estimates in the SARS-CoV-2 PCR-positive population (n=435 in the usual care plus doxycycline group and n=336 in the usual care only group) |
| Doxycycline + Standard/Usual Care | Standard/Usual Care | Butler 2021, UK | RP | 780 | 644 | 0 | usual care plus oral doxycycline (200 mg on day 1, followed by 100 mg once daily for the following 6 days) | usual care only: largely supportive. | Time to sustained recovery in days | HR (95% CI) | 1·00 (0·88 to 1·14) | n value from concurrent randomisation analysis population. Estimated hazard ratio derived from a Cox proportional hazards model adjusted for age, comorbidity at baseline, duration of illness, and eligibility for doxycycline at baseline. |
| Dutasteride | Placebo | Cadegiani, Brazil, 2021 | RP | 43 | 44 | 1 | 0.5 mg/day for 30 days with nitazoxanide 500 mg twice a day for six days and azithromycin 500 mg/day for five days. | Placebo pill 0.5 mg/day for 30 days with nitazoxanide 500 mg twice a day for six days and azithromycin 500 mg/day for five days. | Proportion (%) fully clinically recovered on day 7, determined through a report of perceived recovery scale from 0 to 100 | Tx vs comparator; Proportion with RT-PCR negative | 84.1% vs 57.5%, p = 0.03 |  |
| Dutasteride | Placebo | Cadegiani, Brazil, 2021 | RP | 43 | 44 | 1 | 0.5 mg/day for 30 days with nitazoxanide 500 mg twice a day for six days and azithromycin 500 mg/day for five days. | Placebo pill 0.5 mg/day for 30 days with nitazoxanide 500 mg twice a day for six days and azithromycin 500 mg/day for five days. | Clinically recovered on Day 1 | Tx vs comparator; mean (SD) | 60.4 (24.2) vs 34.2 (21.4) ; p<0.0001 |  |
| Dutasteride | Placebo | Cadegiani, Brazil, 2021 | RP | 43 | 44 | 1 | 0.5 mg/day for 30 days with nitazoxanide 500 mg twice a day for six days and azithromycin 500 mg/day for five days. | Placebo pill 0.5 mg/day for 30 days with nitazoxanide 500 mg twice a day for six days and azithromycin 500 mg/day for five days. | Clinically recovered on Day 2 | Tx vs comparator; mean (SD) | 78.5 (±17.8) vs 52.9 (±21.3); p<0.0001 |  |
| Dutasteride | Placebo | Cadegiani, Brazil, 2021 | RP | 43 | 44 | 1 | 0.5 mg/day for 30 days with nitazoxanide 500 mg twice a day for six days and azithromycin 500 mg/day for five days. | Placebo pill 0.5 mg/day for 30 days with nitazoxanide 500 mg twice a day for six days and azithromycin 500 mg/day for five days. | Clinically recovered on Day 3 | Tx vs comparator; mean (SD) | 89.2 (12.3) vs 66.8 (20.7); p<0.0001 |  |
| Dutasteride | Placebo | Cadegiani, Brazil, 2021 | RP | 43 | 44 | 1 | 0.5 mg/day for 30 days with nitazoxanide 500 mg twice a day for six days and azithromycin 500 mg/day for five days. | Placebo pill 0.5 mg/day for 30 days with nitazoxanide 500 mg twice a day for six days and azithromycin 500 mg/day for five days. | Clinically recovered on Day 7 | Tx vs comparator; mean (SD) | 97.4 (5.7) vs 82.9 (15.0) p<0.0001 |  |
| Fluticasone Furoate | Placebo | Boulware, USA, 2023 | RP | 656 | 621 | 1 | 200 μg once daily for 14 days | Matching placebo once daily for 14 days | Time to recovery (third of 3 consecutive days without symptoms) | HR (95% Credible interval) | 1.01 (0.91 to 1.12); p=0.56 | Unique patient population: 30 years or older |
| Fluticasone Furoate | Placebo | Boulware, USA, 2023 | RP | 656 | 621 | 1 | 200 μg once daily for 14 days | Matching placebo once daily for 14 days | Covid-19 clinical progression ordinal  outcome scale, Day 7 | OR (95% Credible interval) | 1.10 (0.62 to 1.63), p=0.41 | Unique patient population: 30 years or older |
| Fluticasone Furoate | Placebo | Boulware, USA, 2023 | RP | 656 | 621 | 1 | 200 μg once daily for 14 days | Matching placebo once daily for 14 days | Covid-19 clinical progression ordinal  outcome scale, Day 14 | OR (95% Credible interval) | 0.91 (0.42 to 1.50), p=0.67 | Unique patient population: 30 years or older |
| Fluticasone Furoate | Placebo | Boulware, USA, 2023 | RP | 656 | 621 | 1 | 200 μg once daily for 14 days | Matching placebo once daily for 14 days | Covid-19 clinical progression ordinal  outcome scale, Day 28 | OR (95% Credible interval) | 2.74 (0.50 to 5.94), p=0.06 | Unique patient population: 30 years or older |
| Fluticasone Furoate | Placebo | Boulware, USA, 2023 | RP | 656 | 621 | 1 | 200 μg once daily for 14 days | Matching placebo once daily for 14 days | Mean no. of days unwell | Mean difference (95% Credible interval) | −0.10 (−0.45 to 0.26), p=0.7 | Unique patient population: 30 years or older |
| Fluvoxamine | Placebo | Stewart, 2023, USA | RP | 589 | 586 | 1 | 50 mg (one 50-mg tablet) Fluvoxamine twice daily for 1 day, followed by 100 mg (two 50-mg tablets) | Matching placebo twice daily for 1 day (two 50-mg tablets) or matching placebo twice daily for 12 days, for a total 13-day course | Time to sustained recovery | Tx vs comparator; Median days   95% CI  Posterior probability for benefit  HR (95% CI) | 10 days (10-11) vs. 10 days (10-11) p=0.4   0.99 ( 0.89-1.09) | Unique patient population: 30 years or older |
| Fluvoxamine | Placebo | Stewart, 2023, USA | RP | 589 | 586 | 1 | 51 mg (one 50-mg tablet) Fluvoxamine twice daily for 1 day, followed by 100 mg (two 50-mg tablets) | Matching placebo twice daily for 1 day (two 50-mg tablets) or matching placebo twice daily for 12 days, for a total 13-day course | Difference in mean time unwell | Tx vs comparator; Median days (95% CI)  p-value | 1.3 [11.1-11.5] days vs 11.5 [11.2-11.7]  p=0.79 | Unique patient population: 30 years or older |
| Fluvoxamine + Budesonide | Placebo | Reis, Brazil, 2023 | RP | 738 | 738 | 0 | fluvoxamine (100 mg twice daily for 10 days) plus inhaled budesonide (800 mcg twice daily for 10 days) | matching placebos | Time to recovery (self-reported) | HR (95% CrI) | 1.01 (0.91-1.13) |  |
| Fluvoxamine + favipiravir; Fluvoxamine + favipiravir + dexamethasone | Favipiravir | Siripongboonsitti, 2023, Thailand | RP | 134 | 132 | 1 | 50 mg twice daily of fluvoxamine for 10 days and favipiravir; patients with moderate illness also received 6 mg per day of dexamethasone for 10 days | Each group received 3600 mg of FPV loading dose on day 0 and 1600 mg per day of FPV from days 1-4 in mild COVID-19 and days 1-9 in moderate COVID-19. In a patient with obesity, the FPV loading dose was 4800 mg on day 0, then 2000 mg per day of FPV on days later | WHO clinical progression scale improvement day 2, mild illness | Tx vs comparator, N (%) vs N (%), p-value | 45 (34.09) vs 53 (39.55), p=0.356 |  |
| Fluvoxamine + favipiravir; Fluvoxamine + favipiravir + dexamethasone | Favipiravir | Siripongboonsitti, 2023, Thailand | RP | 134 | 132 | 1 | 50 mg twice daily of fluvoxamine for 10 days and favipiravir; patients with moderate illness also received 6 mg per day of dexamethasone for 10 days | Each group received 3600 mg of FPV loading dose on day 0 and 1600 mg per day of FPV from days 1-4 in mild COVID-19 and days 1-9 in moderate COVID-19. In a patient with obesity, the FPV loading dose was 4800 mg on day 0, then 2000 mg per day of FPV on days later | WHO clinical progression scale improvement day 5, mild illness | Tx vs comparator, N (%) vs N (%), p-value | 79 (59.85) vs 76 (56.72), p=0.604 |  |
| Fluvoxamine + favipiravir; Fluvoxamine + favipiravir + dexamethasone | Favipiravir | Siripongboonsitti, 2023, Thailand | RP | 134 | 132 | 1 | 50 mg twice daily of fluvoxamine for 10 days and favipiravir; patients with moderate illness also received 6 mg per day of dexamethasone for 10 days | Each group received 3600 mg of FPV loading dose on day 0 and 1600 mg per day of FPV from days 1-4 in mild COVID-19 and days 1-9 in moderate COVID-19. In a patient with obesity, the FPV loading dose was 4800 mg on day 0, then 2000 mg per day of FPV on days later | WHO clinical progression scale improvement day 14, mild illness | Tx vs comparator, N (%) vs N (%), p-value | 114 (86.36) vs 113 (84.33), p=0.639 |  |
| Fluvoxamine + favipiravir; Fluvoxamine + favipiravir + dexamethasone | Favipiravir | Siripongboonsitti, 2023, Thailand | RP | 134 | 132 | 1 | 50 mg twice daily of fluvoxamine for 10 days and favipiravir; patients with moderate illness also received 6 mg per day of dexamethasone for 10 days | Each group received 3600 mg of FPV loading dose on day 0 and 1600 mg per day of FPV from days 1-4 in mild COVID-19 and days 1-9 in moderate COVID-19. In a patient with obesity, the FPV loading dose was 4800 mg on day 0, then 2000 mg per day of FPV on days later | WHO clinical progression scale improvement day 2, moderate illness | Tx vs comparator, N (%) vs N (%), p-value | 9 (30) vs 5 (16.13), p=0.198 |  |
| Fluvoxamine + favipiravir; Fluvoxamine + favipiravir + dexamethasone | Favipiravir | Siripongboonsitti, 2023, Thailand | RP | 134 | 132 | 1 | 50 mg twice daily of fluvoxamine for 10 days and favipiravir; patients with moderate illness also received 6 mg per day of dexamethasone for 10 days | Each group received 3600 mg of FPV loading dose on day 0 and 1600 mg per day of FPV from days 1-4 in mild COVID-19 and days 1-9 in moderate COVID-19. In a patient with obesity, the FPV loading dose was 4800 mg on day 0, then 2000 mg per day of FPV on days later | WHO clinical progression scale improvement day 5, moderateillness | Tx vs comparator, N (%) vs N (%), p-value | 11 (36.67) vs 9 (29.03), p=0.420 |  |
| Fluvoxamine + favipiravir; Fluvoxamine + favipiravir + dexamethasone | Favipiravir | Siripongboonsitti, 2023, Thailand | RP | 134 | 132 | 1 | 50 mg twice daily of fluvoxamine for 10 days and favipiravir; patients with moderate illness also received 6 mg per day of dexamethasone for 10 days | Each group received 3600 mg of FPV loading dose on day 0 and 1600 mg per day of FPV from days 1-4 in mild COVID-19 and days 1-9 in moderate COVID-19. In a patient with obesity, the FPV loading dose was 4800 mg on day 0, then 2000 mg per day of FPV on days later | WHO clinical progression scale improvement day 14, moderate illness | Tx vs comparator, N (%) vs N (%), p-value | 26 (86.67) vs 27 (87.1), p=1.0 |  |
| Fluvoxamine + Standard/Usual Care | Placebo + Standard/Usual Care | Ibrahim, 2023, Egypt | RP | 37; moderate: 51 | 35; moderate: 39 | 0 | standard therapy plus fluvoxamine at a dose of 100 mg twice daily for 10 days. | favipiravir (nonspecific antiviral drug) plus nonspecific antibiotics as a standard regimen in our study according to local recommendations | clinical deterioration (desaturation to <93% and/or need for hospitalization due to COVID-related illness), improved among mild cases | Tx vs comparator; N (%) vs. N(%) | 36 (97.3) vs 32 (91.4) | Mild: non-pneumonic; Moderate: pneumonic - reported as "improved" |
| Fluvoxamine + Standard/Usual Care | Placebo + Standard/Usual Care | Ibrahim, 2023, Egypt | RP | 37; moderate: 51 | 35; moderate: 39 | 0 | standard therapy plus fluvoxamine at a dose of 100 mg twice daily for 10 days. | favipiravir (nonspecific antiviral drug) plus nonspecific antibiotics as a standard regimen in our study according to local recommendations | clinical deterioration (desaturation to <93% and/or need for hospitalization due to COVID-related illness), improved among moderate cases | Tx vs comparator; N (%) vs. N(%) | 45 (88.2) vs 28 (71.8) | Mild: non-pneumonic; Moderate: pneumonic; reported as "improved" |
| Ivermectin | Placebo | Naggie, 2022, US | RP | 817 | 774 | 0 | 400 μg/kg daily for 3 days | Placebo | Time to recovery (achieving at least 3 consecutive days without symptoms) | Hazard ratio (HR), 95% CrI | 1.07, 0.96-1.17 |  |
| Ivermectin | Placebo | Naggie, 2022, US | RP | 817 | 774 | 0 | 400 μg/kg daily for 3 days | Placebo | Clinical progression ordinal outcome scale, Day 7 | OR (95% CrI) | OR, 0.81 (0.50 to 1.13) |  |
| Ivermectin | Placebo | Naggie, 2022, US | RP | 817 | 774 | 0 | 400 μg/kg daily for 3 days | Placebo | Clinical progression ordinal outcome scale, Day 14 | OR (95% CrI) | OR, 0.76 (0.39 to 1.13) |  |
| Ivermectin | Placebo | Naggie, 2022, US | RP | 817 | 774 | 0 | 400 μg/kg daily for 3 days | Placebo | Clinical progression ordinal outcome scale, Day 28 | OR (95% CrI) | OR, 1.11 (0.52 to 1.91) |  |
| Ivermectin | Placebo | Naggie, 2022, US | RP | 817 | 774 | 0 | 400 μg/kg daily for 3 days | Placebo | Time unwell | Mean difference (95% CrI) | −0.49 (−0.82 to −0.15) |  |
| Ivermectin | Placebo; Fluvoxamine | Naggie, 2023, US | RP | 708 | 724 | 0 | dosing schedule was based on weight ranges as follows: those weighing 35 to 52 kg received a 21-mg daily dose ivermectin; 53 to 69 kg, 28-mg daily dose; 70 to 89 kg, 42-mg daily dose; 90 to 109 kg, 49-mg daily dose; 110 to 129 kg, 56-mg daily dose; and more than 129 kg, 70-mg daily dose | placebo, fluvoxamine 50mg twice daily | Time unwell | mean difference (95% CrI) | -0.04 (-0.39, 0.32) |  |
| Ivermectin | Placebo; Fluvoxamine | Naggie, 2023, US | RP | 708 | 724 | 0 | dosing schedule was based on weight ranges as follows: those weighing 35 to 52 kg received a 21-mg daily dose ivermectin; 53 to 69 kg, 28-mg daily dose; 70 to 89 kg, 42-mg daily dose; 90 to 109 kg, 49-mg daily dose; 110 to 129 kg, 56-mg daily dose; and more than 129 kg, 70-mg daily dose | placebo, fluvoxamine 50mg twice daily | COVID-19 clinical progression (ordinal outcome scale), day 7 | OR (95% CrI) | 1.29 (0.73, 1.89) | N = 1370 |
| Ivermectin | Placebo; Fluvoxamine | Naggie, 2023, US | RP | 708 | 724 | 0 | dosing schedule was based on weight ranges as follows: those weighing 35 to 52 kg received a 21-mg daily dose ivermectin; 53 to 69 kg, 28-mg daily dose; 70 to 89 kg, 42-mg daily dose; 90 to 109 kg, 49-mg daily dose; 110 to 129 kg, 56-mg daily dose; and more than 129 kg, 70-mg daily dose | placebo, fluvoxamine 50mg twice daily | COVID-19 clinical progression (ordinal outcome scale), day 14 | OR (95% CrI) | 2.50 (0.90, 3.44) | N = 1359 |
| Ivermectin | Placebo; Fluvoxamine | Naggie, 2023, US | RP | 708 | 724 | 0 | dosing schedule was based on weight ranges as follows: those weighing 35 to 52 kg received a 21-mg daily dose ivermectin; 53 to 69 kg, 28-mg daily dose; 70 to 89 kg, 42-mg daily dose; 90 to 109 kg, 49-mg daily dose; 110 to 129 kg, 56-mg daily dose; and more than 129 kg, 70-mg daily dose | placebo, fluvoxamine 50mg twice daily | COVID-19 clinical progression (ordinal outcome scale), day 28 | OR (95% CrI) | 2.50 (0.86 to 4.68) |  |
| Ivermectin + doxycycline + Standard/Usual Care | Placebo + Standard/Usual Care | Mahmud 2021, Bangladesh | RP | 183 | 180 | 0 | Ivermectin 12 mg and doxycycline 100mg, twice daily for 5 days, in addition to standard of care. (Standard of care included administration of paracetamol, antihistamines, cough suppressants, vitamins, oxygen therapy according to indication and need, low molecular weight heparin according to indication, appropriate other broad-spectrum antibiotics, remdesivir injection, other antiviral drugs, and other drugs for associated comorbid conditions.) | The placebo group received placebo in addition to standard of care | Patients recovery or responding to treatment within 7 days | Hazard ratio (95% CI) | 0.06 (0.04–0.09) |  |
| Ivermectin + doxycycline + Standard/Usual Care | Placebo + Standard/Usual Care | Mahmud 2021, Bangladesh | RP | 183 | 180 | 0 | Ivermectin 12 mg and doxycycline 100mg, twice daily for 5 days, in addition to standard of care. (Standard of care included administration of paracetamol, antihistamines, cough suppressants, vitamins, oxygen therapy according to indication and need, low molecular weight heparin according to indication, appropriate other broad-spectrum antibiotics, remdesivir injection, other antiviral drugs, and other drugs for associated comorbid conditions.) | The placebo group received placebo in addition to standard of care | Patients recovery or responding to treatment within 7–11 days | Hazard ratio (95% CI) | 1.02 (0.77–1.36) |  |
| Ivermectin + Standard/Usual Care | Standard/Usual Care | Hayward, 2024, UK | RP | 2250 | 1869 | 0 | Participants were randomised to usual care plus ivermectin tablets (target 300–400 μg/kg per dose, once daily for 3 days) or usual care plus other interventions | Usual care | Time to first recovery | Hazard ratio   95% Bayesian credible interval  p-value | 1.15 (1.07 - 1.23)  p=>0.999 | NOTE: Based on "Concurrently and eligible randomized population" |
| Ivermectin + Standard/Usual Care | Standard/Usual Care | Hayward, 2024, UK | RP | 2250 | 1869 | 0 | Participants were randomised to usual care plus ivermectin tablets (target 300–400 μg/kg per dose, once daily for 3 days) or usual care plus other interventions | Usual care | Median time to recovery of 16 days | Ivermectin vs. usual care:  Hazard ratio   95% Bayesian credible interval | 2.055 (0.999 to 3.06) | NOTE: Based on "Concurrently and eligible randomized population" |
| Ivermectin + Standard/Usual Care | Standard/Usual Care | Hayward, 2024, UK | RP | 2250 | 1869 | 0 | Participants were randomised to usual care plus ivermectin tablets (target 300–400 μg/kg per dose, once daily for 3 days) or usual care plus other interventions | Usual care | Time to sustained recovery (days), median IQR | Estimated difference median | 1.19 (1.09 to 1.29) | NOTE: Based on "Concurrently and eligible randomized population" |
| Oral N-acetylcysteine + Standard/Usual Care; Bromhexine tablets + Standard/Usual Care | Standard/Usual Care | Eslami-Ghayour, 2024, Iran | RP | Group A: 75 Group B: 75 | 75 | 0 | Group A: Oral N-acetylcysteine, 600 mg twice a day for five days + usual treatment  Group B: Bromhexine tablets, 8 mg three times a day for five days + usual treatment | Group C: Only usual treatment (Naproxen, 250 mg twice daily for five days, famotidine, 20 mg once daily for 10 days, vitamin D 50,000 per week for four weeks, vitamin C, 1000 mg daily) | Mean recovery time (days) | One-Way ANOVA, p-value (95% CI) | Group A vs. B vs. C:   12.65 (± 0.90) vs. 10.76(± 0.64) vs. 15.04 (± 8.557)  p-value < 0.001 | Note: ITT analysis was not explicitly mentioned in this trial. |
| SNG001 | Placebo | Jagannathan, 2023, US | RP | 110 | 110 | 0 | SNG001, 12 MIU/mL, single nebulized dose, once a day, for 14 days | Placebo solution, single nebulized dose, once day, for 14 days | Time (days) to returning to usual (pre-COVID) health for 3 consecutive days | Tx vs comparator; Median days (Q1,Q3)  p-value | 19 (11, >27) vs 17 (8, >27)  p=0.18 |  |
| SNG001 | Placebo | Jagannathan, 2023, US | RP | 110 | 110 | 0 | SNG001, 12 MIU/mL, single nebulized dose, once a day, for 14 days | Placebo solution, single nebulized dose, once day, for 14 days | Time (days) to returning to usual (pre-COVID) health for 4 consecutive days | Tx vs comparator; Median days (Q1,Q3)  p-value | 21 (12, >25) vs 19 (9, >25)  p=0.43 |  |
| Tafenoquine | Placebo | Dow, 2022, USA | RP | 45 | 41 | 0 | Tafenoquine administered orally as 2 × 100 mg tablets on study days 1, 2, 3, and 10 | Matching placebo | Day 14 Clinical Recovery (ITT) - Patients not recovered | Tx vs. Comparator:     N (%) vs. N(%) | 8 (17.8%) vs. 10 (24.4%) |  |
| Tafenoquine | Placebo | Dow, 2022, USA | RP | 45 | 41 | 0 | Tafenoquine administered orally as 2 × 100 mg tablets on study days 1, 2, 3, and 10 | Matching placebo | Day 14 Clinical Recovery (ITT) - Patients recovered | Tx vs comparator:  N (%, 95% CI) vs. N (%, 95% CI) | 37 (82.2%, 68-92%) vs. 31 (75.6%, 60-88%) |  |
| Tafenoquine | Placebo | Dow, 2022, USA | RP | 45 | 41 | 0 | Tafenoquine administered orally as 2 × 100 mg tablets on study days 1, 2, 3, and 10 | Matching placebo | Day 14 Clinical Recovery (ITT) - Change in proportion unrecovered | Tx vs. Comparator:  %, p-value | 27%, p=0.6 |  |
| Tafenoquine | Placebo | Dow, 2022, USA | RP | 45 | 41 | 0 | Tafenoquine administered orally as 2 × 100 mg tablets on study days 1, 2, 3, and 10 | Matching placebo | Day 28 Clinical Recovery (ITT) - Patients not recovered | Tx vs. Comparator:     N (%) vs. N(%) | 7 (15.5%) vs. 9 (22%) |  |
| Tafenoquine | Placebo | Dow, 2022, USA | RP | 45 | 41 | 0 | Tafenoquine administered orally as 2 × 100 mg tablets on study days 1, 2, 3, and 10 | Matching placebo | Day 28 Clinical Recovery (ITT) - Patients recovered | Tx vs comparator:  N (%, 95% CI) vs. N (%, 95% CI) | 38 (84%, 71-94%) vs. 32 (78%, 62-89%) |  |
| Tafenoquine | Placebo | Dow, 2022, USA | RP | 45 | 41 | 0 | Tafenoquine administered orally as 2 × 100 mg tablets on study days 1, 2, 3, and 10 | Matching placebo | Day 28 Clinical Recovery (ITT) - Change in proportion unrecovered | Tx vs. Comparator:    %, p-value) | 13%, p=0.58 |  |
| Tafenoquine | Placebo | Dow, 2022, USA | RP | 45 | 41 | 0 | Tafenoquine administered orally as 2 × 100 mg tablets on study days 1, 2, 3, and 10 | Matching placebo | ITT time to clinical recovery | Tx vs comparator  N, mean (SE) vs. N, mean (SE)  *P* no covariates/P covariates | 4.4 (0.69) vs. 6.5 (0.84)  0.05/0.11 |  |

**Table 1b:** Effect of antivirals on time to recovery

| **Treatment Name** | **Comparator category** | **Study, country** | **Tx classification** | **Treatment (N)** | **Comparator (N)** | **Unique population** | **Tx dosage and duration** | **Type of comparator** | **Outcome Definition** | **Effect measure** | **Effect estimate** | **Notes** |
| --- | --- | --- | --- | --- | --- | --- | --- | --- | --- | --- | --- | --- |
| Favipiravir | Placebo | Golan, USA, Mexico, Brazil, 2023 | AV | 599 | 588 | 1 | 1801 mg twice daily on Day 1, followed by 800 mg twice daily on Days 2–10 | Matching placebo | Time to sustained clinical recovery | Tx vs comparator; Median time in days, (95% CI) | 7 (7-8) vs 7 (6-8) |  |
| Favipiravir | Placebo | Golan, USA, Mexico, Brazil, 2023 | AV | 599 | 588 | 1 | 1802 mg twice daily on Day 1, followed by 800 mg twice daily on Days 2–10 | Matching placebo | Time to sustained clinical recovery, by day 28 | Tx vs comparator, Proportion (%), p-value | 87.8% vs 87.3% |  |
| Leriterelvir | Placebo | Zhan, 2024, China | AV | 680 | 679 | 0 | Leritrelvir, 400 mg, three times a day orally for 5 days | Placebo, three times a day orally for 5 days | Time to sustained clinical recovery - hours | Tx vs comparator; Hours, Median (IQR)  Hazard ratio (95% CI)  p-value | 251.02 (188.95, 428.68) vs 271.33 (219.00, 529.63)  1.20 (1.07, 1.35)  p-value = 0.0022 |  |
| Leriterelvir | Placebo | Zhan, 2024, China | AV | 680 | 679 | 0 | Leritrelvir, 400 mg, three times a day orally for 5 days | Placebo, three times a day orally for 5 days | Time from the first dose to sustained clinical remission | Tx vs comparator; Hours, Median (IQR)  Hazard ratio (95% CI)  p-value | 243.33 (158.58, 368.78) vs 249.42 (171.43, 431.15)  1.16 (1.03, 1.30)  p-value = 0.011 |  |
| Leriterelvir | Placebo | Zhan, 2024, China | AV | 680 | 679 | 0 | Leritrelvir, 400 mg, three times a day orally for 5 days | Placebo, three times a day orally for 5 days | Participants in clinical recovery by date - Day 7 | Tx vs comparator; Day, N(%) (95% CI)  p-value | 20.88 (17.89, 24.13) vs. 15.76 (13.10, 18.72)  p-value = 0.014 |  |
| Leriterelvir | Placebo | Zhan, 2024, China | AV | 680 | 679 | 0 | Leritrelvir, 400 mg, three times a day orally for 5 days | Placebo, three times a day orally for 5 days | Participants in clinical recovery by date - Day 14 | Tx vs comparator; Day, N(%) (95% CI)  p-value | 66.18 (62.48, 69.73) vs 58.32 (54.51, 62.06)  p-value = 0.0029 |  |
| Leriterelvir | Placebo | Zhan, 2024, China | AV | 680 | 679 | 0 | Leritrelvir, 400 mg, three times a day orally for 5 days | Placebo, three times a day orally for 5 days | Participants in clinical recovery by date - Day 21 | Tx vs comparator; Day, N(%) (95% CI)  p-value | 79.12 (75.87, 82.11) vs 72.90 (69.39, 76.21)  p-value = 0.0073 |  |
| Leriterelvir | Placebo | Zhan, 2024, China | AV | 680 | 679 | 0 | Leritrelvir, 400 mg, three times a day orally for 5 days | Placebo, three times a day orally for 5 days | Participants in clinical recovery by date - Day 28 | Tx vs comparator; Day, N(%) (95% CI)  p-value | 83.68 (80.68, 86.38) vs 79.23 (75.99, 82.23)  p-value = 0.035 |  |
| Leriterelvir | Placebo | Zhan, 2024, China | AV | 680 | 679 | 0 | Leritrelvir, 400 mg, three times a day orally for 5 days | Placebo, three times a day orally for 5 days | Participants in clinical remission by date - Day 7 | Tx vs comparator; Day, N(%) (95% CI)  p-value | 29.12 (25.73, 32.69) vs   23.86 (20.70, 27.25)  p-value = 0.028 |  |
| Leriterelvir | Placebo | Zhan, 2024, China | AV | 680 | 679 | 0 | Leritrelvir, 400 mg, three times a day orally for 5 days | Placebo, three times a day orally for 5 days | Participants in clinical remission by date - Day 14 | Tx vs comparator; Day, N(%) (95% CI)  p-value | 70.44 (66.85, 73.85) vs 64.51 (60.78, 68.11)  p-value = 0.021 |  |
| Leriterelvir | Placebo | Zhan, 2024, China | AV | 680 | 679 | 0 | Leritrelvir, 400 mg, three times a day orally for 5 days | Placebo, three times a day orally for 5 days | Participants in clinical remission by date - Day 21 | Tx vs comparator; Day, N(%) (95% CI)  p-value | 82.94 (79.90, 85.69) vs 78.06 (74.75, 81.12)  p-value = 0.024 |  |
| Leriterelvir | Placebo | Zhan, 2024, China | AV | 680 | 679 | 0 | Leritrelvir, 400 mg, three times a day orally for 5 days | Placebo, three times a day orally for 5 days | Participants in clinical remission by date - Day 28 | Tx vs comparator; Day, N(%) (95% CI)  p-value | 86.91 (84.14, 89.36) vs 83.21 (80.18, 85.95)  p-value = 0.057 |  |
| Molnupiravir | Standard/Usual Care | Butler, UK, 2023 | AV | 12,774 | 12,934 | 1 | 800 mg molnupiravir orally twice daily for 5 days. | Standard of care | Early sustained recovery (recovery by day 14 sustained until day 28) | Estimated treatment OR (95% BCI) | 1.62 (1.53-1.72);p = >0.99 | Early sustained recovery |
| Molnupiravir | Standard/Usual Care | Butler, UK, 2023 | AV | 12,774 | 12,934 | 1 | 800 mg molnupiravir orally twice daily for 5 days. | Standard of care | Number of days until sustained recovery | Estimated treatment HR (95% BCI); Estimated benefit HR (95% BCI) | 1.24 (1.21-1.28); Estimated benefit = 3.5 (3.0 - 3.90); p = >0.99 | Days to sustained recovery |
| Molnupiravir + Standard/Usual Care | Standard/Usual Care | Butler, UK, 2023 | AV | 12,774 | 12,934 | 1 | 800 mg molnupiravir orally twice daily for 5 days. | Managing symptoms with antipyretics; However, patients at very high risk (ie, those with impaired immune systems or who are extremely clinically vulnerable— roughly 1·8 million people in the UK) are eligible to receive monoclonal antibodies (sotrovimab), intravenous antivirals (remdesivir), and oral antivirals (molnupiravir or nirmatrelvir–ritonavir) from specialist regional COVID-19 clinics (Ages 18-69 & 70-74; Tx n = 1,116 [9%]; Control: 1,111 [9%]) | Time to first self-reported recovery | Hazard Ratio (HR), BCI | 1·36 [ 1·32–1·40] |  |
| Molnupriavir | Placebo | Guan, 2023, UK | AV | 709 | 699 | 1 | 800 mg, oral molnupiravir, twice daily, for 5 days | 800 mg, oral placebo, twice daily, for 5 days | Sustained resolution/improvement by day 10 | N/A | Among the 5 distinctive COVID-19 symptoms, participants in the molnupiravir group were most likely to achieve sustained resolution/improvement by day 3, day 5 (end of treatment), and day 10 for all symptoms, except for cough on day 10 | Unique population - At-risk, unvaccinated, nonhospitalized adults |
| Molnupriavir + Standard/Usual Care | Standard/Usual Care | Sinha, 2022, India | AV | 608 | 610 | 1 | molnupiravir 800 mg of (4 × 200 mg capsules, administered orally every 12 hours for five days) plus + SOC | SoC | Time to clinical recovery | Tx vs comparator; Median time (IQR) | 6.0 days (6.00:7.00); vs 10 days ( 6.00:11.00), p < 0.0001 |  |
| SNG001 | Placebo | Francis, 2023, Britain | AV | 56 | 58 | 0 | Inhale SNG001, once daily via nebulizer, for 14 days | Placebo, once only via nebulizer, for 14 days | Time-to-first recovery >1 according to the World Health Organization Ordinal Scale of Clinical Improvement (WHO OSCI) | Hazard ratio, [95% CI; p-value<0.05 is considered statistically significant] | 0.84 (0.43, 1.65) p=0.62 |  |
| SNG001 | Placebo | Francis, 2023, Britain | AV | 56 | 58 | 0 | Inhale SNG001, once daily via nebulizer, for 14 days | Placebo, once only via nebulizer, for 14 days | Time-to-first improvement ≥1 according to the World Health Organization Ordinal Scale of Clinical Improvement (WHO OSCI) | Hazard ratio, [95% CI; p-value<0.05 is considered statistically significant] | 0.75 (0.40, 1.40) p=0.36 |  |

**Table 2a:** Effect of repurposed treatments on any admission to the emergency department

| **Treatment Name** | **Comparator category** | **Study, country** | **Tx classification** | **Treatment (N)** | **Comparator (N)** | **Unique population** | **Tx dosage and duration** | **Type of comparator** | **Outcome Definition** | **Effect measure** | **Effect estimate** | **Note** |
| --- | --- | --- | --- | --- | --- | --- | --- | --- | --- | --- | --- | --- |
| Acebilustat | Placebo | Levitt, 2023, US | RP | 60 | 60 | 0 | 100 mg of oral acebilustat, daily, for 28 days | 100 mg of oral placebo, daily, for 28 days | Emergency department visits between the treatment groups | Tx. vs Comparator; N   p-value | 1 vs 5  p-value = 0.21 |  |
| Budesonide | Standard/Usual Care | Ramakrishnan 2021, Oxfordshire, UK | RP | 73 | 73 | 0 | Budesonide dry powder inhaler at a dose of 400 µg per actuation (two puffs to be taken twice per day; total dose 1600 µg) until symptom resolution. | Usual care was supportive therapy | Emergency department  assessment or hospitalisation | Difference in proportion, 95% CI, p value | 0·123, 0·033–0·213; p=0·009 |  |
| Fluvoxamine | Placebo | Lenze 2020, St. Louis metropolitan area (Missouri and Illinois) | RP | Fluvoxamine (n = 80) | Placebo (n = 72) | 0 | 100 mg of fluvoxamine for 15 days | 100 mg of placebo for 15 days | 30-d post trial observation events (emergency department visit for chest pain (costochondritis COVID-19 sequela)) | Tx vs comparator; No of participants | 0 vs 1 |  |
| Fluvoxamine | Placebo | Reis 2022, Brazil | RP | 741 | 756 | 0 | fluvoxamine (Luvox, Abbott) at a dose of 100 mg twice a day for 10 days starting directly after randomisation (day 1). | Corresponding placebo starting directly after randomisation (day 1). | Emergency setting visit for at  least 6 h | OR 95% CI | 0·19 (0·08–0·41) | Unadjusted odds ratio |
| Ivermectin | Placebo | Lopez-Medina 2021, Columbia | RP | 200 | 198 | 0 | Ivermectin, 300 μg/kg of body weight per day for 5 days | Placebo 300 μg/kg of body weight per day for 5 days | Emergency department visits or telemedicine consultations, | Absolute difference; 95% CI | 1.24 (0.56 to 2.74) |  |
| Losartan | Placebo | Puskarich, 2021 | RP | 58 | 59 | 0 | Losartan 25mg, self-administered orally twice daily for 10 days for participants with eGFR >60 mL/min/1.73 m2, and once daily for those with eGFR 30–60 mL/min/1.73 m2. | 1:1 placebo | ED/clinic vist within 28 days of treatment start | difference in proportion, 95% confidence interval | -1.8 (-13.3, 9.4) |  |
| Metformin; Ivermectin; Fluvoxamine | Placebo + Control | Bramante 2022, United States | RP | Metformin = 663 , Ivermectin = 410 , Fluvoxamine = 334 | Metformin control= 660, Ivermectin control= 398, Fluvoxamine control = 327 | 0 | immediate-release metformin administered with an increase in dose over 6 days to 1500 mg per day for 14 days, ivermectin at a dose of 390 to 470 μg per kilogram per day for 3 days, and fluvoxamine at a dose of 50 mg twice daily for 14 days. | The metformin control group included the patients who had received placebo, fluvoxamine alone, or ivermectin alone. Patients in the ivermectin control group were randomly assigned to receive either placebo or metformin alone. The fluvoxamine and fluvoxamine control groups were constructed similarly. The control groups that were used in the comparisons with the active-drug groups included only concurrently randomly assigned patient | Emergency department visit; Metformin | Adjusted Odds Ratio (95% CI) | 0.58 (0.35-0.94) | Modified ITT population. Adjusted odds ratios and 95% confidence intervals are based on a logistic-regression model that was adjusted for baseline vaccination status and the receipt of other medications during the trial; multiple imputation was used with chained equations and predictive mean matching. |
| Metformin; Ivermectin; Fluvoxamine | Placebo + Control | Bramante 2022, United States | RP | Metformin = 663 , Ivermectin = 410 , Fluvoxamine = 335 | Metformin control= 660, Ivermectin control= 398, Fluvoxamine control = 328 | 0 | immediate-release metformin administered with an increase in dose over 6 days to 1500 mg per day for 14 days, ivermectin at a dose of 390 to 470 μg per kilogram per day for 3 days, and fluvoxamine at a dose of 50 mg twice daily for 14 days. | The metformin control group included the patients who had received placebo, fluvoxamine alone, or ivermectin alone. Patients in the ivermectin control group were randomly assigned to receive either placebo or metformin alone. The fluvoxamine and fluvoxamine control groups were constructed similarly. The control groups that were used in the comparisons with the active-drug groups included only concurrently randomly assigned patient | Emergency department visit; Ivermectin | Adjusted Odds Ratio (95% CI) | 1.39 (0.72-2.69) | Modified ITT population. Adjusted odds ratios and 95% confidence intervals are based on a logistic-regression model that was adjusted for baseline vaccination status and the receipt of other medications during the trial; multiple imputation was used with chained equations and predictive mean matching. |
| Metformin; Ivermectin; Fluvoxamine | Placebo + Control | Bramante 2022, United States | RP | Metformin = 663 , Ivermectin = 410 , Fluvoxamine = 336 | Metformin control= 660, Ivermectin control= 398, Fluvoxamine control = 329 | 0 | immediate-release metformin administered with an increase in dose over 6 days to 1500 mg per day for 14 days, ivermectin at a dose of 390 to 470 μg per kilogram per day for 3 days, and fluvoxamine at a dose of 50 mg twice daily for 14 days. | The metformin control group included the patients who had received placebo, fluvoxamine alone, or ivermectin alone. Patients in the ivermectin control group were randomly assigned to receive either placebo or metformin alone. The fluvoxamine and fluvoxamine control groups were constructed similarly. The control groups that were used in the comparisons with the active-drug groups included only concurrently randomly assigned patient | Emergency department visit; Fluvoxamine | Adjusted Odds Ratio (95% CI) | 1.17 (0.57-2.40) | Modified ITT population. Adjusted odds ratios and 95% confidence intervals are based on a logistic-regression model that was adjusted for baseline vaccination status and the receipt of other medications during the trial; multiple imputation was used with chained equations and predictive mean matching. |

**Table 2b:** Effect of antivirals on any admission to the emergency department

| **Treatment Name** | **Comparator category** | **Study, country** | **Tx classification** | **Treatment (N)** | **Comparator (N)** | **Unique population** | **Tx dosage and duration** | **Type of comparator** | **Outcome Definition** | **Effect measure** | **Effect estimate** | **Note** |
| --- | --- | --- | --- | --- | --- | --- | --- | --- | --- | --- | --- | --- |
| Favipiravir | Placebo | Holubar 2022, US | AV | 59 | 57 | 0 | favipiravir 1800 mg twice daily (BID) on day 1, then 800 mg BID on days 2–10. | Placebo | ED visits by day 28 | Tx vs comparator, %, p value | 10 vs 7; p=0.56 | Among the ITT population |
| Molnupiravir | Placebo | Johnson 2022, 107 sites in Africa, the Asia-Pacific region, Europe, Latin America, and North America | AV | 709 | 699 | 0 | Molnupiravir, 800 mg every 12 hours for 5 days | matching placebo every 12 hours for 5 days | COVID-19–related acute care visits through day 29 (MITT population). | % difference 95% CI | -3.4% (-6.3% to -0.5%) | Modified ITT |

**Table 3a:** Effect of repurposed treatments on any hospital admission and/or duration

| **Treatment Name** | **Comparator category** | **Study, country** | **Tx classification** | **Treatment (N)** | **Comparator (N)** | **Unique population** | **Tx dosage and duration** | **Type of comparator** | **Outcome Definition** | **Effect measure** | **Effect estimate** | **Note** |
| --- | --- | --- | --- | --- | --- | --- | --- | --- | --- | --- | --- | --- |
| Acebilustat | Placebo | Levitt, 2023, US | RP | 60 | 60 | 0 | 100 mg of oral acebilustat, daily, for 28 days | 100 mg of oral placebo, daily, for 28 days | Hospitalizations | Tx vs comparator; N | 1 vs 1 | Not quantitatively expressed; 1 subject in each arm |
| Amantadine | Placebo | Weis, 2023, Denmark | RP | 121 | 121 | 1 | Amantadine 100 mg twice daily for 5 days | Placebo twice daily for 5 days | Hospitalization during study duration | N vs N; Tx vs comparator | 5 vs 3; p=0.72 |  |
| Aspirin + placebo; 2.5mg Apixaban; 5mg Apixaban | Placebo | Connors 2021, US | RP | Aspirin (n=144),  Prophylactic Apixaban (n=135),  Therapeutic Apixaban (n=143) | Placebo (n=136) | 0 | 1. Aspirin (81 mg orally once daily) with matching placebo. 2. Prophylactic-dose apixaban (2.5 mg orally twice daily. 3. Therapeutic-dose apixaban (5 mg orally twice daily)  For 45 days | Placebo twice daily | Hospitalization for cardiopulmonary events. | N (%) | Aspirin = 0  Prophylactic Apixaban = 1 (0.7)  Therapeutic Apixaban = 2 (1.4)  Placebo = 1 (0.7) |  |
| Azithromycin | Placebo | Oldenburg, USA, 2021 | RP | 171 | 92 | 0 | Single oral 1.2g Azithromycin dose | Matching placebo | All-cause hospitalization or death through day 21 | Proportion of participants who are symptom free, difference (95% CI) | 4%; (−1% to 9%) P = .16). |  |
| Azithromycin | Placebo | Oldenburg, USA, 2022 | RP | 171 | 92 | 0 | Single oral 1.2g Azithromycin dose | Matching placebo | ED or urgent care visit through day 21 | Proportion of participants who are symptom free, difference (95% CI) | 12% (3% to 20%), p=0.01 |  |
| Azithromycin + Standard/Usual Care | Standard/Usual Care | Butler 2021, UK | RP | 186 | 236 | 1 | Oral Azithromycin 500 mg once daily for three days plus usual care | Usual care | Hospital admission or death  within 28 days of random assignment | Absolute benefit; 95% Bayseian credible interval | 1·6% (–3·1 to 6·2) | n value used is in SARS-CoV-2-positive analysis population for primary analysis.Estimated absolute benefit in percentage of hospitalisation or death derived from a Bayesian logistic regression model adjusted for age and comorbidity at baseline, with 95% Bayesian credible interval. A positive value favours azithromycin |
| Budesonide | Standard/Usual Care | Ramakrishnan. 2021, UK | RP | 71 | 69 | 0 | Budesonide dry powder inhaler (two puffs to be taken twice per day; total dose 1600 μg) | Usual care | COVID-19-related urgent care visits, including emergency department assessment or hospitalisation | Tx vs comparator; N (%) vs. N(%)  Difference in proportions, 95% CI, p-value | 2 (3%) vs 11 (15%) | ITT |
| Budesonide | Standard/Usual Care | Yu, UK, 2021 | RP | 833 | 1126 | 1 | 800 μg Budesonide twice daily for 14 days | Usual care + other inventions | Hospital admission within 28 days | Odds Ratio (OR), 95% BCI | 0.75 ( 0.55 to 1.03) |  |
| Camostat | Placebo | Jilg, 2023, USA | RP | 109 | 107 | 0 | 200 mg oral camostat (2 tablets, 100 mg each) every 6 hours for 7 days | 200 mg oral placebo (2 tablets, 100 mg each) every 6 hours for 7 days | Hospitalizations through day 28 | Camostat vs. Placebo:   n (%) vs. n (%)  RR [95% CI] | 6 (5.6%) vs. 5 (4.7%) 1.2 [ 0.4, 3.8] |  |
| Ciclesonide | Placebo | Ezer 2021, Canada | RP | 105 | 98 | 1 | inhaled ciclesonide (600 μg twice daily) and intranasal ciclesonide (200 μg daily) for 14 days. | metered dose inhaler and nasal saline placebos for 14 days | Admission to hospital: day 14 | Adjusted risk difference‡, % (95% CI), Tx vs comparator N% | **2.3 (−3.0 to 7.6), 6 (6)** vs **3 (3)** | ‡Adjusted for stratification on sex |
| Colchicine | Placebo | Tardif 2021, Brazil, Canada, Greece, South Africa, Spain, and the USA | RP | 2235 | 2253 | 0 | 0·5 mg colchicine orally administered twice per day for the first 3 days and then  once per day for 27 days thereafter | Matching placebo | Hospitalisation for COVID-19 | Odds ratio  (95% CI) | 0·79 (0·60–1·03) |  |
| Colchicine | Standard/Usual Care | Eikelboom 2022, Brazil, Canada, Colombia, Ecuador, Egypt, India, Nepal, Pakistan, Phillippines, Russia, South Africa, United Arab Emirates | RP | 1939 | 1942 | 0 | oral colchicine 0·6 mg twice daily for 3 days and then 0.6 mg once daily for 25 days or control | usual care as established by local site investigator | Any hospitalization | HR (95% CI) | 1·02 (0·71–1·45) |  |
| Colchicine | Standard/Usual Care | Eikelboom 2022, Brazil, Canada, Colombia, Ecuador, Egypt, India, Nepal, Pakistan, Phillippines, Russia, South Africa, United Arab Emirates | RP | 1939 | 1942 | 0 | oral colchicine 0·6 mg twice daily for 3 days and then 0.6 mg once daily for 25 days or control | usual care as established by local site investigator | Hospitalization or death | HR (95% CI) | 1.02 (0.72-1.43) |  |
| Colchicine | Standard/Usual Care | Eikelboom 2022, Brazil, Canada, Colombia, Ecuador, Egypt, India, Nepal, Pakistan, Phillippines, Russia, South Africa, United Arab Emirates | RP | 1939 | 1942 | 0 | oral colchicine 0·6 mg twice daily for 3 days and then 0.6 mg once daily for 25 days or control | usual care as established by local site investigator | Hospitalization or respiratory death | HR (95% CI) | 1.00 (0.71-1.41) |  |
| Domperidone + Standard/Usual Care | Placebo | Basalo, 2023, Spain | RP | 87 | 86 | 0 | Three daily doses of 10 mg (30 mg/day) of domperidone for 7 days | Placebo, three daily doses, for 7 days | Hospitalized due to COVID-19 disease | N vs N | 0 vs 0 | No patients needed to be hospitalized |
| Doxycycline + Standard/Usual Care | Standard/Usual Care | Butler 2021, UK | RP | 780 | 644 | 0 | usual care plus oral doxycycline (200 mg on day 1, followed by 100 mg once daily for the following 6 days) | usual care only: largely supportive. | Duration of hospital admission in days | Quantile regression (95% CI) | –2·40 (–5·40 to 0·52) | n value from concurrent randomisation analysis population. Quantile regression adjusted for age, comorbidity, duration of illness, and eligibility for doxycycline at baseline. |
| Febuxostat | Hydroxychloroquine | Davoodi 2020, Iran | RP | 29 | 25 |  | Febuxostat = one tablet of FBX 80 mg per day for 5 days | Hydroxyxhloroquine = one tablet of HCQ 200 mg twice daily for 5 days | the rate of hospitalization | Tx vs comparator; N(%) | 3 (10.3), vs 3 (12.5) |  |
| Fluticasone Furoate | Placebo | Boulware, USA, 2023 | RP | 656 | 621 | 1 | 200 μg once daily for 14 days | Matching placebo once daily for 14 days | Hospitalization or death through day 28 | N (%) vs. N(%) | 3(0.5) vs 3(0.5) | Unique patient population: 30 years or older |
| Fluticasone Furoate | Placebo | Boulware, USA, 2023 | RP | 656 | 621 | 1 | 200 μg once daily for 14 days | Matching placebo once daily for 14 days | Composite of hospitalization, urgent-care  visit, emergency department visit,  or death | HR (95% Credible interval) | 1.9 (0.8 to 3.5), p=0.04 | Unique patient population: 30 years or older |
| Fluvoxamine | Placebo | Stewart, 2023, USA | RP | 589 | 586 | 1 | 50 mg (one 50-mg tablet) Fluvoxamine twice daily for 1 day, followed by 100 mg (two 50-mg tablets) | Matching placebo twice daily for 1 day (two 50-mg tablets) or matching placebo twice daily for 12 days, for a total 13-day course | Hospitalization or death through day 28 | N (%) vs. N(%)  HR (adjusted estimate) [95% CI] | 1 (0.17%) vs. 2 (0.34%)   0.51 (0.05 to 5.64) | Unique patient population: 30 years or older |
| Fluvoxamine | Placebo | McCarthy, USA, 2023 | RP | 674 | 614 | 1 | Fluvoxamine (50 mg twice daily) for 10 days | Placebo | Composite outcome of hospitalization, urgent care visit, ED visit, or death through Day 28 | Tx vs comparator, N vs N, Hazard Ratio (HR, 95% CI) | 26 (3.9%) vs 23 (3.8%); 1.1 (0.5 to 1.8), p=0.35 |  |
| Fluvoxamine | Placebo | Lenze 2020, St. Louis metropolitan area (Missouri and Illinois) | RP | Fluvoxamine (n = 80) | Placebo (n = 72) | 0 | 100 mg of fluvoxamine for 15 days | 100 mg of placebo for 15 days | Oxygen saturation less than 92% plus supplemental oxygen needed and hospitalization related to dyspnea or hypoxia. | Asbsolute difference, 95 CI, and Tx vs comparator N (%) | ,−4.2 (−13.2 to 2.0), P value = 0.07; 0 vs 3 (4.2) |  |
| Fluvoxamine | Placebo | Lenze 2020, St. Louis metropolitan area (Missouri and Illinois) | RP | Fluvoxamine (n = 80) | Placebo (n = 72) | 0 | 100 mg of fluvoxamine for 15 days | 100 mg of placebo for 15 days | 30-d post trial observation events (hospitalization for post-COVID headache) | Tx vs comparator; No of participants | 1 vs 0 |  |
| Fluvoxamine | Standard/Usual Care | Wannigama 2024, Thailand | RP | 162 | 336 | 0 | The fluvoxamine-only arm received fluvoxamine (immediate release) 50 mg orally, one tablet in the morning and one tablet at bedtime for the first two days, then escalated to one 50 mg one tablet in the morning and two tablets before bedtime for days 3 through 12, ending with one 50 mg one tablet in the morning and one tablet at bedtime for days 13 and 14 | Standard care (based on COVID-19 treatment guidelines at the time of the trial) | Hospitalized, requiring non-invasive mechanical ventilation or high flow oxyegenation, low flow supplemental oxygen, or not requiring supplemental oxygen, and requiring ongoing medical care at day 9 | Tx vs comparator; N (%) | 0 vs 208 (61.8); p<0.0001 |  |
| Fluvoxamine | Standard/Usual Care | Wannigama 2024, Thailand | RP | 162 | 336 | 0 | The fluvoxamine-only arm received fluvoxamine (immediate release) 50 mg orally, one tablet in the morning and one tablet at bedtime for the first two days, then escalated to one 50 mg one tablet in the morning and two tablets before bedtime for days 3 through 12, ending with one 50 mg one tablet in the morning and one tablet at bedtime for days 13 and 15 | Standard care (based on COVID-19 treatment guidelines at the time of the trial) | Hospitalized, requiring non-invasive mechanical ventilation or high flow oxyegenation, low flow supplemental oxygen, or not requiring supplemental oxygen, and requiring ongoing medical care at day 14 | Tx vs comparator; N (%) | 0 vs 44 (40.3), p<0.0001 |  |
| Fluvoxamine | Standard/Usual Care | Wannigama 2024, Thailand | RP | 162 | 336 | 0 | The fluvoxamine-only arm received fluvoxamine (immediate release) 50 mg orally, one tablet in the morning and one tablet at bedtime for the first two days, then escalated to one 50 mg one tablet in the morning and two tablets before bedtime for days 3 through 12, ending with one 50 mg one tablet in the morning and one tablet at bedtime for days 13 and 16 | Standard care (based on COVID-19 treatment guidelines at the time of the trial) | Hospitalized, requiring non-invasive mechanical ventilation or high flow oxyegenation, low flow supplemental oxygen, or not requiring supplemental oxygen, and requiring ongoing medical care at day 28 | Tx vs comparator; N (%) | 9 (5.6) vs 37 (66.1), p<0.0001 |  |
| Fluvoxamine | Placebo | Reis 2022, Brazil | RP | 741 | 756 | 0 | fluvoxamine (Luvox, Abbott) at a dose of 100 mg twice a day for 10 days starting directly after randomisation (day 1). | Corresponding placebo starting directly after randomisation | All-cause hospitalisation | OR 95% CI | 0·76 (0·58–1·04) | Unadjusted odds ratio |
| Fluvoxamine | Placebo | Reis 2022, Brazil | RP | 741 | 756 | 0 | fluvoxamine (Luvox, Abbott) at a dose of 100 mg twice a day for 10 days starting directly after randomisation (day 1). | Corresponding placebo starting directly after randomisation | Period of hospitalisation | Linear regression estimate; 95% CI | 1·23 (0·99–1·53) | Exponentiated unadjusted estimates from a log-transformed linear regression. |
| Fluvoxamine | Placebo | Reis 2022, Brazil | RP | 741 | 756 | 0 | fluvoxamine (Luvox, Abbott) at a dose of 100 mg twice a day for 10 days starting directly after randomisation (day 1). | Corresponding placebo starting directly after randomisation (day 1). | Hospitalised for COVID | OR 95% CI | 0·77 (0·55–1·05) | Unadjusted odds ratio |
| Fluvoxamine + Bromhexine | Standard/Usual Care | Wannigama 2024, Thailand | RP | 178 | 336 | 0 | The fluvoxamine-only arm received fluvoxamine (immediate release) 50 mg orally, one tablet in the morning and one tablet at bedtime for the first two days, then escalated to one 50 mg one tablet in the morning and two tablets before bedtime for days 3 through 12, ending with one 50 mg one tablet in the morning and one tablet at bedtime for days 13 and 14. Plus one 8 mg bromhexine tablet twice daily for 10 days | Standard care (based on COVID-19 treatment guidelines at the time of the trial) | Hospitalized, requiring non-invasive mechanical ventilation or high flow oxyegenation, low flow supplemental oxygen, or not requiring supplemental oxygen, and requiring ongoing medical care at day 9 | Tx vs comparator; N (%), p-value | 0 vs 208 (61.8); p<0.0001 |  |
| Fluvoxamine + Bromhexine | Standard/Usual Care | Wannigama 2024, Thailand | RP | 178 | 336 | 0 | The fluvoxamine-only arm received fluvoxamine (immediate release) 50 mg orally, one tablet in the morning and one tablet at bedtime for the first two days, then escalated to one 50 mg one tablet in the morning and two tablets before bedtime for days 3 through 12, ending with one 50 mg one tablet in the morning and one tablet at bedtime for days 13 and 14. Plus one 8 mg bromhexine tablet twice daily for 10 days | Standard care (based on COVID-19 treatment guidelines at the time of the trial) | Hospitalized, requiring non-invasive mechanical ventilation or high flow oxyegenation, low flow supplemental oxygen, or not requiring supplemental oxygen, and requiring ongoing medical care at day 14 | Tx vs comparator; N (%), p-value | 0 vs 44 (40.3), p<0.0001 |  |
| Fluvoxamine + Bromhexine | Standard/Usual Care | Wannigama 2024, Thailand | RP | 178 | 336 | 0 | The fluvoxamine-only arm received fluvoxamine (immediate release) 50 mg orally, one tablet in the morning and one tablet at bedtime for the first two days, then escalated to one 50 mg one tablet in the morning and two tablets before bedtime for days 3 through 12, ending with one 50 mg one tablet in the morning and one tablet at bedtime for days 13 and 14. Plus one 8 mg bromhexine tablet twice daily for 10 days | Standard care (based on COVID-19 treatment guidelines at the time of the trial) | Hospitalized, requiring non-invasive mechanical ventilation or high flow oxyegenation, low flow supplemental oxygen, or not requiring supplemental oxygen, and requiring ongoing medical care at day 28 | Tx vs comparator; N (%), p-value | 0 vs 37 (66.1), p<0.0001 |  |
| Fluvoxamine + Budesonide | Placebo | Reis, Brazil, 2023 | RP | 738 | 738 | 0 | fluvoxamine (100 mg twice daily for 10 days) plus inhaled budesonide (800 mcg twice daily for 10 days) | matching placebos | Composite of hospitalization, emergency care visits, or suspected complications | Relative risk (RR); Risk difference (95% CrI) | 0.50 [0.25 to 0.92]; risk difference, 0.019 [ 0.017 to 0.020]); | |
| Fluvoxamine + Budesonide | Placebo | Reis, Brazil, 2023 | RP | 738 | 738 | 0 | fluvoxamine (100 mg twice daily for 10 days) plus inhaled budesonide (800 mcg twice daily for 10 days) | matching placebos | Hospitalization | Tx vs comparator; Relative risk (RR), 95% CrI | 0.89 (0.33–2.35) |  |
| Fluvoxamine + Budesonide | Placebo | Reis, Brazil, 2023 | RP | 738 | 738 | 0 | fluvoxamine (100 mg twice daily for 10 days) plus inhaled budesonide (800 mcg twice daily for 10 days) | matching placebos | Days to hospitalization | HR (95% CrI) | 0.86 (0.31 -2.38) |  |
| Fluvoxamine + Budesonide | Placebo | Reis, Brazil, 2023 | RP | 738 | 738 | 0 | fluvoxamine (100 mg twice daily for 10 days) plus inhaled budesonide (800 mcg twice daily for 10 days) | matching placebos | Any ED visit | RR (95% CrI) | 0.94 (0.72–1.22) |  |
| Fluvoxamine + Cyproheptadine | Standard/Usual Care | Wannigama 2024, Thailand | RP | 147 | 336 | 0 | The fluvoxamine-only arm received fluvoxamine (immediate release) 50 mg orally, one tablet in the morning and one tablet at bedtime for the first two days, then escalated to one 50 mg one tablet in the morning and two tablets before bedtime for days 3 through 12, ending with one 50 mg one tablet in the morning and one tablet at bedtime for days 13 and 14. Plus one 4 mg cyproheptadine tablet, three times a day for 14 days | Standard care (based on COVID-19 treatment guidelines at the time of the trial) | Hospitalized, requiring non-invasive mechanical ventilation or high flow oxyegenation, low flow supplemental oxygen, or not requiring supplemental oxygen, and requiring ongoing medical care at day 9 | Tx vs comparator; N (%), p-value | 0 vs 208 (61.8); p<0.0001 |  |
| Fluvoxamine + Cyproheptadine | Standard/Usual Care | Wannigama 2024, Thailand | RP | 147 | 336 | 0 | The fluvoxamine-only arm received fluvoxamine (immediate release) 50 mg orally, one tablet in the morning and one tablet at bedtime for the first two days, then escalated to one 50 mg one tablet in the morning and two tablets before bedtime for days 3 through 12, ending with one 50 mg one tablet in the morning and one tablet at bedtime for days 13 and 14. Plus one 4 mg cyproheptadine tablet, three times a day for 14 days | Standard care (based on COVID-19 treatment guidelines at the time of the trial) | Hospitalized, requiring non-invasive mechanical ventilation or high flow oxyegenation, low flow supplemental oxygen, or not requiring supplemental oxygen, and requiring ongoing medical care at day 14 | Tx vs comparator; N (%), p-value | 0 vs 44 (40.3), p<0.0001 |  |
| Fluvoxamine + Cyproheptadine | Standard/Usual Care | Wannigama 2024, Thailand | RP | 147 | 336 | 0 | The fluvoxamine-only arm received fluvoxamine (immediate release) 50 mg orally, one tablet in the morning and one tablet at bedtime for the first two days, then escalated to one 50 mg one tablet in the morning and two tablets before bedtime for days 3 through 12, ending with one 50 mg one tablet in the morning and one tablet at bedtime for days 13 and 14. Plus one 4 mg cyproheptadine tablet, three times a day for 14 days | Standard care (based on COVID-19 treatment guidelines at the time of the trial) | Hospitalized, requiring non-invasive mechanical ventilation or high flow oxyegenation, low flow supplemental oxygen, or not requiring supplemental oxygen, and requiring ongoing medical care at day 28 | Tx vs comparator; N (%), p-value | 0 vs 37 (66.1), p<0.0001 |  |
| Fluvoxamine + Favipiravir; Fluvoxamine + Favipiravir + Dexamethasone | Favipiravir | Siripongboonsitti, 2023, Thailand | RP | 134 | 132 | 1 | 50 mg twice daily of fluvoxamine for 10 days and favipiravir; patients with moderate illness also received 6 mg per day of dexamethasone for 10 days | Each group received 3600 mg of FPV loading dose on day 0 and 1600 mg per day of FPV from days 1-4 in mild COVID-19 and days 1-9 in moderate COVID-19. In a patient with obesity, the FPV loading dose was 4800 mg on day 0, then 2000 mg per day of FPV on days later | Hospitalization in 14 days, mild illness | Tx vs comparator, N(%), p value | 2 (1.52) vs 0, p=0.245 |  |
| Fluvoxamine + Favipiravir; Fluvoxamine + Favipiravir + Dexamethasone | Favipiravir | Siripongboonsitti, 2023, Thailand | RP | 134 | 132 | 1 | 50 mg twice daily of fluvoxamine for 10 days and favipiravir; patients with moderate illness also received 6 mg per day of dexamethasone for 10 days | Each group received 3600 mg of FPV loading dose on day 0 and 1600 mg per day of FPV from days 1-4 in mild COVID-19 and days 1-9 in moderate COVID-19. In a patient with obesity, the FPV loading dose was 4800 mg on day 0, then 2000 mg per day of FPV on days later | Hospitalization in 14 days, moderate illness | Tx vs comparator, N(%), p value | 4 (13.33) vs 5 (16.13), p=1.0 |  |
| Fluvoxamine + Favipiravir; Fluvoxamine + Favipiravir + Dexamethasone | Favipiravir | Siripongboonsitti, 2023, Thailand | RP | 134 | 132 | 1 | 50 mg twice daily of fluvoxamine for 10 days and favipiravir; patients with moderate illness also received 6 mg per day of dexamethasone for 10 days | Each group received 3600 mg of FPV loading dose on day 0 and 1600 mg per day of FPV from days 1-4 in mild COVID-19 and days 1-9 in moderate COVID-19. In a patient with obesity, the FPV loading dose was 4800 mg on day 0, then 2000 mg per day of FPV on days later | Hospitalization in 28 days, mild illness | Tx vs comparator, N(%), p value | 2 (1.52) vs 0, p=0.245 |  |
| Fluvoxamine + Favipiravir; Fluvoxamine + Favipiravir + Dexamethasone | Favipiravir | Siripongboonsitti, 2023, Thailand | RP | 134 | 132 | 1 | 50 mg twice daily of fluvoxamine for 10 days and favipiravir; patients with moderate illness also received 6 mg per day of dexamethasone for 10 days | Each group received 3600 mg of FPV loading dose on day 0 and 1600 mg per day of FPV from days 1-4 in mild COVID-19 and days 1-9 in moderate COVID-19. In a patient with obesity, the FPV loading dose was 4800 mg on day 0, then 2000 mg per day of FPV on days later | Hospitalization in 28 days, moderate illness | Tx vs comparator, N(%), p value | 4 (13.33) vs 5 (16.13), p=1.0 |  |
| Fluvoxamine + Standard/Usual Care | Placebo + Standard/Usual Care | Ibrahim, 2023, Egypt | RP | 37; moderate: 51 | 35; moderate: 39 | 0 | standard therapy plus fluvoxamine at a dose of 100 mg twice daily for 10 days. | favipiravir (nonspecific antiviral drug) plus nonspecific antibiotics as a standard regimen in our study according to local recommendations | clinical deterioration (desaturation to <93% and/or need for hospitalization due to COVID-related illness), hospitalization among mild cases | Tx vs comparator; N (%) vs. N(%), p-value | 1 (2.7%) vs 3 (8.6%) p = 0.277 | Mild: non-pneumonic; Moderate: pneumonic |
| Fluvoxamine + Standard/Usual Care | Placebo + Standard/Usual Care | Ibrahim, 2023, Egypt | RP | 37; moderate: 51 | 35; moderate: 39 | 0 | standard therapy plus fluvoxamine at a dose of 100 mg twice daily for 10 days. | favipiravir (nonspecific antiviral drug) plus nonspecific antibiotics as a standard regimen in our study according to local recommendations | clinical deterioration (desaturation to <93% and/or need for hospitalization due to COVID-related illness), hospitalization among moderate cases | Tx vs comparator; N (%) vs. N(%) , p-value; OR | 6 (11.8) vs 11 (28.2), p=0.048; OR 2.94 | Mild: non-pneumonic; Moderate: pneumonic |
| Halofuginone | Placebo | Tomazini, 2024, Brazil | RP | Group A: 50  Group B: 52 | Group C: 51 | 0 | Group A:  Halofuginone, 0.5mg, orally once daily for 10 days  Group B:  Halofuginone, 1mg, orally once daily for 10 days | Group C:  Placebo, orally once daily for 10 days | Hospital admissions within 28 days | Group A , Group B, Group C; N (%) | 0(0%)   2(3.8%)   0(0%) |  |
| Hydroxychloroquine | Placebo | Spivak, 2023, USA | RP | 185 | 182 | 0 | Participants randomized to the HCQ arm received 400 mg HCQ by mouth twice daily for 1 day and then 200 mg by mouth twice daily for 4 days. | Matching placebo | Hospitalization within 14 days | n (%) vs. N (%)   p-value | 7 (4.6%) vs. 4 (2.6%)  p=0.36 |  |
| Hydroxychloroquine | Placebo | Spivak, 2023, USA | RP | 185 | 182 | 0 | Participants randomized to the HCQ arm received 400 mg HCQ by mouth twice daily for 1 day and then 200 mg by mouth twice daily for 4 days. | Matching placebo | Hospitalization within 28 days | n (%) vs. N (%)   p-value | 7 (4.6%) vs. 4 (2.7%)  p=0.541 |  |
| Hydroxychloroquine | Placebo | Skipper, 2020, USA and Canada | RP | 244 | 247 | 0 | Hydroxychloroquine was prescribed at 800 mg (4 tablets) once, then 600 mg (3 tablets) 6 to 8 hours later, then 600 mg (3 tablets) once daily for 4 more days (5 days in total) | Matching placebo | The incidence of hospitalization or death | Tx. vs comparator; N vs N; p-value | 4 vs.10  p=0.29 | (2 were unrelated to COVID-19) |
| Hydroxychloroquine | Standard/Usual Care | Mitja 2021, Spain | RP | 136 | 157 | 0 | HCQ - Dolquine® 800 mg on day 1, followed by 400 mg once daily for six days) | Usual care | Hospitalization not requiring mechanical ventilation | Tx vs comparator; Events (%) | 8 (5.9) vs 11 (7.1) |  |
| Hydroxychloroquine | Placebo | Avezum, Brazil, 2022 | RP | 689 | 683 | 1 | hydroxychloroquine 400 mg twice a day in the first day, 400 mg once daily thereafter for a total of seven days | Placebo twice daily in the first day, then once daily thereafter for a total of seven days | Risk of hospitalization due to COVID-19 at 30 days | Risk Ratio (RR) | 0.77 [ 0.52–1.12], p = 0.16 |  |
| Hydroxychloroquine | Placebo | Schwartz 2021 | RP | 111 | 37 | 0 | hydroxychloroquine dose was 800 mg orally in divided  doses on day 1 followed by 200 mg twice daily for 4 days | identical matching placebo (12 tablets over 5 days) | Hospitalization within 30 d | Tx vs comparator; N% | 4 (3.6) vs 0 |  |
| Hydroxychloroquine + Azithromycin | Placebo | Rodrigues, Brazil, 2021 | RP | 42 | 42 | 1 | two 200 mg HCQ capsules twice a day (bid) for a total course of 7 days, d one 500 mg AZT capsule taken on day 1, followed by one 250 mg AZT capsule daily for the next 4 days | Placebo | Rate of hospitalization | Proportion of patients hospitalized | Tx: 1 (2.4%) |  |
| Hydroxychloroquine + Azithromycin; Hydroxychloroquine | Placebo | Johnston, USA, 2021 | RP | HCQ + azithromycin: 77; HCQ - azithromycin: 71 | 83 | 0 | hydroxychloroquine (HCQ) (400 mg BID x1day, followed by 200 mg BID x9days) with or without azithromycin (AZ) (500 mg, then 250 mg daily x4days) | placebo-equivalent (ascorbic acid (HCQ) and folic acid (AZ)) | Hospitalization or progression to lower respiratory tract infection (LRTI) or death by Day 28 | Tx vs comparator; Proportion hospitalized | 5.4% vs 4.7%) | Co-morbidities were summarized in text as all individuals randomized into the high-risk cohort across each of the three arms; no individual arm breakdown provided |
| Hydroxycloroquine | Placebo | Gonzalez, 2023, Spain | RP | 55 | 57 | 1 | HCQ (400 mg/day for 3 days, followed by 200 mg/day for 11 days) | placebo (2 tablets for 3 days, followed by one tablet for 11 days | Hospitalizations due to severe COVID-19 | Tx vs comparator, N vs N | 2 vs 2 | Unique patient population: Pregnant and postpartum women |
| Ivermectin | Placebo | Angkasekwinai, Thailand, 2022 | RP | 259 | 277 | 1 | ivermectin (400–600 µg/kg/d) once daily for 3 days | Placebo once daily for 3 days | Proportion hospitalized within 14 days | Rate of hospitalization | 8 (3.4% vs 4 (1.9%), p=0.386 |  |
| Ivermectin | Placebo | Reis 2022, Brazil | RP | 679 | 679 | 0 | Ivermectin 400 microgram/kg for 3 days, once per day | placebos that were used in the trial involved regimens of 1, 3, 10, or 14 days in duration, according to the various comparator groups in the trial at the time of randomization | Median no. of days of hospitalization | Tx vs comparator; Days (IQR), RR (95% CI) | 5 (3 to 7) vs 5 (3 to 8)  1.00 (0.80 to 1.25) |  |
| Ivermectin | Placebo | Lopez-Medina 2021, Columbia | RP | 200 | 198 | 0 | Ivermectin, 300 μg/kg of body weight per day for 5 days | Placebo 300 μg/kg of body weight per day for 5 days | Hospitalized, not requiring supplemental oxygen | Tx vs comparator; N (%) | 1 (0.5) vs 1 (0.5) |  |
| Ivermectin | Placebo | Reis 2022, Brazil | RP | 679 | 679 | 0 | Ivermectin 400 microgram/kg for 3 days, once per day | placebos that were used in the trial involved regimens of 1, 3, 10, or 14 days in duration, according to the various comparator groups in the trial at the time of randomization | Hospitalization due to COVID-19 wihtin 28 days after randomization or ED visit within 28 days | RR, 95% CI | 0.90 (0.70-1.16) |  |
| Ivermectin | Placebo | Lopez-Medina 2021, Columbia | RP | 200 | 198 | 0 | Ivermectin, 300 μg/kg of body weight per day for 5 days | Placebo 300 μg/kg of body weight per day for 5 days | Hospitalized, requiring supplemental oxygen | Tx vs comparator; N (%) | 1 (0.5) vs 1 (0.5) | Not high-flow nasal oxygen nor mechanical ventilation. |
| Ivermectin | Placebo | Naggie, USA, 2022 | RP | 817 | 774 | 1 | 400 μg/kg Ivermectin (n = 817), daily for 3 days | Placebo | Hospitalization or death through day 28 | Hazard ratio (HR), 95% CI | 1.1 [ 0.4-2.6]). |  |
| Ivermectin | Placebo | Naggie, USA, 2022 | RP | 817 | 774 | 1 | 400 μg/kg Ivermectin (n = 817), daily for 3 days | Placebo | Hospitalization, urgent care, ED visit, or death through day 28 | Hazard ratio (HR), 95% CI | 1.2 (0.6 to 1.8) |  |
| Ivermectin | Placebo; Fluvoxamine | Naggie, 2023, US | RP | 708 | 724 | 0 | dosing schedule was based on weight ranges as follows: those weighing 35 to 52 kg received a 21-mg daily dose ivermectin; 53 to 69 kg, 28-mg daily dose; 70 to 89 kg, 42-mg daily dose; 90 to 109 kg, 49-mg daily dose; 110 to 129 kg, 56-mg daily dose; and more than 129 kg, 70-mg daily dose | placebo, fluvoxamine 50mg twice daily | Hospitalization or death through day 28 | Hazard ratio (HR), 95% CI | 2.51 (0.49 to 12.96) |  |
| Ivermectin | Placebo; Fluvoxamine | Naggie, 2023, US | RP | 708 | 724 | 0 | dosing schedule was based on weight ranges as follows: those weighing 35 to 52 kg received a 21-mg daily dose ivermectin; 53 to 69 kg, 28-mg daily dose; 70 to 89 kg, 42-mg daily dose; 90 to 109 kg, 49-mg daily dose; 110 to 129 kg, 56-mg daily dose; and more than 129 kg, 70-mg daily dose | placebo, fluvoxamine 50mg twice daily | Hospitalization, urgent care, ED visit, or death through day 28 | Hazard ratio (HR), 95% CrI |  |  |
| Ivermectin | Placebo | Mirahmadizadeh 2022, Iran | RP | Ivermectin (S) = 130, Ivermectin (D) = 131 | Placebo = 130 | 0 | Single dose of ivermectin (3 mg tablet, cumulative dose of 12 mg, Stat. placebo tablets, at the second day) and double-dose ivermectin (3 mg tablet for 2 days, cumulative dose of 24 mg) at the day of RT-PCR-positive testing (enrolment day) and the next day. | Placebo | the proportion of subjects who required hospitalization up to 28 days of follow-up | Absolute difference 95% CI | -3.9 [-9.8, 2.2] | Ivermectin (Single Dose =S) Ivermectin (Double dose = D) |
| Ivermectin + Standard/Usual Care | Standard/Usual Care | Hayward, 2024, UK | RP | 2250 | 1869 | 0 | Participants were randomised to usual care plus ivermectin tablets (target 300–400 μg/kg per dose, once daily for 3 days) or usual care plus other interventions | Usual care | Hospitalization/death at 28 days | Odds ratio [95% CI]  Estimated difference in hospitalization rate   p-value | 1·02 (0·63 to 1·62)  0% [−1·0% to 0·6%]  p =0.472 | NOTE: Based on "Concurrently and eligible randomized population" |
| Ivermectin + Standard/Usual Care | Placebo + Standard/Usual Care | Vallejos 2021, Argentina | RP | 250 | 251 | 1 | Ivermectin plus standard of care (SOC) from randomization to the End Of Study (EOS) visit.  X tablets of 6 mg each at inclusion and another X tablets of 6 mg each 24 h after the first dose.  (The dose of ivermectin used was the approved dose in Argentina for the treatment of other diseases, such as parasitic diseases, and it was staggered according to weight.) | Placebo plus Standard of care (SOC)   (The SOC was in accordance with the recommendations of the Argentine Ministry of Health) | defined as a stay of at least 24 h in a health institution, in any of its services, at any point from randomization until the end of study visit. | Odds Ratio (95% CI) | 0.65 (0.32–1.31);  p = 0.227 | Estimated odds ratio in the Ivermectin arm between 0.3 and 0.5 with the aim of including a total of 500 patients (250 patients in each group) |
| Leflunomide + Standard/Usual Care | Standard/Usual Care | Pan, 2023, China | RP | 27 | 30 | 0 | leflunomide (50 mg orally every 12 h for three consecutive doses, then 20 mg once daily for 3 days, for a total of 5 days) + symptomtic treatment | symptomatic treatment, e.g., antipyretics, analgesics, antiemetic or antidiarrheal medicines, traditional Chinese medicine, supportive therapy, or supplemental oxygen as necessary | Hospitalization | Tx vs comparator; N vs N | 8 (29.6) vs 13 (43.3) p=0.426 |  |
| Levamisole | Standard/Usual Care | Asgardoon, 2022, Iran | RP | 185 | 180 | 0 | On days 1 to 10, Levamisole was administered orally at a dosage of 50 mg. | Standard care | Time between symptoms onset and hospitalization (days) | Tx vs comparator; Median (IQR)  p-value | 7.0 (7.0-7.0) vs. 9.5 (8.0-9.5)  p=0.102 |  |
| Losartan | Placebo | Puskarich, 2021 | RP | 58 | 59 | 0 | Losartan 25mg, self-administered orally twice daily for 10 days for participants with eGFR >60 mL/min/1.73 m2, and once daily for those with eGFR 30–60 mL/min/1.73 m2. | 1:1 placebo | hospital admission order to the hospital from the primary clinical team within 15 days of treatment start | difference in proportion, 95% confidence interval | -3.5 (-13.2, 4.8) |  |
| Losartan | Placebo | Puskarich, 2021 | RP | 58 | 59 | 0 | Losartan 25mg, self-administered orally twice daily for 10 days for participants with eGFR >60 mL/min/1.73 m2, and once daily for those with eGFR 30–60 mL/min/1.73 m2. | 1:1 placebo | Any hospital admission beyond 15 and within 28 days of treatment conclusion | difference in proportion, 95% confidence interval | 0.0 (-6.8, 6.7) |  |
| Metformin | Placebo | Bramante, USA, 2023 | RP | 564 | 562 | 1 | metformin dose was titrated over 6 days: 500 mg on day 1, 500 mg twice daily on days 2–5, then 500 mg in the morning and 1000 mg in the evening up to day 14. | Placebo | Proportion of patients hospitalized for COVID-19-related reasons by day 14 | Hospitalization rate (%), OR 95% CI | 42.3%; 0.58 [0.35–0.94] |  |
| Metformin | Placebo | Reis, Brazil, 2022 (retracted) | RP | 215 | 203 | 1 | Metformin (750 mg twice daily for 10 days) | Placebo tablets: twice diaily for 10 days | Proportion of patients hospitalized for COVID-19 at 28 days | Relative risk (RR); Bayesian Credible Interval | 1.14 (0.73 to 1.81) |  |
| Metformin; Ivermectin; Fluvoxamine | Placebo + Control | Bramante 2022, United States | RP | Metformin = 663 , Ivermectin = 410 , Fluvoxamine = 334 | Metformin control= 660, Ivermectin control= 398, Fluvoxamine control = 327 | 0 | immediate-release metformin administered with an increase in dose over 6 days to 1500 mg per day for 14 days, ivermectin at a dose of 390 to 470 μg per kilogram per day for 3 days, and fluvoxamine at a dose of 50 mg twice daily for 14 days. | The metformin control group included the patients who had received placebo, fluvoxamine alone, or ivermectin alone. Patients in the ivermectin control group were randomly assigned to receive either placebo or metformin alone. The fluvoxamine and fluvoxamine control groups were constructed similarly. The control groups that were used in the comparisons with the active-drug groups included only concurrently randomly assigned patient | Hospitalization; Metformin | Adjusted Odds Ratio (95% CI) | 0.47 (0.20-1.11) | Modified ITT population. Adjusted odds ratios and 95% confidence intervals are based on a logistic-regression model that was adjusted for baseline vaccination status and the receipt of other medications during the trial; multiple imputation was used with chained equations and predictive mean matching. |
| Metformin; Ivermectin; Fluvoxamine | Placebo + Control | Bramante 2022, United States | RP | Metformin = 663 , Ivermectin = 410 , Fluvoxamine = 335 | Metformin control= 660, Ivermectin control= 398, Fluvoxamine control = 328 | 0 | immediate-release metformin administered with an increase in dose over 6 days to 1500 mg per day for 14 days, ivermectin at a dose of 390 to 470 μg per kilogram per day for 3 days, and fluvoxamine at a dose of 50 mg twice daily for 14 days. | The metformin control group included the patients who had received placebo, fluvoxamine alone, or ivermectin alone. Patients in the ivermectin control group were randomly assigned to receive either placebo or metformin alone. The fluvoxamine and fluvoxamine control groups were constructed similarly. The control groups that were used in the comparisons with the active-drug groups included only concurrently randomly assigned patient | Hospitalization; Ivermectin | Adjusted Odds Ratio (95% CI) | 0.73 (0.19-2.77) | Modified ITT population. Adjusted odds ratios and 95% confidence intervals are based on a logistic-regression model that was adjusted for baseline vaccination status and the receipt of other medications during the trial; multiple imputation was used with chained equations and predictive mean matching. |
| Metformin; Ivermectin; Fluvoxamine | Placebo + Control | Bramante 2022, United States | RP | Metformin = 663 , Ivermectin = 410 , Fluvoxamine = 336 | Metformin control= 660, Ivermectin control= 398, Fluvoxamine control = 329 | 0 | immediate-release metformin administered with an increase in dose over 6 days to 1500 mg per day for 14 days, ivermectin at a dose of 390 to 470 μg per kilogram per day for 3 days, and fluvoxamine at a dose of 50 mg twice daily for 14 days. | The metformin control group included the patients who had received placebo, fluvoxamine alone, or ivermectin alone. Patients in the ivermectin control group were randomly assigned to receive either placebo or metformin alone. The fluvoxamine and fluvoxamine control groups were constructed similarly. The control groups that were used in the comparisons with the active-drug groups included only concurrently randomly assigned patient | Hospitalization; Fluvoxamine | Adjusted Odds Ratio (95% CI) | 1.11 (0.33-3.76) | Modified ITT population. Adjusted odds ratios and 95% confidence intervals are based on a logistic-regression model that was adjusted for baseline vaccination status and the receipt of other medications during the trial; multiple imputation was used with chained equations and predictive mean matching. |
| methylprednisolone | Standard/Usual Care | Issak, 2023, Egypt | RP | 377 | 375 | 0 | methylprednisolone 30 mg daily for 1 week in moderate cases, then reduced gradually over 2 weeks, mild cases were started at 15mg | standard COVID-19 protocol | Need for hospitalization @ day 10 | OR 95% CI, Tx vs comparator, N vs N | 24 (6.37%) vs 51 (13.6%) p = 0.001  0.43 (0.26 to 0.72) |  |
| Niclosamide + Bromhexine | Standard/Usual Care | Wannigama 2024, Thailand | RP | 172 | 336 | 0 | Participants received a 1000 mg of niclosamide tablet two times daily for a total of 14 days, in combination with one 8 mg tablet of bromhexine twice daily for a total of 10 days | Standard care (based on COVID-19 treatment guidelines at the time of the trial) | Hospitalized, requiring non-invasive mechanical ventilation or high flow oxyegenation, low flow supplemental oxygen, or not requiring supplemental oxygen, and requiring ongoing medical care at day 9 | Tx vs comparator; N (%), p-value | 0 vs 208 (61.8); p<0.0001 |  |
| Niclosamide + Bromhexine | Standard/Usual Care | Wannigama 2024, Thailand | RP | 172 | 336 | 0 | Participants received a 1000 mg of niclosamide tablet two times daily for a total of 14 days, in combination with one 8 mg tablet of bromhexine twice daily for a total of 10 days | Standard care (based on COVID-19 treatment guidelines at the time of the trial) | Hospitalized, requiring non-invasive mechanical ventilation or high flow oxyegenation, low flow supplemental oxygen, or not requiring supplemental oxygen, and requiring ongoing medical care at day 14 | Tx vs comparator; N (%), p-value | 0 vs 44 (40.3), p<0.0001 |  |
| Niclosamide + Bromhexine | Standard/Usual Care | Wannigama 2024, Thailand | RP | 172 | 336 | 0 | Participants received a 1000 mg of niclosamide tablet two times daily for a total of 14 days, in combination with one 8 mg tablet of bromhexine twice daily for a total of 10 days | Standard care (based on COVID-19 treatment guidelines at the time of the trial) | Hospitalized, requiring non-invasive mechanical ventilation or high flow oxyegenation, low flow supplemental oxygen, or not requiring supplemental oxygen, and requiring ongoing medical care at day 28 | Tx vs comparator; N (%), p-value | 0 vs 37 (66.1), p<0.0001 |  |
| Nitric Oxide-Generating Lozenges | Placebo | Bryan 2023, USA | RP | 261 | 263 | 1, African American and Hispanic patients only; aged 50-85 | Supply of study drug for 30 days (60 lozenges) and were instructed to take one Nitric Oxide-Generating lozenge twice daily approximately 12 hours apart. If a dose was missed, the next dose was to be taken at the next regular scheduled interval. Patients were instructed not to take more than 2 lozenges per day. | placebo | composite primary endpoint of hospitalization, intubation, ICU admission, dialysis and death in patients with a diagnosis of COVID-19 | Tx vs comparator; N V N, p-value | 3 (1.1%) vs 3 (1.1%), p-value = 1.00 | Only composite end point info available. non-ITT population |
| Oral N-acetylcysteine + Standard/Usual Care; Bromhexine tablets + Standard/Usual Care | Standard/Usual Care | Eslami-Ghayour, 2023, Iran | RP | Group A: 75 Group B: 75 | 75 | 0 | Group A: Oral N-acetylcysteine, 600 mg twice a day for five days + usual treatment  Group B: Bromhexine tablets, 8 mg three times a day for five days + usual treatment | Group C: Only usual treatment (Naproxen, 250 mg twice daily for five days, famotidine, 20 mg once daily for 10 days, vitamin D 50,000 per week for four weeks, vitamin C, 1000 mg daily) | Mean hospitalization time (days) | Group A vs. B vs. C:  One-Way ANOVA, p-value (95% CI) | 6.7 (± 3.3) vs. 5.3 (± 0.47) vs. 10.47 (± 2.29)  p= <0.001 | Note: ITT analysis was not explicitly mentioned in this trial. |
| Oral N-acetylcysteine + Standard/Usual Care; Bromhexine tablets + Standard/Usual Care | Standard/Usual Care | Eslami-Ghayour, 2023, Iran | RP | Group A: 75 Group B: 75 | 75 | 0 | Group A: Oral N-acetylcysteine, 600 mg twice a day for five days + usual treatment  Group B: Bromhexine tablets, 8 mg three times a day for five days + usual treatment | Group C: Only usual treatment (Naproxen, 250 mg twice daily for five days, famotidine, 20 mg once daily for 10 days, vitamin D 50,000 per week for four weeks, vitamin C, 1000 mg daily) | Number of patients hospitalized per group | Group A vs. B vs. C: p-value (95% CI) | 11 (14.66%) vs. 6 (8%) vs. 21 (28%)  p-value = 0.003 | Note: ITT analysis was not explicitly mentioned in this trial. |
| Prednisolone | Placebo | Amra, Iran, 2023 | RP | 37 | 38 | 0 | Prednisolone 50 mg/day for 5 days vs. Placebo | Placebo | Hospitalization rate at days 3, 7, 14, 21, 28 | Tx vs Comparator; Rate of hospitalization (%), p-value | 10.8% vs 7.9%, p=0.6 |  |
| Rivaroxaban | Placebo | Ananworanich, 2022, USA | RP | 192 | 199 | 0 | one 10 mg Rivaroxaban tablet, daily, for 21 days | one 10 mg multivitamin placebo tablet, daily, for 21 days | Proportion with hospitalization | Risk difference: [95% CI]  p-value | 0.41 [0.08-2.07] p=0.27 | Modified ITT population |
| Rivaroxaban | Placebo | Piazza, 2023, USA | RP | 641 | 643 | 1 | 10 mg of oral rivaroxaban daily for 35 days | 10 mg of placebo daily for 35 days | All cause hospitalization | N (%) vs. N(%)  HR/RR (95% CI) | 22 (3.3%) vs. 17 (2.6%)  1.24 (0.66-2.36) |  |
| Rivaroxaban | Placebo | Piazza, 2023, USA | RP | 641 | 643 | 1 | 10 mg of oral rivaroxaban daily for 35 days | 10 mg of placebo daily for 35 days | All cause hospitalization or all cause mortality at day 35 | N (%) vs. N(%)  HR/RR (95% CI) | 5 (0.8%) vs. 3 (0.5%)  1.67 (0.4-6.96) |  |
| rivaroxaban | Standard/Usual Care | Avezum, 2023, Brazil | RP | 327 | 330 | 0 | receive open-label rivaroxaban 10 mg OD for 14 days | routine care | hospitalisation followed the local practice and clinical judgement at each participating site | RR 95% CI | 1.13 (0.72–1.78) p = 0.610 |  |
| rivaroxaban | Standard/Usual Care | Avezum, 2023, Brazil | RP | 327 | 330 | 0 | receive open-label rivaroxaban 10 mg OD for 14 days | routine care | composite of venous thromboembolic events (VTE), need of mechanical ventilation, MACE (defined as acute myocardial infarction, stroke, or acute limb ischemia), death not attributed to major injury within 30 days from randomisation, and COVID-19 hospitalization | RR 95% CI | 0.98 (0.64-1.51) p = 1.00 |  |
| Rivaroxaban | Placebo | Ananworanich 2022, USA | RP | 222 | 222 | 0 | Rivaroxaban (one 10-mg tablet) orally daily for 21 consecutive days. | Placebo equivalent (multivitamin, 1 tablet) orally daily for 21 consecutive days | Proportion with hospitalization | Risk Difference  [95% CI] | .43  [.11 to 1.65]  P = .21 |  |
| SNG001 | Placebo | Jagannathan, 2023, US | RP | 110 | 110 | 0 | SNG001, 12 MIU/mL, single nebulized dose, once a day, for 14 days | Placebo solution, single nebulized dose, once day, for 14 days | Hospitalizations due to COVID-19 | Tx vs comparator; N (%)  p-value | 1(1%) vs 7 (6%)  p-value=0.07 |  |
| Sulodexide | Placebo | Gonzalez-Ochoa 2021, Mexico | RP | 124 | 119 | 0 | Sulodexide (oral 1,000 LRU/d) for 21 days | Placebo for 21 days | Need for hospital care | p-value, RR (95% CI) | 0.60 (0.37–0.96), p = 0.03 |  |
| Sulodexide | Placebo | Gonzalez-Ochoa 2021, Mexico | RP | 124 | 119 | 0 | Sulodexide (oral 1,000 LRU/d) for 21 days | Placebo for 21 days | Length of hospital care (days) | Tx vs comparator; mean (SD), p value, | 6.3(4.1) vs 7.8 (4.5); p = 0.21 |  |
| Tafenoquine | Placebo | Dow, 2022, USA | RP | 45 | 41 | 0 | Tafenoquine administered orally as 2 × 100 mg tablets on study days 1, 2, 3, and 10 | Matching placebo | ITT hospitalizations due to COVID-19 symptoms | Tafenoquine vs. Placebo:   Patients hospitalized:  N (%) vs. N(%)   p-value | 1 (2.2%) vs. 2 (4.9%)  p=0.60 |  |
| Upamostat | Placebo | Plasse, USA, 2023 | RP | Upamostat 200 mg: 20; Upamostat 400 mg: 21 | 20 | 1 | Upamostat 200 mg daily for 14 days  Upamostat 400 mg daily for 14 days | Placebo | Hospitalization due to worsening COVID-19 | Tx vs comparator; Proportion hospitalized | 0% vs 15%, p = 0.032 |  |

**Table 3b:** Effect of antivirals on any hospital admission and/or duration

| **Treatment Name** | **Comparator category** | **Study, country** | **Tx classification** | **Treatment (N)** | **Comparator (N)** | **Unique population** | **Tx dosage and duration** | **Type of comparator** | **Outcome Definition** | **Effect measure** | **Effect estimate** | **Note** |
| --- | --- | --- | --- | --- | --- | --- | --- | --- | --- | --- | --- | --- |
| Arbidol + hydroxychloroquine | Hydroxychloroquine + Standard/Usual Care | Ghaderkhani, 2022, Iran | AV | 28 | 25 | 0 | The case group received hydroxychloroquine (HCQ) at a dose of 200 mg every 12 hours and Umifenovir at a dose of 200 mg every 8 hours for 10 days. | The control group received the national treatment protocol proposed for COVID-19 (28), including HCQ every 12 hours for 10 days and acetaminophen and diphenhydramine oral syrup if needed | Need for hospitalization (yes) | p-value | 0.981 |  |
| Bemnifosbuvir | Placebo | Horga 2023, Multiple | AV | 137 | 70 | 0 | Oral bemnifosbuvir 550 mg (two 275 mg tablets a day), for 5 days | Placebo treatment, 275 mg, two times a day, for 5 days | Proportion of patients with ≥ 1 COVID-19 related medically attended visit throughout the study | Median (97.5% CI from Kaplan-Meier curves), n | 14 (4.05-16.38) vs. 10 (4.20-24.37) | Note: Variables unclear (unsure if this includes hospitalizations) |
| Bemnifosbuvir | Placebo | Horga 2023, Multiple | AV | 137 | 70 | 0 | Oral bemnifosbuvir 550 mg (two 275 mg tablets a day), for 5 days | Placebo treatment, 275 mg, two times a day, for 5 days | Proportion of patients requiring hospitalization for COVID-19 (ad hoc) – Patients aged ≤ 40 years | Median (97.5% CI from Kaplan-Meier curves), n | 2 (0-8.65) vs. 1 (0-11.95) |  |
| Bemnifosbuvir | Placebo | Horga 2023, Multiple | AV | 137 | 70 | 0 | Oral bemnifosbuvir 550 mg (two 275 mg tablets a day), for 5 days | Placebo treatment, 275 mg, two times a day, for 5 days | Proportion of patients requiring hospitalization for COVID-19 (ad hoc) – Patients aged > 40 years | Median (97.5% CI from Kaplan-Meier curves), n | 2 (0-7.81) vs. 6 (1.15-29.62) |  |
| Bemnifosbuvir | Placebo | Horga 2023, Multiple | AV | 137 | 70 | 0 | Oral bemnifosbuvir 550 mg (two 275 mg tablets a day), for 5 days | Placebo treatment, 275 mg, two times a day, for 5 days | Proportion of patients requiring hospitalization for COVID-19 | Median (97.5% CI from Kaplan-Meier curves), n | 4 (0.00-6.51) vs. 7 (1.25-18.75) |  |
| Favipiravir | Placebo | Vaezi, Iran, 2023 | AV | 38 | 39 | 1 | Favipiravir 1600 mg daily for five days | Placebo | Change in viral load (SARS-CoV-2 RT-PCR) | Tx vs comparator; Proportion of patients hospitalized; validated by chi-square tests | 4 (10.5%) vs 2 (5.1%), p-value = 0.3 |  |
| Favipiravir | Placebo | Holubar 2022, US | AV | 59 | 57 | 0 | favipiravir 1800 mg twice daily (BID) on day 1, then 800 mg BID on days 2–10. | Placebo | Hospitalizations by day 28 | Tx vs comparator, %, p value | 5 vs 0; p=0.06 | Among the ITT population |
| Favipiravir | Placebo | McMahon, Australia 2022 | AV | 99 | 100 | 1 | 1800 mg Favipiravir on day 1, then 800 mg twice daily for 14 days | Matching placebo | Progression to hospitalization | Tx vs comparator; Proportion of patients hospitalized | 14 (14.14%) vs 9 (9.0%); p = 0.38 |  |
| Favipiravir + Standard/Usual Care | Standard/Usual Care | Tehrani, Iran, 2022 | AV | 38 | 40 | 0 | in addition to supportive therapy, received oral favipiravir (Cytovex, made by Abidi company in Iran) at a dose of 1600 mg every 12 h for the first day and 600 mg every 12 h for the next 4 d. | Patients in the control group received supportive therapy. According to the national guideline for COVID-19 management at the time of the study, outpatients with moderate severity received the following oral drugs: acetaminophen (500 mg every 6 h, until fever stops), diphenhydramine (10-15 ml every 6-8 h, in cases of coughing), vitamin D3  (50000 IU, weekly, if there was no history of vitamin D consumption), zinc and selenium supplements (daily)[13]. | Hospitalization rate during the 7d follow-up period | Tx vs comparator; %, p-value | 26.3% vs. 40.0%; p=0.200) |  |
| IBIO123 | Placebo | Maranda, 2023, South Africa | AV | Phase I FAS:  83   Phase II Modified FAS:  88 | Phase I FAS Placebo:  28  Phase II Modified FAS Placebo:  30 | 0 | A single dose of 0·1 mL of either IBIO123 (1 mg) was administered to participants in cohort 1, 0·5 mL (5 mg) in cohort 2, and 1·0 mL (10 mg) in cohort 3. After completion of phase 1, the data monitoring committee approved the dose of 10 mg IBIO123 and the corresponding placebo volume for phase 2 | A single dose of 0·1 mL (1 mg) of placebo was administered to participants in cohort 1, 0·5 mL (5 mg) in cohort 2, and 1·0 mL (10 mg) in cohort 3. After completion of phase 1, the data monitoring committee approved the dose of 10 mg corresponding placebo volume for phase 2 | Hospitalizations | N/A | One serious adverse event was noted in the placebo group (bacterial pneumonia). The participant with this serious adverse event was hospitalised and died before the end of the study. | Not quantitatively measured, mentioned in the Results section |
| Leriterelvir | Placebo | Zhan, 2024, China | AV | 680 | 679 | 0 | Leritrelvir, 400 mg, three times a day orally for 5 days | Placebo, three times a day orally for 5 days | The disease progresses to hospitalization or death by day 29 | Tx vs comparator; N (%) | 0 vs 0 |  |
| Leriterelvir | Placebo | Zhan, 2024, China | AV | 680 | 679 | 0 | Leritrelvir, 400 mg, three times a day orally for 5 days | Placebo, three times a day orally for 5 days | Hospitalization for all-cause death from progression of COVID-19 related illness by day 29 | Tx vs comparator; N (%) | 0 vs 0 |  |
| Lopinavir/ritonavir | Placebo | Kaizer, USA, 2023 | AV | 220 | 226 | 0 | Twice-daily oral LPV/r (400 mg/100 mg) for 14 days | matching placebo | Hospitalization by day 28 | N (%) | 7 (3.2%) vs 6 (2.7%) |  |
| Molnupiravir | Placebo | Chawla, USA, 2023 | AV | 716 | 717 | 0 | MOV 800mg Q12H twice daily (every 12 hours) for 5 days | Placebo | All-cause hospitalization or death through day 29 | Tx vs comparator; Event rate (%), % reduction | 6.8 vs 9.7; 30.4% reduction |  |
| Molnupiravir | Placebo | JaykBernal 2022, Multiple countries (USA, Ukraine, Taiwan, UK, Spain, South Africa, Russian Federation, Philippines, Mexico, Japan, Italy, Guatemala, Germany, France, Egypt, Colombia, Chilie, Canada, Brazil, Argentina) | AV | 709 | 699 | 0 | 800 mg of molnupiravir twice daily for 5 days | placebo twice daily for 5 days | only Covid-19–related hospitalizations or deaths. hospitalization for any cause (defined as ≥24 hours of acute care in a hospital or any similar facility) | % difference, 95% CI | -2.8%; −5.7 to 0.0 |  |
| Molnupiravir + Standard/Usual Care | Standard/Usual Care | Butler, UK, 2023 | AV | 12,774 | 12,934 | 1 | 800 mg molnupiravir orally twice daily for 5 days. | Managing symptoms with antipyretics; However, patients at very high risk (ie, those with impaired immune systems or who are extremely clinically vulnerable— roughly 1·8 million people in the UK) are eligible to receive monoclonal antibodies (sotrovimab), intravenous antivirals (remdesivir), and oral antivirals (molnupiravir or nirmatrelvir–ritonavir) from specialist regional COVID-19 clinics (Ages 18-69 & 70-74; Tx n = 1,116 [9%]; Control: 1,111 [9%]) | Hospitalization or death within 28 days | Adjusted Odds Ratio (OR) | 1.06 [ 0.81–1.41]; p= 0.33 |  |
| Molnupiravir + Standard/Usual Care | Placebo + Standard/Usual Care | Khoo, UK 2023 | AV | 90 | 90 | 1 | molnupiravir (orally; 800 mg twice daily for 5 days) plus SoC | Placebo + SoC | Hospitalization rates at days 15 | Tx vs comparator; Proportion % | 0 vs 3 |  |
| Molnupiravir + Standard/Usual Care | Placebo + Standard/Usual Care | Khoo, UK 2024 | AV | 90 | 90 | 1 | molnupiravir (orally; 800 mg twice daily for 5 days) plus SoC | Placebo + SoC | Hospitalization rates at days 30 | Tx vs comparator; Proportion % | 4 vs 0 |  |
| Molnupiravir; Ritonavir-boosted nirmatrelvir | No study drug | Schilling, 2023, Multiple | AV | Group A: 58  Group B: 65 | 84 | 1 | Oral molnupiravir 800 mg was given twice a day for 5 days; oral nirmatrelvir 300 mg with oral ritonavir 100 mg was given twice a day for 5 days. | No study drug | Any severe disease or hospitalizations | N/A | No patients developed sever disease or were hospitalized. | Unique patient population: Previously healthy adults aged 18-50 years |
| SNG001 | Placebo | Francis, 2023, Britain | AV | 56 | 58 | 0 | Inhale SNG001, once daily via nebulizer, for 14 days | Placebo, once only via nebulizer, for 14 days | Hospitalizations | N vs N, Tx vs comparator | 0 vs 2 | Only 2 ppts from the placebo group were hospitalized. Endpoint was not analysed |
| Sofosbuvir + Daclatasvir + Hydroxychloroquine | Hydroxychloroquine + Standard/Usual Care | Roozbeh 2021, Iran | AV | 27 | 28 | 0 | Single daily oral tablet containing 400 mg sofosbuvir and 60 mg daclatasvir with hydroxychloroquine (200 mg twice daily) for 7 days, or until death/hospitalization | **Hydrosxychloroquine and Standard care** Standard care (azithromycin capsules (500 mg for 6 days) with naproxen tablets (500 mg, twice daily for 7 days), as well as 40 mg pantoprazole tablets.) | Hospital admission | Tx vs comparator; p value, n (%) | 1 (4) vs 4 (14), p = 0.352 |  |
| Sofosbuvir + Standard/Usual Care | Placebo + Standard/Usual Care | Shoushtari, 2024, Iran | AV | 180 | 180 | 0 | Under usual treatment + sofosbuvir 400 mg, one pill daily for 7 days | Standard of care (under usual treatment + placebo one tablet daily for 7 days) | Mean days of hospitalization | Tx vs comparator; N (SD), p-value | 3 (0.8) vs 4 (1.1), p=0.15 | Note: ITT analysis was not explicitly mentioned in this trial. |
| Sofosbuvir + Standard/Usual Care | Placebo + Standard/Usual Care | Shoushtari, 2024, Iran | AV | 180 | 180 | 0 | Under usual treatment + sofosbuvir 400 mg, one pill daily for 7 days | Standard of care (under usual treatment + placebo one tablet daily for 7 days) | Frequency of the need for hospitalization | Tx vs comparator; N (%), p-value | 4 (2.2) vs 7 (3.8) | Note: ITT analysis was not explicitly mentioned in this trial. |

**Table 3c:** Effect of both antivirals and repurposed treatments on any hospital admission and/or duration

| **Treatment Name** | **Comparator category** | **Study, country** | **Tx classification** | **Treatment (N)** | **Comparator (N)** | **Unique population** | **Tx dosage and duration** | **Type of comparator** | **Outcome Definition** | **Effect measure** | **Effect estimate** | **Note** |
| --- | --- | --- | --- | --- | --- | --- | --- | --- | --- | --- | --- | --- |
| Artesunate-Amodiaquine + Standard/Usual Care; Pyronaridine-artesunate + Standard/Usual Care; Favipiravir plus Nitazoxanide + Standard/Usual Care; or Sofosbuvir-Daclatasvir + Standard/Usual Care | Standard/Usual Care | Chandiwana 2022, Johannesburg, South Africa | Both | ASAQ (n = 39), PA (n = 36), FPV + NTZ (n = 37), SOF-DCV (n = 35) | SOC (n = 39) | 0 | Paracetamol 1000 mg 6-hourly as needed; ASAQ 200/540 mg once daily for 3 days; PA once daily for 3 days dosed by body weight, 540/180 mg for 45 to <65 kg, and 720/240 for ≥65 kg; FPV, 1600 mg 12-hourly for 1 day, then 600 mg 12-hourly for 6 days; NTZ 1000 mg 12-hourly for 7 days with food; SOF-DCV (Mylan Laboratories, India), 400/60 mg once daily for 7 days |  | hospitalisation incidence to day 28 | ASAQ vs PA vs FPV+NTZ vs SOF-DCV; N% | 0 vs 1 vs 0 vs 1 | Modified ITT population. |
| Hydroxychloroquine; Lopinavir-ritonavir | Placebo | Reis 2021, Brazil | Both | Hydroxychloroquine = 214, Lopinavir-Ritonavir = 244 | Control = 227 | 0 | Hydroxychloroquine group received a loading dose of 800 mg at the time of randomization and then 400 mg in daily doses at 8:00 AM for 9 days. Lopinavir-ritonavir group received a loading dose of 800 mg of Lopinavir and 200 mg of ritonavir at the first 2 intakes, followed by 400 mg of lopinavir and 100 mg of ritonavir every 12 hours for the next 9 days. | Placebo group received corresponding tablets of inert material (talc) | COVID-associated hospitalization and death, Hydroxychloroquine | HR 95% CI | 0.76 (0.30-1.88) |  |
| Hydroxychloroquine; Lopinavir-ritonavir | Placebo | Reis 2021, Brazil | Both | Hydroxychloroquine = 214, Lopinavir-Ritonavir = 245 | Control = 227 | 0 | Hydroxychloroquine group received a loading dose of 800 mg at the time of randomization and then 400 mg in daily doses at 8:00 AM for 9 days. Lopinavir-ritonavir group received a loading dose of 800 mg of Lopinavir and 200 mg of ritonavir at the first 2 intakes, followed by 400 mg of lopinavir and 100 mg of ritonavir every 12 hours for the next 9 days. | Placebo group received corresponding tablets of inert material (talc) | COVID-associated hospitalization and death, Liponavir-ritonavir | HR 95% CI | 1.16 (0.53-2.56), |  |
| Hydroxychloroquine; Lopinavir-ritonavir | Placebo | Reis 2021, Brazil | Both | Hydroxychloroquine = 214, Lopinavir-Ritonavir = 244 | Control = 227 | 0 | Hydroxychloroquine group received a loading dose of 800 mg at the time of randomization and then 400 mg in daily doses at 8:00 AM for 9 days. lopinavir-ritonavir group received a loading dose of 800 mg of Lopinavir and 200 mg of ritonavir at the first 2 intakes, followed by 400 mg of lopinavir and 100 mg of ritonavir every 12 hours for the next 9 days. | Placebo group received corresponding tablets of inert material (talc) | Hospital admissions for any cause, Hydroxychloroquine | HR 95% CI | 0.96 (0.42-2.17) |  |
| Hydroxychloroquine; Lopinavir-ritonavir | Placebo | Reis 2021, Brazil | Both | Hydroxychloroquine = 214, Lopinavir-Ritonavir = 245 | Control = 227 | 0 | Hydroxychloroquine group received a loading dose of 800 mg at the time of randomization and then 400 mg in daily doses at 8:00 AM for 9 days. lopinavir-ritonavir group received a loading dose of 800 mg of Lopinavir and 200 mg of ritonavir at the first 2 intakes, followed by 400 mg of lopinavir and 100 mg of ritonavir every 12 hours for the next 9 days. | Placebo group received corresponding tablets of inert material (talc) | Hospital admissions for any cause; Liponavir-ritonavir | HR 95% CI | 1.22 (0.58-2.57) |  |
| Nitazoxanide | Placebo | Rocco, 2021 | Both | 194 | 198 | 0 | Nitazoxanide 500mg three times daily for 5 days | 1:1 placebo matching | incidence of hospital admission after completion of therapy | hospital admission incidence post-therapy | 0 |  |

**Table 4a:** Effect of repurposed treatments on ICU admission and/or duration

| **Treatment Name** | **Comparator category** | **Study, country** | **Tx classification** | **Treatment (N)** | **Comparator (N)** | **Unique population** | **Tx dosage and duration** | **Type of comparator** | **Outcome Definition** | **Effect measure** | **Effect estimate** | **Notes** |
| --- | --- | --- | --- | --- | --- | --- | --- | --- | --- | --- | --- | --- |
| Azithromycin | Standard/Usual Care | Hinks 2021 | RP | 145 | 147 | 0 | 500 mg Azithromycin once daily orally for 14 days | Standard care | the proportion of participants with hospital admission with respiratory failure  requiring non-invasive mechanical ventilation or  invasive mechanical ventilation | Tx vs comparator; p value, N (%) | 2/145 (1%) vs 2/147 (1%); p=1·00, | the values on the table for mechanical ventialtion specifies Level 2 and 3 ventilation |
| Azithromycin + Standard/Usual Care | Standard/Usual Care | Butler 2021, UK | RP | 500 | 629 | 1 | Oral Azithromycin 500 mg once daily for three days plus usual care | Usual care | Intensive care unit  admission | RR 95% Bayseian credible interval | 0·76 (0·18 to 3·15) | n value used for Azithromycin in primary analysis population and n value used for Usual care in concurrent randomisation analysis population. Unadjusted relative risk  due to low event rate. |
| Azithromycin + Standard/Usual Care | Standard/Usual Care | Butler 2021, UK | RP | 500 | 629 | 1 | Oral Azithromycin 500 mg once daily for three days plus usual care | Usual care | Oxygen administration | RR 95% Bayseian credible interval | 0·84 (0·38 to 1·85) | n value used for Azithromycin in primary analysis population and n value used for Usual care in concurrent randomisation analysis population. Unadjusted relative risk due to low event rate. |
| Azithromycin + Standard/Usual Care | Standard/Usual Care | Butler 2021, UK | RP | 500 | 629 | 1 | Oral Azithromycin 500 mg once daily for three days plus usual care | Usual care | Mechanical ventilation | RR 95% Bayseian credible interval | 0·50 (0·10 to 2·59) | n value used for Azithromycin in primary analysis population and n value used for Usual care in concurrent randomisation analysis population. Unadjusted relative risk due to low event rate. |
| Colchicine | Placebo | Tardif 2021, Brazil, Canada, Greece, South Africa, Spain, and the USA | RP | 2235 | 2253 | 0 | 0·5 mg colchicine orally administered twice per day for the first 3 days and then  once per day for 27 days thereafter | Matching placebo | Mechanical ventilation | Odds ratio  (95% CI) | 0·53 (0·25–1·09) |  |
| Doxycycline + Standard/Usual Care | Standard/Usual Care | Butler 2021, UK | RP | 780 | 644 | 0 | usual care plus oral doxycycline (200 mg on day 1, followed by 100 mg once daily for the following 6 days) | usual care only: largely supportive. | Oxygen administration | RR (95% CI) | 0·98 (0·55 to 1·76) | n value from concurrent randomisation analysis population. Unadjusted relative risk due to low event rate |
| Doxycycline + Standard/Usual Care | Standard/Usual Care | Butler 2021, UK | RP | 780 | 644 | 0 | usual care plus oral doxycycline (200 mg on day 1, followed by 100 mg once daily for the following 6 days) | usual care only: largely supportive. | Mechanical ventilation | RR (95% CI) | 0·49 (0·12 to 2·05) | n value from concurrent randomisation analysis population. Unadjusted relative risk due to low event rate |
| Doxycycline + Standard/Usual Care | Standard/Usual Care | Butler 2021, UK | RP | 780 | 644 | 0 | usual care plus oral doxycycline (200 mg on day 1, followed by 100 mg once daily for the following 6 days) | usual care only: largely supportive. | Intensive care unit admission | RR (95% CI) | 6/620 (1·0%) | n value from concurrent randomisation analysis population. Unadjusted relative risk due to low event rate |
| Fluvoxamine | Placebo | Lenze 2020, St. Louis metropolitan area (Missouri and Illinois) | RP | Fluvoxamine (n = 80) | Placebo (n = 72) | 0 | 100 mg of fluvoxamine for 15 days | 100 mg of placebo for 15 days | Oxygen saturation <92% plus supplemental oxygen needed and hospitalization related to dyspnea or hypoxia plus ventilator support needed for ≥3 days | Absolute difference, 95 CI, p-value and Tx vs comparator; N (%) | −1.4 (−8.4 to 4.4) P value = 0.36;  0 vs 1 (1.4) |  |
| Fluvoxamine | Standard/Usual Care | Wannigama 2024, Thailand | RP | 162 | 336 | 0 | The fluvoxamine-only arm received fluvoxamine (immediate release) 50 mg orally, one tablet in the morning and one tablet at bedtime for the first two days, then escalated to one 50 mg one tablet in the morning and two tablets before bedtime for days 3 through 12, ending with one 50 mg one tablet in the morning and one tablet at bedtime for days 13 and 14 | Standard care (based on COVID-19 treatment guidelines at the time of the trial) | Hospitalized, requiring invasive mechanical ventilation or extr5acorporeal membrane oxyegenation at day 9 | Tx vs comparator; N (%) | 0 vs 19 (5.7); p<0.0001 |  |
| Fluvoxamine | Standard/Usual Care | Wannigama 2024, Thailand | RP | 162 | 336 | 0 | The fluvoxamine-only arm received fluvoxamine (immediate release) 50 mg orally, one tablet in the morning and one tablet at bedtime for the first two days, then escalated to one 50 mg one tablet in the morning and two tablets before bedtime for days 3 through 12, ending with one 50 mg one tablet in the morning and one tablet at bedtime for days 13 and 15 | Standard care (based on COVID-19 treatment guidelines at the time of the trial) | Hospitalized, requiring invasive mechanical ventilation or extr5acorporeal membrane oxyegenation at day 14 | Tx vs comparator; N (%) | 0 vs 8 (7.3), p<0.0001 |  |
| Fluvoxamine | Standard/Usual Care | Wannigama 2024, Thailand | RP | 162 | 336 | 0 | The fluvoxamine-only arm received fluvoxamine (immediate release) 50 mg orally, one tablet in the morning and one tablet at bedtime for the first two days, then escalated to one 50 mg one tablet in the morning and two tablets before bedtime for days 3 through 12, ending with one 50 mg one tablet in the morning and one tablet at bedtime for days 13 and 16 | Standard care (based on COVID-19 treatment guidelines at the time of the trial) | Hospitalized, requiring invasive mechanical ventilation or extr5acorporeal membrane oxyegenation at day 28 | Tx vs comparator; N (%) | 0 vs 5 (8.9), p<0.0001 |  |
| Fluvoxamine | Placebo | Reis 2022, Brazil | RP | 741 | 756 | 0 | fluvoxamine (Luvox, Abbott) at a dose of 100 mg twice a day for 10 days starting directly after randomisation (day 1). | Corresponding placebo starting directly after randomisation (day 1). | Mechanical ventilation | Median (IQR) | 0·77 (0·45–1·30) |  |
| Fluvoxamine + Bromhexine | Standard/Usual Care | Wannigama 2024, Thailand | RP | 178 | 336 | 0 | The fluvoxamine-only arm received fluvoxamine (immediate release) 50 mg orally, one tablet in the morning and one tablet at bedtime for the first two days, then escalated to one 50 mg one tablet in the morning and two tablets before bedtime for days 3 through 12, ending with one 50 mg one tablet in the morning and one tablet at bedtime for days 13 and 14. Plus one 8 mg bromhexine tablet twice daily for 10 days | Standard care (based on COVID-19 treatment guidelines at the time of the trial) | Hospitalized, requiring invasive mechanical ventilation or extr5acorporeal membrane oxyegenation at day 9 | Tx vs comparator; N (%), p-value | 0 vs 19 (5.7); p<0.0001 |  |
| Fluvoxamine + Bromhexine | Standard/Usual Care | Wannigama 2024, Thailand | RP | 178 | 336 | 0 | The fluvoxamine-only arm received fluvoxamine (immediate release) 50 mg orally, one tablet in the morning and one tablet at bedtime for the first two days, then escalated to one 50 mg one tablet in the morning and two tablets before bedtime for days 3 through 12, ending with one 50 mg one tablet in the morning and one tablet at bedtime for days 13 and 14. Plus one 8 mg bromhexine tablet twice daily for 10 days | Standard care (based on COVID-19 treatment guidelines at the time of the trial) | Hospitalized, requiring invasive mechanical ventilation or extr5acorporeal membrane oxyegenation at day 14 | Tx vs comparator; N (%), p-value | 0 vs 8 (7.3), p<0.0001 |  |
| Fluvoxamine + Bromhexine | Standard/Usual Care | Wannigama 2024, Thailand | RP | 178 | 336 | 0 | The fluvoxamine-only arm received fluvoxamine (immediate release) 50 mg orally, one tablet in the morning and one tablet at bedtime for the first two days, then escalated to one 50 mg one tablet in the morning and two tablets before bedtime for days 3 through 12, ending with one 50 mg one tablet in the morning and one tablet at bedtime for days 13 and 14. Plus one 8 mg bromhexine tablet twice daily for 10 days | Standard care (based on COVID-19 treatment guidelines at the time of the trial) | Hospitalized, requiring invasive mechanical ventilation or extr5acorporeal membrane oxyegenation at day 28 | Tx vs comparator; N (%), p-value | 0 vs 5 (8.9), p<0.0001 |  |
| Fluvoxamine + Budesonide | Placebo | Reis, Brazil, 2023 | RP | 738 | 738 | 0 | fluvoxamine (100 mg twice daily for 10 days) plus inhaled budesonide (800 mcg twice daily for 10 days) | matching placebos | Mechnical ventilation | RR 95% CrI | 2.44 (0.18-76.66) |  |
| Fluvoxamine + Cyproheptadine | Standard/Usual Care | Wannigama 2024, Thailand | RP | 147 | 336 | 0 | The fluvoxamine-only arm received fluvoxamine (immediate release) 50 mg orally, one tablet in the morning and one tablet at bedtime for the first two days, then escalated to one 50 mg one tablet in the morning and two tablets before bedtime for days 3 through 12, ending with one 50 mg one tablet in the morning and one tablet at bedtime for days 13 and 14. Plus one 4 mg cyproheptadine tablet, three times a day for 14 days | Standard care (based on COVID-19 treatment guidelines at the time of the trial) | Hospitalized, requiring invasive mechanical ventilation or extr5acorporeal membrane oxyegenation at day 9 | Tx vs comparator; N (%), p-value | 0 vs 19 (5.7); p<0.0001 |  |
| Fluvoxamine + Cyproheptadine | Standard/Usual Care | Wannigama 2024, Thailand | RP | 147 | 336 | 0 | The fluvoxamine-only arm received fluvoxamine (immediate release) 50 mg orally, one tablet in the morning and one tablet at bedtime for the first two days, then escalated to one 50 mg one tablet in the morning and two tablets before bedtime for days 3 through 12, ending with one 50 mg one tablet in the morning and one tablet at bedtime for days 13 and 14. Plus one 4 mg cyproheptadine tablet, three times a day for 14 days | Standard care (based on COVID-19 treatment guidelines at the time of the trial) | Hospitalized, requiring invasive mechanical ventilation or extr5acorporeal membrane oxyegenation at day 14 | Tx vs comparator; N (%), p-value | 0 vs 8 (7.3), p<0.0001 |  |
| Fluvoxamine + Cyproheptadine | Standard/Usual Care | Wannigama 2024, Thailand | RP | 147 | 336 | 0 | The fluvoxamine-only arm received fluvoxamine (immediate release) 50 mg orally, one tablet in the morning and one tablet at bedtime for the first two days, then escalated to one 50 mg one tablet in the morning and two tablets before bedtime for days 3 through 12, ending with one 50 mg one tablet in the morning and one tablet at bedtime for days 13 and 14. Plus one 4 mg cyproheptadine tablet, three times a day for 14 days | Standard care (based on COVID-19 treatment guidelines at the time of the trial) | Hospitalized, requiring invasive mechanical ventilation or extr5acorporeal membrane oxyegenation at day 28 | Tx vs comparator; N (%), p-value | 0 vs 5 (8.9), p<0.0001 |  |
| Fluvoxamine + favipiravir; Fluvoxamine + favipiravir + dexamethasone | Favipiravir | Siripongboonsitti, 2023, Thailand | RP | 134 | 132 | 1 | 50 mg twice daily of fluvoxamine for 10 days and favipiravir; patients with moderate illness also received 6 mg per day of dexamethasone for 10 days | Each group received 3600 mg of FPV loading dose on day 0 and 1600 mg per day of FPV from days 1-4 in mild COVID-19 and days 1-9 in moderate COVID-19. In a patient with obesity, the FPV loading dose was 4800 mg on day 0, then 2000 mg per day of FPV on days later | Mechanical ventillation, mild illness | Tx vs comparator, N vs N, p-value | 0 vs 0, p = 1.000 |  |
| Fluvoxamine + favipiravir; Fluvoxamine + favipiravir + dexamethasone | Favipiravir | Siripongboonsitti, 2023, Thailand | RP | 134 | 132 | 1 | 50 mg twice daily of fluvoxamine for 10 days and favipiravir; patients with moderate illness also received 6 mg per day of dexamethasone for 10 days | Each group received 3600 mg of FPV loading dose on day 0 and 1600 mg per day of FPV from days 1-4 in mild COVID-19 and days 1-9 in moderate COVID-19. In a patient with obesity, the FPV loading dose was 4800 mg on day 0, then 2000 mg per day of FPV on days later | Mechanical ventillation, moderate illness | Tx vs comparator, N vs N, p-value | 0 vs 0, p = 1.000 |  |
| Fluvoxamine + favipiravir; Fluvoxamine + favipiravir + dexamethasone | Favipiravir | Siripongboonsitti, 2023, Thailand | RP | 134 | 132 | 1 | 50 mg twice daily of fluvoxamine for 10 days and favipiravir; patients with moderate illness also received 6 mg per day of dexamethasone for 10 days | Each group received 3600 mg of FPV loading dose on day 0 and 1600 mg per day of FPV from days 1-4 in mild COVID-19 and days 1-9 in moderate COVID-19. In a patient with obesity, the FPV loading dose was 4800 mg on day 0, then 2000 mg per day of FPV on days later | ICU admission, mild illness | Tx vs comparator, N vs N, p-value | 0 vs 0, p = 1.000 |  |
| Fluvoxamine + favipiravir; Fluvoxamine + favipiravir + dexamethasone | Favipiravir | Siripongboonsitti, 2023, Thailand | RP | 134 | 132 | 1 | 50 mg twice daily of fluvoxamine for 10 days and favipiravir; patients with moderate illness also received 6 mg per day of dexamethasone for 10 days | Each group received 3600 mg of FPV loading dose on day 0 and 1600 mg per day of FPV from days 1-4 in mild COVID-19 and days 1-9 in moderate COVID-19. In a patient with obesity, the FPV loading dose was 4800 mg on day 0, then 2000 mg per day of FPV on days later | ICU adimssion, moderate illness | Tx vs comparator, N vs N, p-value | 0 vs 0, p = 1.000 |  |
| Hydroxychloroquine | Standard/Usual Care | Mitja 2021, Spain | RP | 136 | 157 | 0 | HCQ - Dolquine® 800 mg on day 1, followed by 400 mg once daily for six days) | Usual care | Hospitalization requiring mechanical ventilation | Tx vs comparator; Events (%) | 0 vs 0 |  |
| Hydroxychloroquine | Placebo | Avezum, Brazil, 2022 | RP | 689 | 683 | 1 | hydroxychloroquine 400 mg twice a day in the first day, 400 mg once daily thereafter for a total of seven days | Placebo twice daily in the first day, then once daily thereafter for a total of seven days | Need for ICU admission within 30 days | Risk Ratio (RR) | 0.84 [ 0.43–1.61], p = 0.59 |  |
| Hydroxychloroquine | Placebo | Avezum, Brazil, 2022 | RP | 689 | 683 | 1 | hydroxychloroquine 400 mg twice a day in the first day, 400 mg once daily thereafter for a total of seven days | Placebo twice daily in the first day, then once daily thereafter for a total of seven days | Requirement of invasive mechnical ventilation | Risk ratio (RR) | 1.32 (0.46, 3.79) 0.6006 | Mechanical ventilation |
| Hydroxychloroquine | Placebo | Schwartz 2021 | RP | 111 | 37 | 0 | hydroxychloroquine dose was 800 mg orally in divided  doses on day 1 followed by 200 mg twice daily for 4 days | identical matching placebo (12 tablets over 5 days) | Admission to ICU within   30 d | Tx vs comparator; N% | 1 (0.9) vs 0 |  |
| Ivermectin | Standard/Usual Care | Hayward, 2024, UK | RP | 2250 | 1869 | 0 | Participants were randomised to usual care plus ivermectin tablets (target 300–400 μg/kg per dose, once daily for 3 days) or usual care plus other interventions | Usual care | ICU admission | Estimated difference median | 4.2 (0.47 to 198.5) | NOTE: Based on "Concurrently and eligible randomized population" |
| Ivermectin | Placebo | Reis 2022, Brazil | RP | 679 | 679 | 0 | Ivermectin 400 microgram/kg for 3 days, once per day | placebos that were used in the trial involved regimens of 1, 3, 10, or 14 days in duration, according to the various comparator groups in the trial at the time of randomization | Mechanical ventilation | RR, 95% CI | 0.77 (0.43 to 1.36) |  |
| Ivermectin | Placebo | Mirahmadizadeh 2022, Iran | RP | Ivermectin (S) = 130, Ivermectin (D) = 131 | Placebo = 130 | 0 | Single dose of ivermectin (3 mg tablet, cumulative dose of 12 mg, Stat. placebo tablets, at the second day) and double-dose ivermectin (3 mg tablet for 2 days, cumulative dose of 24 mg) or placebo at the day of RT-PCR-positive testing (enrolment day) and the next day. | Placebo | Need for machine ventilation | Absolute difference in proportions; 95% CI | -1.5 [-4.5, 1.5] | Ivermectin (Single Dose =S) Ivermectin (Double dose = D) |
| Ivermectin + Standard/Usual Care | Placebo + Standard/Usual Care | Vallejos 2021, Argentina | RP | 250 | 251 | 1 | Ivermectin plus standard of care (SOC) from randomization to the End Of Study (EOS) visit.  X tablets of 6 mg each at inclusion and another X tablets of 6 mg each 24 h after the first dose.  (The dose of ivermectin used was the approved dose in Argentina for the treatment of other diseases, such as parasitic diseases, and it was staggered according to weight.) | Placebo plus Standard of care (SOC)   (The SOC was in accordance with the recommendations of the Argentine Ministry of Health) | use of invasive mechanical ventilatory support (MVS) | OR 95% CI | 1.34 (0.30–6.07);  p = 0.7 |  |
| Losartan | Placebo | Puskarich, 2021 | RP | 58 | 59 | 0 | Losartan 25mg, self-administered orally twice daily for 10 days for participants with eGFR >60 mL/min/1.73 m2, and once daily for those with eGFR 30–60 mL/min/1.73 m2. | 1:1 placebo | ICU admission | difference in proportion, 95% confidence interval | 0.0 (-8.2, 8.1) |  |
| Niclosamide + Bromhexine | Standard/Usual Care | Wannigama 2024, Thailand | RP | 172 | 336 | 0 | Participants received a 1000 mg of niclosamide tablet two times daily for a total of 14 days, in combination with one 8 mg tablet of bromhexine twice daily for a total of 10 days | Standard care (based on COVID-19 treatment guidelines at the time of the trial) | Hospitalized, requiring invasive mechanical ventilation or extr5acorporeal membrane oxyegenation at day 9 | Tx vs comparator; N (%), p-value | 0 vs 19 (5.7); p<0.0001 |  |
| Niclosamide + Bromhexine | Standard/Usual Care | Wannigama 2024, Thailand | RP | 172 | 336 | 0 | Participants received a 1000 mg of niclosamide tablet two times daily for a total of 14 days, in combination with one 8 mg tablet of bromhexine twice daily for a total of 10 days | Standard care (based on COVID-19 treatment guidelines at the time of the trial) | Hospitalized, requiring invasive mechanical ventilation or extr5acorporeal membrane oxyegenation at day 14 | Tx vs comparator; N (%), p-value | 0 vs 8 (7.3), p<0.0001 |  |
| Niclosamide + Bromhexine | Standard/Usual Care | Wannigama 2024, Thailand | RP | 172 | 336 | 0 | Participants received a 1000 mg of niclosamide tablet two times daily for a total of 14 days, in combination with one 8 mg tablet of bromhexine twice daily for a total of 10 days | Standard care (based on COVID-19 treatment guidelines at the time of the trial) | Hospitalized, requiring invasive mechanical ventilation or extr5acorporeal membrane oxyegenation at day 28 | Tx vs comparator; N (%), p-value | 0 vs 5 (8.9), p<0.0001 |  |
| Oral N-acetylcysteine + Standard/Usual Care; Bromhexine tablets + Standard/Usual Care | Standard/Usual Care | Eslami-Ghayour, 2023, Iran | RP | Group A: 75 Group B: 75 | 75 | 0 | Group A: Oral N-acetylcysteine, 600 mg twice a day for five days + usual treatment  Group B: Bromhexine tablets, 8 mg three times a day for five days + usual treatment | Group C: Only usual treatment (Naproxen, 250 mg twice daily for five days, famotidine, 20 mg once daily for 10 days, vitamin D 50,000 per week for four weeks, vitamin C, 1000 mg daily) | Number of ICU admissions per hospitalized group | p-value (95% CI) | Group A vs. B vs. C:   4 (36.36%) vs. 1 (16.67%) vs. 11 (52.38%)   p-value = 0.095 | Note: ITT analysis was not explicitly mentioned in this trial. |
| rivaroxaban | Standard/Usual Care | Avezum, 2023, Brazil | RP | 327 | 330 | 0 | receive open-label rivaroxaban 10 mg OD for 14 days | routine care | Requirement of invasive mechanical ventillation | RR 95% CI | 1.10 (0.49-2.46) p = 0.835 |  |
| Sulodexide | Placebo | Gonzalez-Ochoa 2021, Mexico | RP | 124 | 119 | 0 | Sulodexide (oral 1,000 LRU/d) for 21 days | Placebo for 21 days | Invasive mechanical ventilation | p-value, RR (95% CI) | 0.47 (0.12–1.87), p = 0.29 |  |

**Table 4b:** Effect of antivirals on ICU admission and/or duration

| **Treatment Name** | **Comparator category** | **Study, country** | **Tx classification** | **Treatment (N)** | **Comparator (N)** | **Unique population** | **Tx dosage and duration** | **Type of comparator** | **Outcome Definition** | **Effect measure** | **Effect estimate** | **Notes** |
| --- | --- | --- | --- | --- | --- | --- | --- | --- | --- | --- | --- | --- |
| Lopinavir/ritonavir | Placebo | Kaizer, USA, 2023 | AV | 220 | 226 | 0 | Twice-daily oral LPV/r (400 mg/100 mg) for 14 days | matching placebo | Hospitalization rate for ICU admission over first 15 days | Tx vs comparator; Proportion (%) | 1 (0.5%) vs 0 | Hospitalized on mechanical ventilation/extracorporeal |
| Molnupiravir | Placebo | Johnson 2022, 107 sites in Africa, the Asia-Pacific region, Europe, Latin America, and North America | AV | 709 | 699 | 0 | Molnupiravir, 800 mg every 12 hours for 5 days | matching placebo every 12 hours for 5 days | Invasive Mechanical Ventilation through day 29 in the Modified ITT population | RRR, CI | 64.1% (−12.1% to 88.5%) | Modified ITT |
| Molnupiravir | Placebo | Johnson 2022, 107 sites in Africa, the Asia-Pacific region, Europe, Latin America, and North America | AV | 48 | 67 | 0 | Molnupiravir, 800 mg every 12 hours for 5 days | matching placebo every 12 hours for 5 days | Invasive Mechanical Ventilation through day 29 in the hospitalized Modified ITT population | RRR, CI | 49.2% (−49.9% to 82.8%) | n value used from Hospitalized MITT |
| Sofosbuvir + Daclatasvir + Standard/Usual Care; Sofosbuvir + Ravidasvir + Standard/Usual Care | Standard/Usual Care | Abbass 2021, Egypt | AV | SOF-DCV group  (N = 40 ) SOF-RDV group  (N = 40) | 40 | 0 | SOF 400 mg tablets plus DCV 60 mg tablets (SOF‐DCV) or  RDV 200 mg tablets (SOFRDV) in addition to standard treatment for 10 days. | Standard of care in Egyptian adults with COVID‐19.  The standard of care included: acetaminophen 500 mg tablets as needed and multivitamin supplements offered for all patients. | The percentage of patients admitted to ICU until the end of treatment and follow‐up period; SOF-DCV | Tx vs. comparator; N (%); p value | 3 (7.5%) vs 8 (20%); p = 0.09 |  |
| Sofosbuvir + Daclatasvir + Standard/Usual Care; Sofosbuvir + Ravidasvir + Standard/Usual Care | Standard/Usual Care | Abbass 2021, Egypt | AV | SOF-DCV group  (N = 40 ) SOF-RDV group  (N = 40) | 40 | 0 | SOF 400 mg tablets plus DCV 60 mg tablets (SOF‐DCV) or  RDV 200 mg tablets (SOFRDV) in addition to standard treatment for 10 days. | Standard of care in Egyptian adults with COVID‐19.  The standard of care included: acetaminophen 500 mg tablets as needed and multivitamin supplements offered for all patients. | The percentage of patients admitted to ICU until the end of treatment and follow‐up period; SOF-RDV | Tx vs. comparator; N (%); p value | 5 (12.5%) vs 8 (20%) (p = 0.273) |  |
| Sofosbuvir + Standard/Usual Care | Standard/Usual Care | Shoushtari, 2024, Iran | AV | 180 | 180 | 0 | Under usual treatment + sofosbuvir 400 mg, one pill daily for 7 days | Standard of care (under usual treatment + placebo one tablet daily for 7 days) | Frequency of hosptialization in the ICU | N vs N | 0 vs 0 | Note: ITT analysis was not explicitly mentioned in this trial. |

**Table 5a:** Effect of repurposed treatments on all-cause mortality.

| **Treatment Name** | **Comparator category** | **Study, country** | **Treatment classification** | **Treatment (N)** | **Comparator (N)** | **Unique population** | **Tx dosage and duration** | **Type of comparator** | **Outcome Definition** | **Effect measure** | **Effect estimate** | **Note** |
| --- | --- | --- | --- | --- | --- | --- | --- | --- | --- | --- | --- | --- |
| Amantadine | Placebo | Weis, 2023, Denmark | RP | 121 | 121 | 1 | Amantadine 100 mg twice daily for 5 days | Placebo twice daily for 5 days | Mortality during study duration (90 days) | Tx vs comparator, N vs N | 0 vs 0 | No mortality observed in either group |
| Aspirin + placebo; 2.5mg Apixaban; 5mg Apixaban | Placebo | Connors 2021, US | RP | Aspirin (n=144),  Prophylactic Apixaban (n=135),  Therapeutic Apixaban (n=143) | Placebo (n=136) |  | 1. Aspirin (81 mg orally once daily) with matching placebo. 2. Prophylactic-dose apixaban (2.5 mg orally twice daily. 3. Therapeutic-dose apixaban (5 mg orally twice daily)  For 45 days | Placebo twice daily | All-cause mortality for up to 45 days after treatment initiation. | No of deaths | 0 |  |
| Azithromycin | Standard/Usual Care | Hinks 2021 | RP | 145 | 147 | 0 | 500 mg Azithromycin once daily orally for 14 days | Standard care | all-cause mortality at day 28 | Tx vs comparator; p value, N (%) | 1/145 (1%) vs 1/147 (1%); p=1·00, |  |
| Bosentan | Placebo | Shahbazi, 2023, Iran | RP | 129 | 130 | 0 | intervention group received bosentan tab 62.5 mg twice daily and the placebo group received identical placebo in the same manner for 30 days | Placebo | Death from any cause within 30 days | Risk difference | -2.3% (-4.7 to 0.3) p = 0.24 |  |
| Budesonide | Standard/Usual Care | Yu, UK, 2021 | RP | 833 | 1126 | 1 | 800 μg Budesonide twice daily for 14 days | Usual care + other inventions | Proportion of participant death | Tx vs comparator; Percentage of mortality | 6 (0.72%) vs 11 (0.98%) |  |
| Camostat | Placebo | Jilg, 2023, USA | RP | 109 | 107 | 0 | 200 mg oral camostat (2 tablets, 100 mg each) every 6 hours for 7 days | 200 mg oral placebo (2 tablets, 100 mg each) every 6 hours for 7 days | Any deaths | N; Tx vs comparator | 1 vs 0 |  |
| Ciclesonide | Placebo | Ezer 2021, Canada | RP | 105 | 98 | 1 | inhaled ciclesonide (600 μg twice daily) and intranasal ciclesonide (200 μg daily) for 14 days. | metered dose inhaler and nasal saline placebos for 14 days | Moratlity at day 14 amd 29 | N,% | 0(0) |  |
| Colchicine | Placebo | Tardif 2021, Brazil, Canada, Greece, South Africa, Spain, and the USA | RP | 2235 | 2253 | 0 | 0·5 mg colchicine orally administered twice per day for the first 3 days and then  once per day for 27 days thereafter | Matching placebo | Death | Odds ratio  (95% CI) | 0·56 (0·19–1·67) |  |
| Colchicine | Standard/Usual Care | Eikelboom 2022, Brazil, Canada, Colombia, Ecuador, Egypt, India, Nepal, Pakistan, Phillippines, Russia, South Africa, United Arab Emirates | RP | 1939 | 1942 | 0 | oral colchicine 0·6 mg twice daily for 3 days and then 0.6 mg once daily for 25 days or control | usual care as established by local site investigator | Death | HR (95% CI) | 1·09 (0·48–2·47) |  |
| Colchicine | Standard/Usual Care | Eikelboom 2022, Brazil, Canada, Colombia, Ecuador, Egypt, India, Nepal, Pakistan, Phillippines, Russia, South Africa, United Arab Emirates | RP | 1939 | 1942 | 0 | oral colchicine 0·6 mg twice daily for 3 days and then 0.6 mg once daily for 25 days or control | usual care as established by local site investigator | Respiratory death | HR (95% CI) | 1.43 (0.54-3.75) |  |
| Domperidone + Standard/Usual Care | Placebo | Basalo, 2023, Spain | RP | 87 | 86 | 0 | Three daily doses of 10 mg (30 mg/day) of domperidone for 7 days | Placebo, three daily doses, for 7 days | Death at 28 days after completion of study | N | 0 |  |
| Fluvoxamine | Placebo | Stewart, 2023, USA | RP | 589 | 586 | 1 | 50 mg (one 50-mg tablet) twice daily for 1 day, followed by 100 mg (two 50-mg tablets) fluvoxamine | Matching placebo twice daily for 1 day (two 50-mg tablets) or matching placebo twice daily for 12 days, for a total 13-day course | Mortality at day 28 | N (%) vs. N(%); Tx vs comparator | 0 vs. 0 | Unique patient population: 30 years or older |
| Fluvoxamine | Standard/Usual Care | Wannigama 2024, Thailand | RP | 162 | 336 | 0 | The fluvoxamine-only arm received fluvoxamine (immediate release) 50 mg orally, one tablet in the morning and one tablet at bedtime for the first two days, then escalated to one 50 mg one tablet in the morning and two tablets before bedtime for days 3 through 12, ending with one 50 mg one tablet in the morning and one tablet at bedtime for days 13 and 14 | Standard care (based on COVID-19 treatment guidelines at the time of the trial) | Death at day 9 | N (%) | 0 vs 0 |  |
| Fluvoxamine | Standard/Usual Care | Wannigama 2024, Thailand | RP | 162 | 336 | 0 | The fluvoxamine-only arm received fluvoxamine (immediate release) 50 mg orally, one tablet in the morning and one tablet at bedtime for the first two days, then escalated to one 50 mg one tablet in the morning and two tablets before bedtime for days 3 through 12, ending with one 50 mg one tablet in the morning and one tablet at bedtime for days 13 and 15 | Standard care (based on COVID-19 treatment guidelines at the time of the trial) | Death at day 14 | N (%) | 0 vs 0 |  |
| Fluvoxamine | Standard/Usual Care | Wannigama 2024, Thailand | RP | 162 | 336 | 0 | The fluvoxamine-only arm received fluvoxamine (immediate release) 50 mg orally, one tablet in the morning and one tablet at bedtime for the first two days, then escalated to one 50 mg one tablet in the morning and two tablets before bedtime for days 3 through 12, ending with one 50 mg one tablet in the morning and one tablet at bedtime for days 13 and 16 | Standard care (based on COVID-19 treatment guidelines at the time of the trial) | Death at day 28 | N (%) | 0 vs 0 |  |
| Fluvoxamine | Placebo | Reis 2022, Brazil | RP | 741 | 756 | 0 | fluvoxamine (Luvox, Abbott) at a dose of 100 mg twice a day for 10 days starting directly after randomisation (day 1). | Corresponding placebo starting directly after randomisation (day 1). | Death, intention to treat | OR 95% CI | 0·69 (0·36–1·27) | Unadjusted odds ratio |
| Fluvoxamine + Bromhexine | Standard/Usual Care | Wannigama 2024, Thailand | RP | 178 | 336 | 0 | The fluvoxamine-only arm received fluvoxamine (immediate release) 50 mg orally, one tablet in the morning and one tablet at bedtime for the first two days, then escalated to one 50 mg one tablet in the morning and two tablets before bedtime for days 3 through 12, ending with one 50 mg one tablet in the morning and one tablet at bedtime for days 13 and 14. Plus one 8 mg bromhexine tablet twice daily for 10 days | Standard care (based on COVID-19 treatment guidelines at the time of the trial) | Death at day 9 | Tx vs comparator; N (%) | 0 vs 0 |  |
| Fluvoxamine + Bromhexine | Standard/Usual Care | Wannigama 2024, Thailand | RP | 178 | 336 | 0 | The fluvoxamine-only arm received fluvoxamine (immediate release) 50 mg orally, one tablet in the morning and one tablet at bedtime for the first two days, then escalated to one 50 mg one tablet in the morning and two tablets before bedtime for days 3 through 12, ending with one 50 mg one tablet in the morning and one tablet at bedtime for days 13 and 14. Plus one 8 mg bromhexine tablet twice daily for 10 days | Standard care (based on COVID-19 treatment guidelines at the time of the trial) | Death at day 14 | Tx vs comparator; N (%) | 0 vs 0 |  |
| Fluvoxamine + Bromhexine | Standard/Usual Care | Wannigama 2024, Thailand | RP | 178 | 336 | 0 | The fluvoxamine-only arm received fluvoxamine (immediate release) 50 mg orally, one tablet in the morning and one tablet at bedtime for the first two days, then escalated to one 50 mg one tablet in the morning and two tablets before bedtime for days 3 through 12, ending with one 50 mg one tablet in the morning and one tablet at bedtime for days 13 and 14. Plus one 8 mg bromhexine tablet twice daily for 10 days | Standard care (based on COVID-19 treatment guidelines at the time of the trial) | Death at day 28 | Tx vs comparator; N (%) | 0 vs 0 |  |
| Fluvoxamine + Budesonide | Placebo | Reis, Brazil, 2023 | RP | 738 | 738 | 0 | fluvoxamine (100 mg twice daily for 10 days) plus inhaled budesonide (800 mcg twice daily for 10 days) | matching placebos | Repored death due to COVID-19 | RR 95% CrI | 2.44 (0.18-76.66) |  |
| Fluvoxamine + Cyproheptadine | Standard/Usual Care | Wannigama 2024, Thailand | RP | 147 | 336 | 0 | The fluvoxamine-only arm received fluvoxamine (immediate release) 50 mg orally, one tablet in the morning and one tablet at bedtime for the first two days, then escalated to one 50 mg one tablet in the morning and two tablets before bedtime for days 3 through 12, ending with one 50 mg one tablet in the morning and one tablet at bedtime for days 13 and 14. Plus one 4 mg cyproheptadine tablet, three times a day for 14 days | Standard care (based on COVID-19 treatment guidelines at the time of the trial) | Death at day 9 | Tx vs comparator; N (%) | 0 vs 0 |  |
| Fluvoxamine + Cyproheptadine | Standard/Usual Care | Wannigama 2024, Thailand | RP | 147 | 336 | 0 | The fluvoxamine-only arm received fluvoxamine (immediate release) 50 mg orally, one tablet in the morning and one tablet at bedtime for the first two days, then escalated to one 50 mg one tablet in the morning and two tablets before bedtime for days 3 through 12, ending with one 50 mg one tablet in the morning and one tablet at bedtime for days 13 and 14. Plus one 4 mg cyproheptadine tablet, three times a day for 14 days | Standard care (based on COVID-19 treatment guidelines at the time of the trial) | Death at day 14 | Tx vs comparator; N (%) | 0 vs 0 |  |
| Fluvoxamine + Cyproheptadine | Standard/Usual Care | Wannigama 2024, Thailand | RP | 147 | 336 | 0 | The fluvoxamine-only arm received fluvoxamine (immediate release) 50 mg orally, one tablet in the morning and one tablet at bedtime for the first two days, then escalated to one 50 mg one tablet in the morning and two tablets before bedtime for days 3 through 12, ending with one 50 mg one tablet in the morning and one tablet at bedtime for days 13 and 14. Plus one 4 mg cyproheptadine tablet, three times a day for 14 days | Standard care (based on COVID-19 treatment guidelines at the time of the trial) | Death at day 28 | Tx vs comparator; N (%) | 0 vs 0 |  |
| Fluvoxamine + favipiravir; Fluvoxamine + favipiravir + dexamethasone | Favipiravir | Siripongboonsitti, 2023, Thailand | RP | 134 | 132 | 1 | 50 mg twice daily of fluvoxamine for 10 days and favipiravir; patients with moderate illness also received 6 mg per day of dexamethasone for 10 days | Each group received 3600 mg of FPV loading dose on day 0 and 1600 mg per day of FPV from days 1-4 in mild COVID-19 and days 1-9 in moderate COVID-19. In a patient with obesity, the FPV loading dose was 4800 mg on day 0, then 2000 mg per day of FPV on days later | Mortality, mild illness | Tx vs comparator, N vs N, p-value | 0 vs 0, p = 1.000 |  |
| Fluvoxamine + favipiravir; Fluvoxamine + favipiravir + dexamethasone | Favipiravir | Siripongboonsitti, 2023, Thailand | RP | 134 | 132 | 1 | 50 mg twice daily of fluvoxamine for 10 days and favipiravir; patients with moderate illness also received 6 mg per day of dexamethasone for 10 days | Each group received 3600 mg of FPV loading dose on day 0 and 1600 mg per day of FPV from days 1-4 in mild COVID-19 and days 1-9 in moderate COVID-19. In a patient with obesity, the FPV loading dose was 4800 mg on day 0, then 2000 mg per day of FPV on days later | Mortality, moderate illness | Tx vs comparator, N vs N, p-value | 0 vs 0, p = 1.000 |  |
| Fluvoxamine + Standard/Usual Care | Placebo + Standard/Usual Care | Ibrahim, 2023, Egypt | RP | 37; moderate: 51 | 35; moderate: 39 | 0 | standard therapy plus fluvoxamine at a dose of 100 mg twice daily for 10 days. | favipiravir (nonspecific antiviral drug) plus nonspecific antibiotics as a standard regimen in our study according to local recommendations | Mortality at day 30 | Not reported | Not reported | Mild: non-pneumonic; Moderate: pneumonic |
| Hydroxychloroquine | Placebo | Spivak, 2023, USA | RP | 185 | 182 | 0 | Participants randomized to the HCQ arm received 400 mg HCQ by mouth twice daily for 1 day and then 200 mg by mouth twice daily for 4 days. | Matching placebo | Deaths | N vs N | 0 vs 0 |  |
| Hydroxychloroquine | Standard/Usual Care | Mitja 2021, Spain | RP | 136 | 157 | 0 | HCQ - Dolquine® 800 mg on day 1, followed by 400 mg once daily for six days) | Usual care | No of deaths | n | 0 deaths |  |
| Hydroxychloroquine | Placebo | Avezum, Brazil, 2022 | RP | 689 | 683 | 1 | hydroxychloroquine 400 mg twice a day in the first day, 400 mg once daily thereafter for a total of seven days | Placebo twice daily in the first day, then once daily thereafter for a total of seven days | Death within 30 days | Hazard Ratio (HR) | 1.56 [ 0.42–6.72], p = 0.54 |  |
| Hydroxychloroquine | Placebo | Schwartz 2021 | RP | 111 | 37 | 0 | hydroxychloroquine dose was 800 mg orally in divided  doses on day 1 followed by 200 mg twice daily for 4 days | identical matching placebo (12 tablets over 5 days) | Mortality within 30 d | N% | 0 |  |
| Ivermectin | Placebo | Abbas 2022, China | RP | 99 | 103 | 0 | ivermectin, 300 μg/ kg body weight per day for 5 d | placebo | Mortality | Apgar score | 1 vs 1 |  |
| Ivermectin | Placebo | Naggie, 2022, US | RP | 817 | 774 | 0 | 400 μg/kg daily for 3 days | Placebo | Mortality at day 28 | N tx vs placebo | 1 vs 0 |  |
| Ivermectin | Placebo; Fluvoxamine | Naggie, 2023, US | RP | 708 | 724 | 0 | dosing schedule was based on weight ranges as follows: those weighing 35 to 52 kg received a 21-mg daily dose Ivermectin; 53 to 69 kg, 28-mg daily dose; 70 to 89 kg, 42-mg daily dose; 90 to 109 kg, 49-mg daily dose; 110 to 129 kg, 56-mg daily dose; and more than 129 kg, 70-mg daily dose | placebo, fluvoxamine 50mg twice daily | Mortality at day 28 | tx: vs comparator | 1 vs 0 | c CI rather than CrI because low event rate precluded covariate adjustment. |
| Ivermectin | Placebo | Reis 2022, Brazil | RP | 679 | 679 | 0 | Ivermectin 400 microgram/kg for 3 days, once per day | placebos that were used in the trial involved regimens of 1, 3, 10, or 14 days in duration, according to the various comparator groups in the trial at the time of randomization | Death within 28 days | RR, 95% CI | 0.88 (0.49 to 1.55) |  |
| Ivermectin | Placebo | Lopez-Medina 2021, Columbia | RP | 200 | 198 | 0 | Ivermectin, 300 μg/kg of body weight per day for 5 days | Placebo 300 μg/kg of body weight per day for 5 days | no of deaths | Tx vs comparator; N (%) | 0 vs 1 (0.5) |  |
| Ivermectin | Placebo | Mirahmadizadeh 2022, Iran | RP | Ivermectin (S) = 130, Ivermectin (D) = 131 | Placebo = 130 | 0 | Single dose of ivermectin (3 mg tablet, cumulative dose of 12 mg, Stat. placebo tablets, at the second day) and double-dose ivermectin (3 mg tablet for 2 days, cumulative dose of 24 mg) or placebo at the day of RT-PCR-positive testing (enrolment day) and the next day. | Placebo | Mortality | N% | 0 | Ivermectin (Single Dose =S) Ivermectin (Double dose = D) |
| Ivermectin + Standard/Usual Care | Placebo + Standard/Usual Care | Vallejos 2021, Argentina | RP | 250 | 251 | 1 | Ivermectin plus standard of care (SOC) from randomization to the End Of Study (EOS) visit.  X tablets of 6 mg each at inclusion and another X tablets of 6 mg each 24 h after the first dose.  (The dose of ivermectin used was the approved dose in Argentina for the treatment of other diseases, such as parasitic diseases, and it was staggered according to weight.) | Placebo plus Standard of care (SOC)   (The SOC was in accordance with the recommendations of the Argentine Ministry of Health) | All-cause mortality | OR (95%CI) | 1.34, ( 0.30–6.07); p = 0.72 |  |
| Losartan | Placebo | Puskarich, 2021 | RP | 58 | 59 | 0 | Losartan 25mg, self-administered orally twice daily for 10 days for participants with eGFR >60 mL/min/1.73 m2, and once daily for those with eGFR 30–60 mL/min/1.73 m2. | 1:1 placebo | Death | difference in proportion, 95% confidence interval | 0.0 (-6.8, 6.7) | No deaths recorded |
| Metformin | Placebo | Reis, Brazil, 2022 | RP | 215 | 203 | 1 | Metformin (750 mg twice daily for 10 days) | Placebo tablets: twice diaily for 10 days | Proportion of fatilities | Tx vs comparator; N (%) p-value | 7 (3.3%) vs 9 (4.4%), p = 0.53 |  |
| Metformin; Ivermectin; Fluvoxamine | Placebo + Control | Bramante 2022, United States | RP | Metformin = 663 , Ivermectin = 410 , Fluvoxamine = 334 | Metformin control= 660, Ivermectin control= 398, Fluvoxamine control = 327 | 0 | immediate-release metformin administered with an increase in dose over 6 days to 1500 mg per day for 14 days, ivermectin at a dose of 390 to 470 μg per kilogram per day for 3 days, and fluvoxamine at a dose of 50 mg twice daily for 14 days. | The metformin control group included the patients who had received placebo, fluvoxamine alone, or ivermectin alone. Patients in the ivermectin control group were randomly assigned to receive either placebo or metformin alone. The fluvoxamine and fluvoxamine control groups were constructed similarly. The control groups that were used in the comparisons with the active-drug groups included only concurrently randomly assigned patient | Death; Metformin | Adjusted Odds Ratio (95% CI) | 0 | Modified ITT population. Adjusted odds ratios and 95% confidence intervals are based on a logistic-regression model that was adjusted for baseline vaccination status and the receipt of other medications during the trial; multiple imputation was used with chained equations and predictive mean matching. |
| Metformin; Ivermectin; Fluvoxamine | Placebo + Control | Bramante 2022, United States | RP | Metformin = 663 , Ivermectin = 410 , Fluvoxamine = 335 | Metformin control= 660, Ivermectin control= 398, Fluvoxamine control = 328 | 0 | immediate-release metformin administered with an increase in dose over 6 days to 1500 mg per day for 14 days, ivermectin at a dose of 390 to 470 μg per kilogram per day for 3 days, and fluvoxamine at a dose of 50 mg twice daily for 14 days. | The metformin control group included the patients who had received placebo, fluvoxamine alone, or ivermectin alone. Patients in the ivermectin control group were randomly assigned to receive either placebo or metformin alone. The fluvoxamine and fluvoxamine control groups were constructed similarly. The control groups that were used in the comparisons with the active-drug groups included only concurrently randomly assigned patient | Death; Ivermectin | Adjusted Odds Ratio (95% CI) | 0 | Modified ITT population. Adjusted odds ratios and 95% confidence intervals are based on a logistic-regression model that was adjusted for baseline vaccination status and the receipt of other medications during the trial; multiple imputation was used with chained equations and predictive mean matching. |
| Metformin; Ivermectin; Fluvoxamine | Placebo + Control | Bramante 2022, United States | RP | Metformin = 663 , Ivermectin = 410 , Fluvoxamine = 336 | Metformin control= 660, Ivermectin control= 398, Fluvoxamine control = 329 | 0 | immediate-release metformin administered with an increase in dose over 6 days to 1500 mg per day for 14 days, ivermectin at a dose of 390 to 470 μg per kilogram per day for 3 days, and fluvoxamine at a dose of 50 mg twice daily for 14 days. | The metformin control group included the patients who had received placebo, fluvoxamine alone, or ivermectin alone. Patients in the ivermectin control group were randomly assigned to receive either placebo or metformin alone. The fluvoxamine and fluvoxamine control groups were constructed similarly. The control groups that were used in the comparisons with the active-drug groups included only concurrently randomly assigned patient | Death; Fluvoxamine | Adjusted Odds Ratio (95% CI) | 0 | Modified ITT population. Adjusted odds ratios and 95% confidence intervals are based on a logistic-regression model that was adjusted for baseline vaccination status and the receipt of other medications during the trial; multiple imputation was used with chained equations and predictive mean matching. |
| Methylprednisolone | Standard/Usual Care | Issak, 2023, Egypt | RP | 377 | 375 | 0 | methylprednisolone 30 mg daily for 1 week in moderate cases, then reduced gradually over 2 weeks, mild cases were started at 15mg | standard COVID-19 protocol | Mortality at 28 days | OR 95% CI, Tx vs comparator, N vs N | 3 (0.80%) vs 11 (2.93%) p =0.043 0.27 ( 0.07 to 0.95) |  |
| Niclosamide + Bromhexine | Standard/Usual Care | Wannigama 2024, Thailand | RP | 172 | 336 | 0 | Participants received a 1000 mg of niclosamide tablet two times daily for a total of 14 days, in combination with one 8 mg tablet of bromhexine twice daily for a total of 10 days | Standard care (based on COVID-19 treatment guidelines at the time of the trial) | Death at day 9 | Tx vs comparator; N (%) | 0 vs 0 |  |
| Niclosamide + Bromhexine | Standard/Usual Care | Wannigama 2024, Thailand | RP | 172 | 336 | 0 | Participants received a 1000 mg of niclosamide tablet two times daily for a total of 14 days, in combination with one 8 mg tablet of bromhexine twice daily for a total of 10 days | Standard care (based on COVID-19 treatment guidelines at the time of the trial) | Death at day 14 | Tx vs comparator; N (%) | 0 vs 0 |  |
| Niclosamide + Bromhexine | Standard/Usual Care | Wannigama 2024, Thailand | RP | 172 | 336 | 0 | Participants received a 1000 mg of niclosamide tablet two times daily for a total of 14 days, in combination with one 8 mg tablet of bromhexine twice daily for a total of 10 days | Standard care (based on COVID-19 treatment guidelines at the time of the trial) | Death at day 28 | Tx vs comparator; N (%) | 0 vs 0 |  |
| Oral N-acetylcysteine + Standard/Usual Care; Bromhexine tablets + Standard/Usual Care | Standard/Usual Care | Eslami-Ghayour, 2023, Iran | RP | Group A: 75 Group B: 75 | 75 | 0 | Group A: Oral N-acetylcysteine, 600 mg twice a day for five days + usual treatment  Group B: Bromhexine tablets, 8 mg three times a day for five days + usual treatment | Group C: Only usual treatment (Naproxen, 250 mg twice daily for five days, famotidine, 20 mg once daily for 10 days, vitamin D 50,000 per week for four weeks, vitamin C, 1000 mg daily) | Mortality rates | N, %; Group A vs. B vs. C: | 0 vs. 0 vs. 7 (9.33%) | Note: ITT analysis was not explicitly mentioned in this trial. |
| Raloxifene | Placebo | Nicastri, 2022, Italy | RP | 60 mg group: 22  120 mg group: 20 | 19 | 0 | On day one of treatment, two oral doses were administered (one dose in the morning and one dose in the evening). Thereafter, a single daily oral dose was administered by patients: raloxifene 60 mg, raloxifene 120 mg or placebo for a maximum 14 days | On day one of treatment, two oral doses were administered (one dose in the morning and one dose in the evening). Thereafter, a single daily oral dose was administered by patients: raloxifene 60 mg, raloxifene 120 mg or placebo for a maximum 14 days | Deaths | N | 0 | None of the patients in any treatment group were admitted to the ICUI after randomization at any timepoint, and ther were also no deaths in any treatment or placebo group. |
| Rivaroxaban | Placebo | Ananworanich, 2022, USA | RP | 192 | 199 | 0 | one 10 mg Rivaroxaban tablet, daily, for 21 days | one 10 mg multivitamin placebo tablet, daily, for 21 days | Deaths | N (%) vs. N(%) | 0 vs 0 | modified ITT population |
| Rivaroxaban | Placebo | Piazza, 2023, USA | RP | 641 | 643 | 1 | 10 mg of oral rivaroxaban daily for 35 days | 10 mg of placebo daily for 35 days | All cause mortality | N (%) vs. N(%)  HR/RR (95% CI) | 2 (0.3%) vs. 2 (0.3%)   1.00 (0.14-7.10) |  |
| rivaroxaban | Standard/Usual Care | Avezum, 2023, Brazil | RP | 327 | 330 | 0 | receive open-label rivaroxaban 10 mg OD for 14 days | routine care | death not attributed to major injury within 30 days from randomisation | RR 95% CI | 0.67 (0.24–1.87) p = 0.603 |  |
| Sulodexide | Placebo | Gonzalez-Ochoa 2021, Mexico | RP | 124 | 119 | 0 | Sulodexide (oral 1,000 LRU/d) for 21 days | Placebo for 21 days | Mortality | p-value, RR (95% CI) | 0.41 (0.10–1.55), p = 0.19 |  |

**Table 5b:** Effect of antivirals on all-cause mortality

| **Treatment Name** | **Comparator category** | **Study, country** | **Tx classification** | **Treatment (N)** | **Comparator (N)** | **Unique population** | **Tx dosage and duration** | **Type of comparator** | **Outcome Definition** | **Effect measure** | **Effect estimate** | **Note** |
| --- | --- | --- | --- | --- | --- | --- | --- | --- | --- | --- | --- | --- |
| Bemnifosbuvir | Placebo | Horga 2023, Multiple | AV | 137 | 70 | 0 | Oral bemnifosbuvir 550 mg (two 275 mg tablets a day), for 5 days | Placebo treatment, 275 mg, two times a day, for 5 days | Proportion of patients with all-cause mortality | Median (97.5% CI from Kaplan-Meier curves), n | 0 vs. 0 |  |
| Ensitrelvir | Placebo | Yotsuyanagi, 2024, Japan, Vietnam, and South Korea | AV | Group A:  347  Group B: 340  Group D: 603  Group E:  595 | Group C:  343  Group F:  600 | 0 | Group A & D:  375 mg oral ensitrelvir on day one and 125 mg, once-daily, days 2-5  Group B and E: 375 mg oral ensitrelvir on day one and 250 mg, once-daily, days 2-5 | Group C and F:  Oral placebo tablets days 1-5 | Adverse events leading to death | N; Only assesses Group A , B, and C | 0 vs 0 vs 0 |  |
| Favipriavir | Placebo | Iwata, 2023, Japan | AV | 41 | 43 | 1 | 1800 mg of favipiravir per dose twice a day on Day 1 followed by 800 mg favipiravir per dose twice a day on Days 2 through 10 | Matching placebo twice daily for 10 days | Adverse events leading to death | Tx vs comparator; N 95% CI | 0 (95% CI 0.0, 8.6) vs  0 (95% CI 0.0, 8.2) | Unique patient population:  Adult patients with one or more of the following risk factors were eligible: age ≥65 years, malignant tumor, chronic obstructive pulmo nary disease or other chronic lung disease, cardiovascular disease, ce rebrovascular disorder, chronic kidney disease, type 2 diabetes mellitus, hypertension or dyslipidemia, obesity (BMI ≥30 kg/m2 ), or active smoking |
| Favipriavir | Placebo | Bosaeed 2022, Saudi Arabia | AV | 112 | 119 | 0 | favipiravir 1800 mg (nine tablets) twice daily as a loading dose on day 1 followed by 800 mg (four tablets) twice daily as a maintenance dose for a total duration of 5 to 7 days of therapy or matching placebo | placebo | mortality at 28 days | Tx vs comparator; N vs N, p-val | 6 (5.3%) vs 2 (1.6%), p-value = 0.16 |  |
| Leriterelvir | Placebo | Zhan, 2024, China | AV | 680 | 679 | 0 | Leritrelvir, 400 mg, three times a day orally for 5 days | Placebo, three times a day orally for 5 days | All-cause mortality by day 29 | N (%); Tx vs comparator | 0 vs 0 |  |
| Lopinavir/ritonavir | Placebo | Kaizer, USA, 2023 | AV | 220 | 226 | 0 | Twice-daily oral LPV/r (400 mg/100 mg) for 14 days | matching placebo | All-cause mortality through day 29 | Tx vs comparator; Proportion (%) | 1 (0.5%) vs 0 |  |
| Molnupiravir | Placebo | JaykBernal 2022, Multiple countries (USA, Ukraine, Taiwan, UK, Spain, South Africa, Russian Federation, Philippines, Mexico, Japan, Italy, Guatemala, Germany, France, Egypt, Colombia, Chilie, Canada, Brazil, Argentina) | AV | 709 | 699 | 0 | 800 mg of molnupiravir twice daily for 5 days | placebo twice daily for 5 days | Reduction in risk of death through day 29 | %, 95% CI | 89%, 14 to 99 |  |
| Sofosbuvir + Daclatasvir + Standard/Usual Care; Sofosbuvir + Ravidasvir + Standard/Usual Care | Standard/Usual Care | Abbass 2021, Egypt | AV | SOF-DCV group  (N = 40 ) SOF-RDV group  (N = 40) | 40 | 0 | SOF 400 mg tablets plus DCV 60 mg tablets (SOF‐DCV) or  RDV 200 mg tablets (SOFRDV) in addition to standard treatment for 10 days. | Standard of care in Egyptian adults with COVID‐19.  The standard of care included: acetaminophen 500 mg tablets as needed and multivitamin supplements offered for all patients. | Death cases in all 3 groups. | Comparator vs tx vs tx; overall p value, N% | P = 0.329, N% (SOC = 6 (15%), SOF‐RDV = 4 (10%),  SOF‐DCV = 2 (5%)) |  |
| Sofosbuvir + Standard/Usual Care | Standard/Usual Care | Shoushtari, 2024, Iran | AV | 180 | 180 | 0 | Under usual treatment + sofosbuvir 400 mg, one pill daily for 7 days | Standard of care (under usual treatment + placebo one tablet daily for 7 days) | Mortality rate | N vs N | 0 vs 0 | Note: ITT analysis was not explicitly mentioned in this trial. |
| SNG001 | Placebo | Jagannathan, 2023, US | RP | 110 | 110 | 0 | SNG001, 12 MIU/mL, single nebulized dose, once a day, for 14 days | Placebo solution, single nebulized dose, once day, for 14 days | Treatment-emergent adverse events through day 28 - Grade 5 (death) | N (%); Tx vs comparator | 0 vs 0 |  |
