# Supplementary material for: Effectiveness of oral and inhaled repurposed and antiviral pharmaceutical treatments for acute SARS-CoV-2 in outpatient settings: a systematic review of 124 randomized trials
Source: BMC Infect Dis. 2026 Jul 23;26:1829. doi: 10.1186/s12879-026-13901-7 (PMC13682097; doi:10.1186/s12879-026-13901-7)
Supplement: Supplementary file 4 [file 12879_2026_13901_MOESM2_ESM.docx]

**Supplementary File 2. Risk of Bias 2.0 Assessment**
